# Supplementary material for: Diabetes drugs activate neuroprotective pathways in models of neonatal hypoxic-ischemic encephalopathy
Source: EMBO Mol Med. 2024 May 23;16(6):5. doi: 10.1038/s44321-024-00079-1 (PMC11178908; doi:10.1038/s44321-024-00079-1)
Supplement: Supplementary file 1 — Appendix [file 44321_2024_79_MOESM1_ESM.pdf]

Appendix

Table of Contents

Appendix Table S1 and legend……………………………………………Page 1

Appendix Table S2 and legend…………………………………………….Page1

Appendix Table S1 – Primer sequences for qPCR.

Target Gene	Sequence	Reference
GAPDH	F GTTGTCTCCTGCGACTTCA<br>R GGTGGTCCAGGGTTTCTTA	Pick primers
CREB	F CACAGAACCAGTTTCCATCATCCAGT<br>R CATGTTCAGAGGGTTAGGGAGAGCA	Miller, 2012
Gsk3β	F TTGGACAAAGGTCTTCCGGC<br>R AAGAGTGCAGGTGTGTCTCG	Li, 2015
GLP1-R	F AGACGGTGCAGAAATGGAGA<br>R TGGCGCTTCCGTGAGG	During, 2003
ATF-3	F TTACCGTCAACAACAGACCC<br>R TCAGCTCAGCATTCACACTC	Edagawa, 2014
BCl2	F GTGGTGGAGGAACTCTTCAG<br>R GTTCCACAAAGGCATCCCAG	Wenyuan, 2016
BClxL	F CGGAGAGCGTTCAGTGATCTA<br>R TTGTCTACGCTTTCCACGCA	Pick primers

Appendix Table S2 – Summary of the primary antibodies used for immunohistochemistry.

Antibody	Reference	Dilution	Protocol
CD68	MCA1957	1:2000	IHC
NeuN	MAB377	1:500	IHC
Iba-1	ab5076	1:500	IHC
GFAP	MAB3402	1:2000	IHC
pCREB Ser133	CS #9198	1:1000	WB
pGSK3β Ser 9	CS #9336	1:500	WB
pGSK3β Try216	Ab75745	1:1000	WB
βactin	Ab8226	1:5000	WB
