# Supplementary figures and images for: Diabetes drugs activate neuroprotective pathways in models of neonatal hypoxic-ischemic encephalopathy
Source: EMBO Mol Med. 2024 May 23;16(6):5. doi: 10.1038/s44321-024-00079-1 (PMC11178908; doi:10.1038/s44321-024-00079-1)

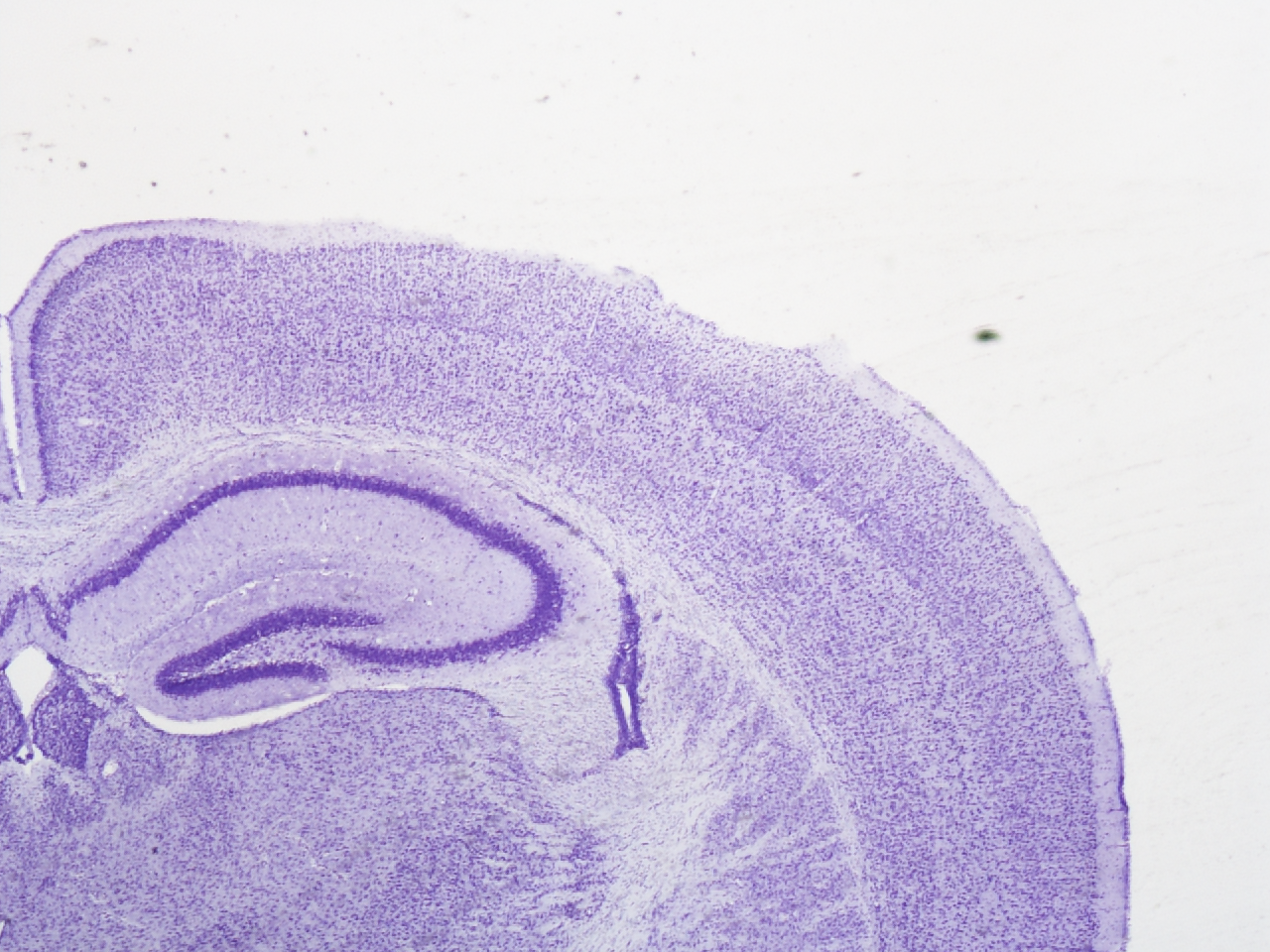

Supplement: Supplementary file 5 — Source data Fig. 4 [file 44321_2024_79_MOESM5_ESM.zip › Figure 4/4D/HI+Sema insert.TIF]

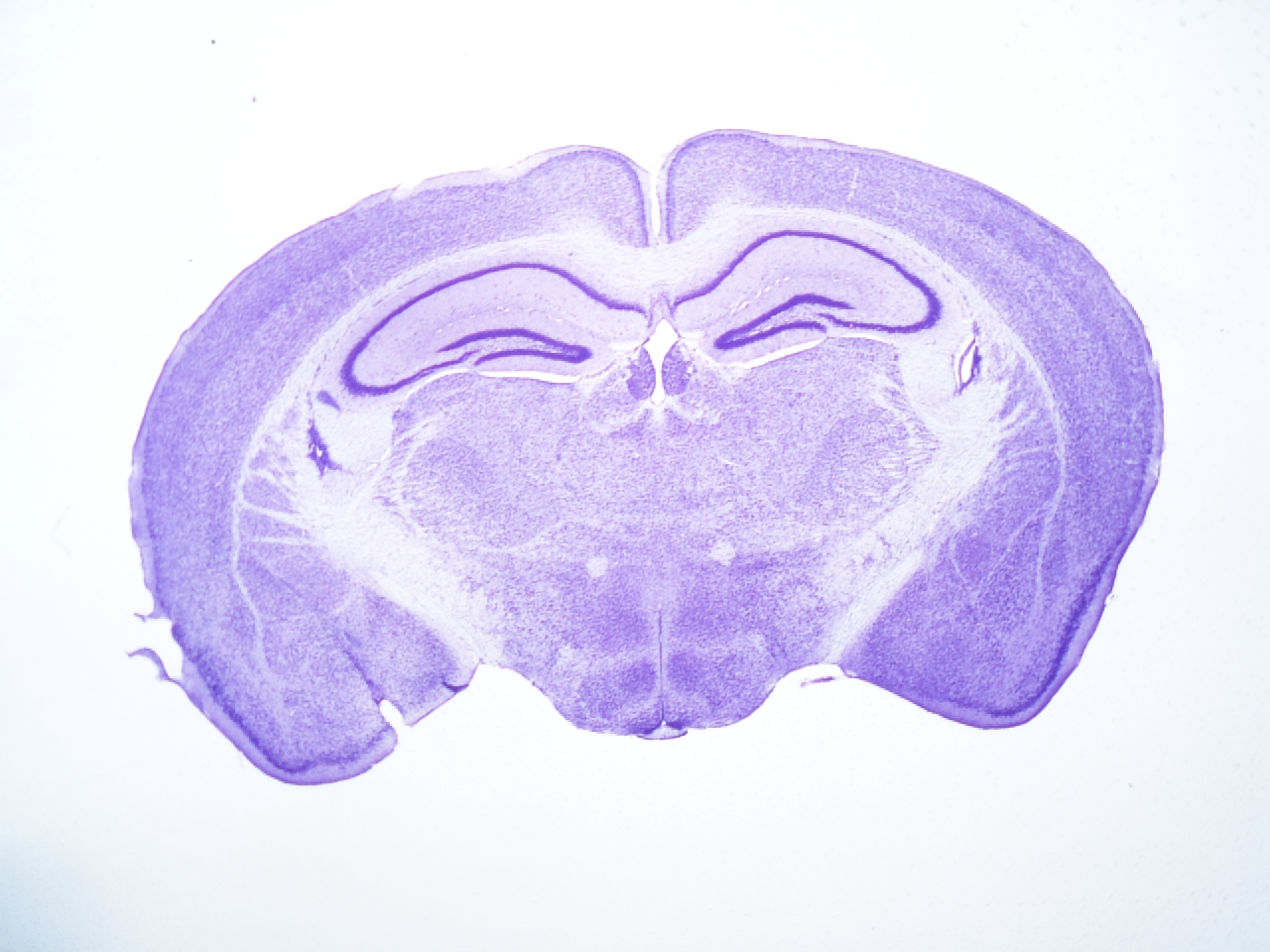

Supplement: Supplementary file 5 — Source data Fig. 4 [file 44321_2024_79_MOESM5_ESM.zip › Figure 4/4D/Sal whole brain.TIF]

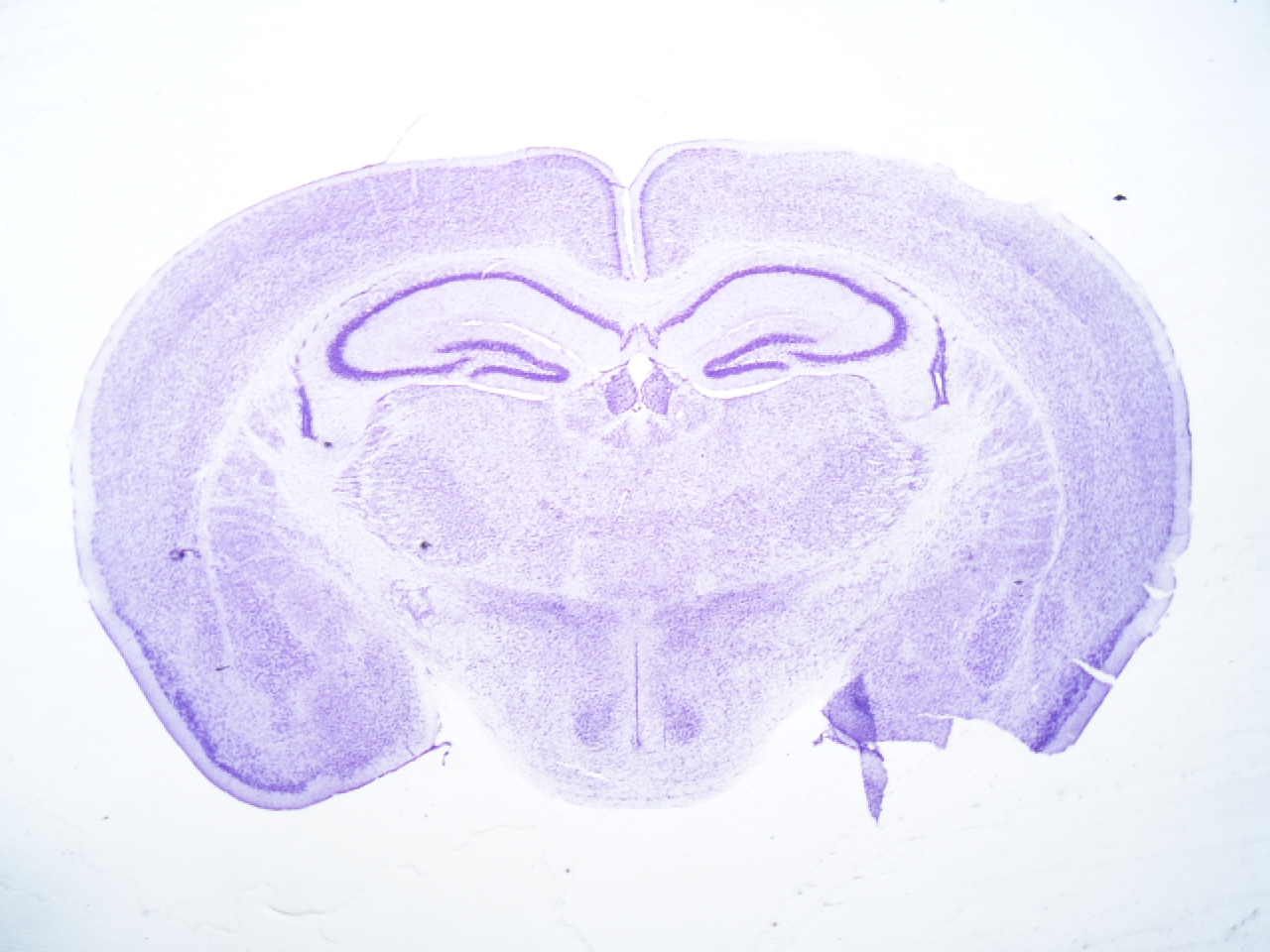

Supplement: Supplementary file 5 — Source data Fig. 4 [file 44321_2024_79_MOESM5_ESM.zip › Figure 4/4D/HI+Sema whole brain.TIF]

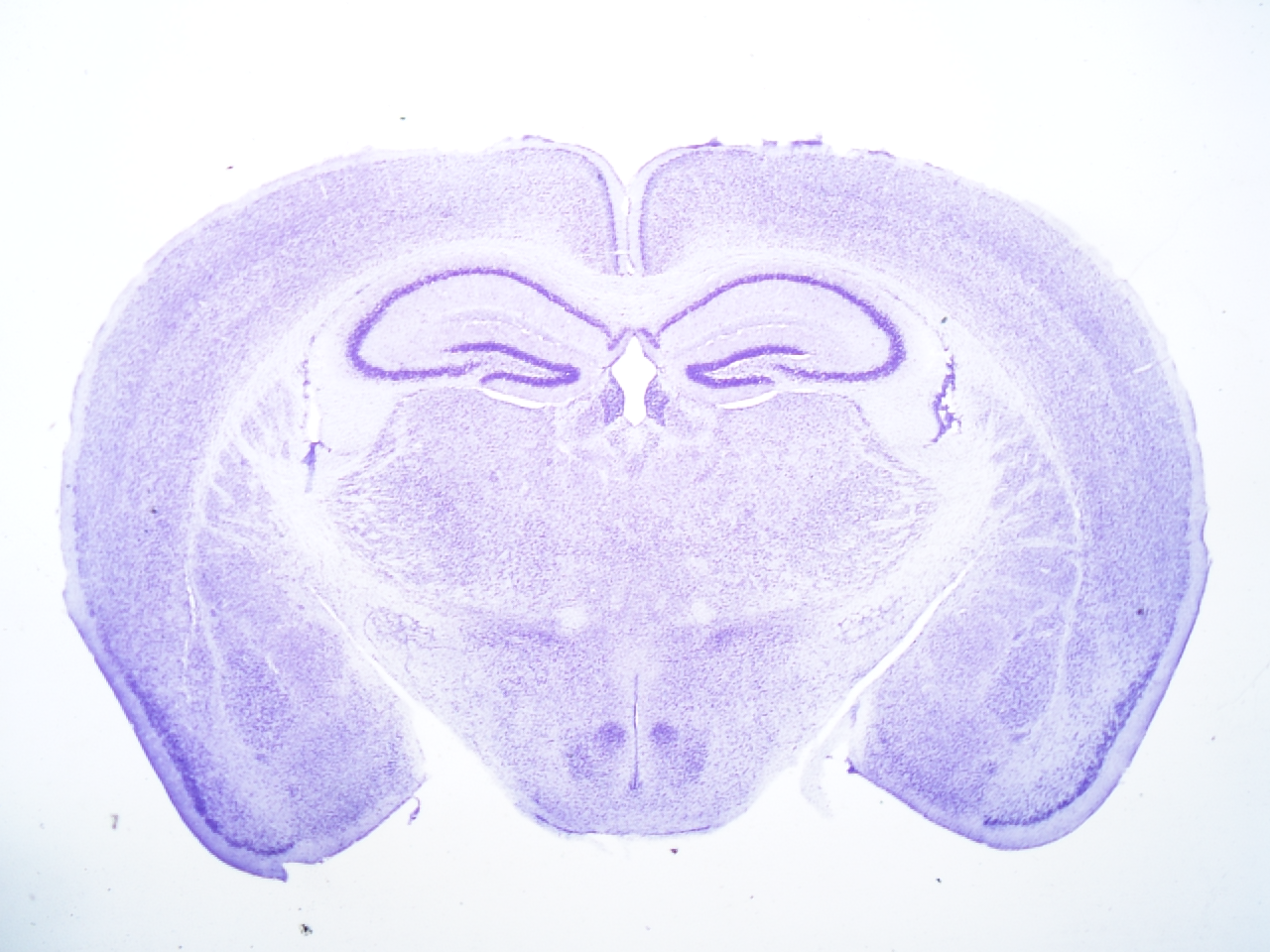

Supplement: Supplementary file 5 — Source data Fig. 4 [file 44321_2024_79_MOESM5_ESM.zip › Figure 4/4D/HI+Ex-4 whole brain.TIF]

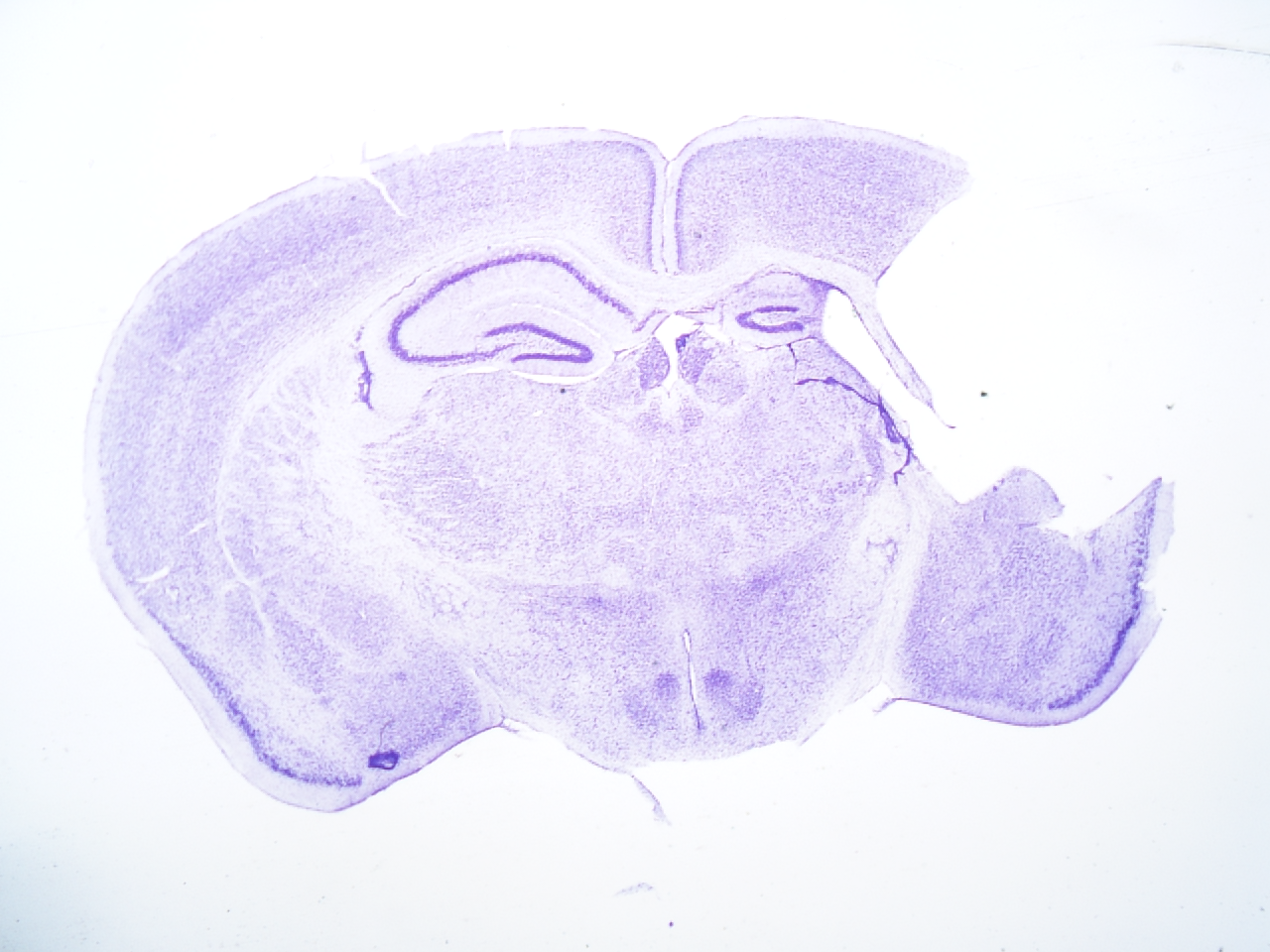

Supplement: Supplementary file 5 — Source data Fig. 4 [file 44321_2024_79_MOESM5_ESM.zip › Figure 4/4D/HI+Sal whole brain.TIF]

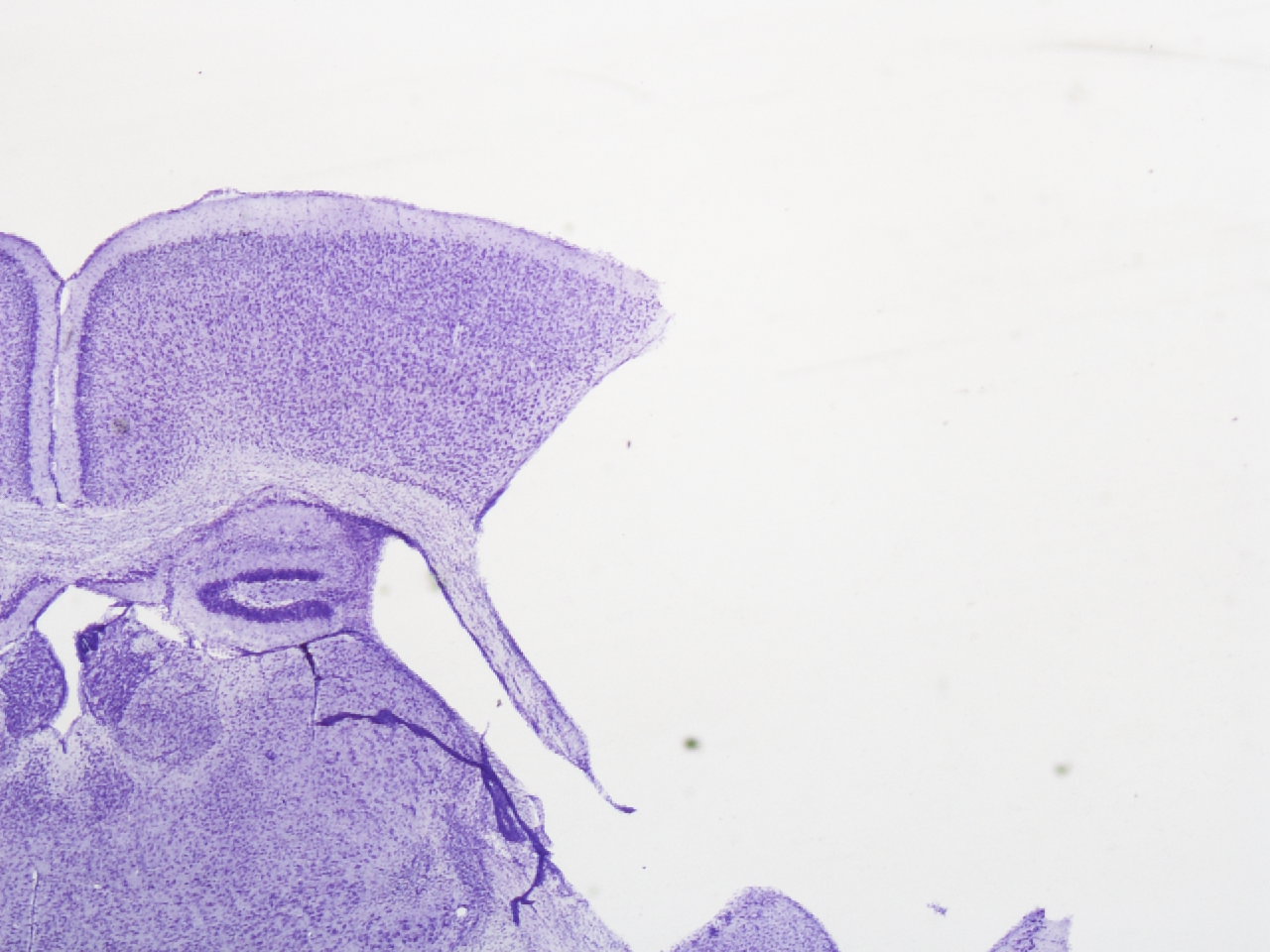

Supplement: Supplementary file 5 — Source data Fig. 4 [file 44321_2024_79_MOESM5_ESM.zip › Figure 4/4D/HI+Sal insert.TIF]

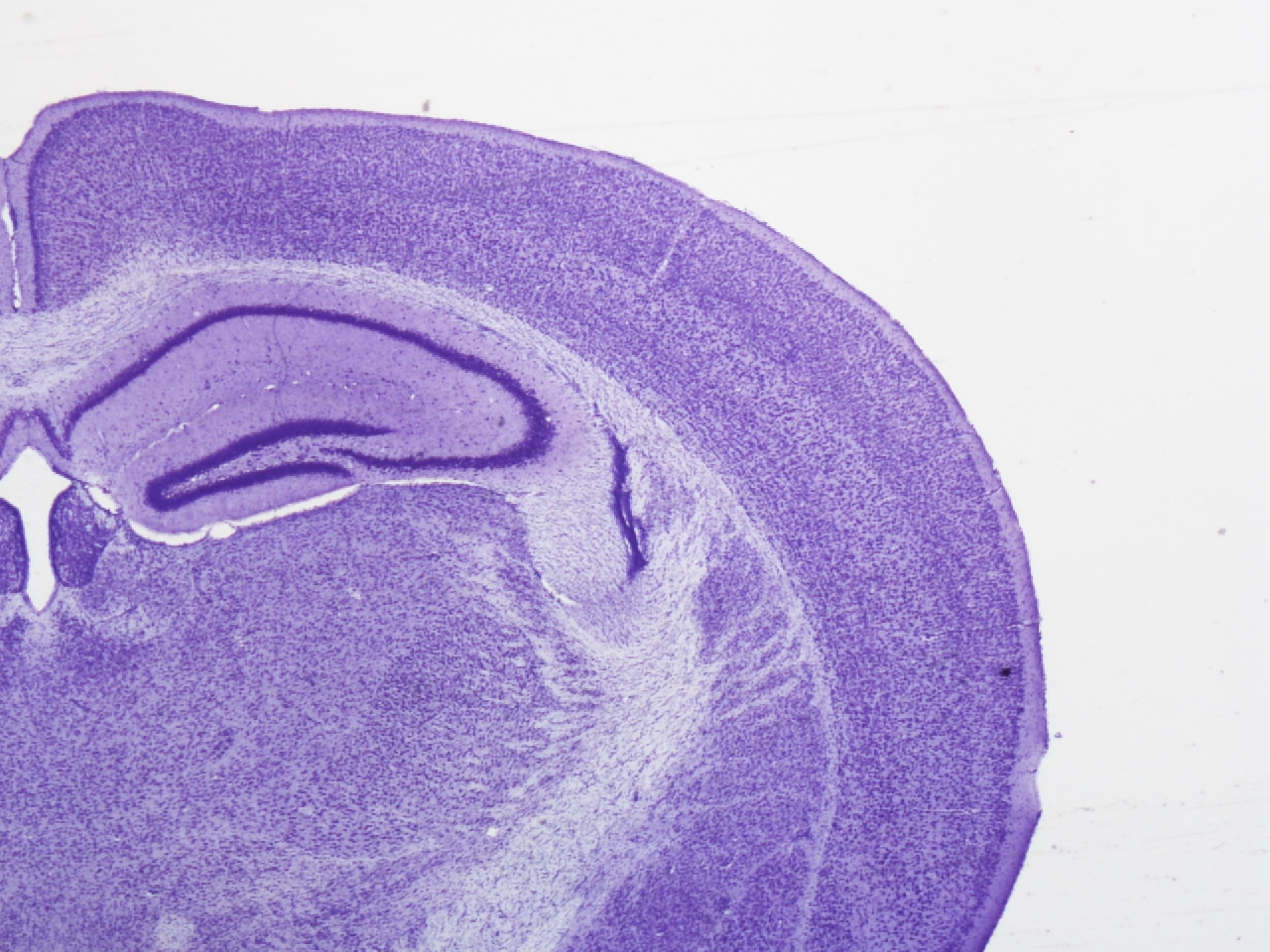

Supplement: Supplementary file 5 — Source data Fig. 4 [file 44321_2024_79_MOESM5_ESM.zip › Figure 4/4D/Sal insert.TIF]

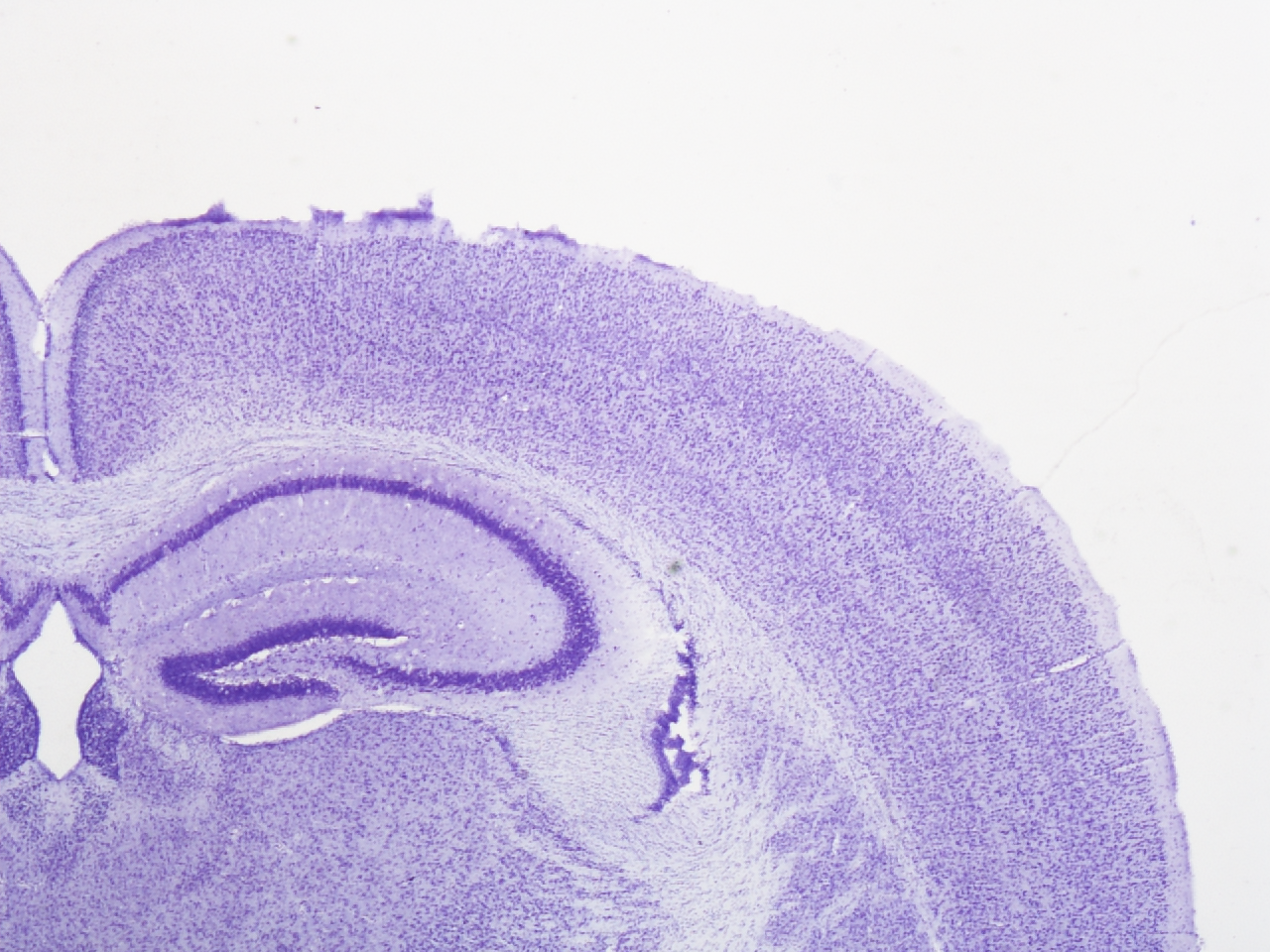

Supplement: Supplementary file 5 — Source data Fig. 4 [file 44321_2024_79_MOESM5_ESM.zip › Figure 4/4D/HI+Ex-4 insert.TIF]

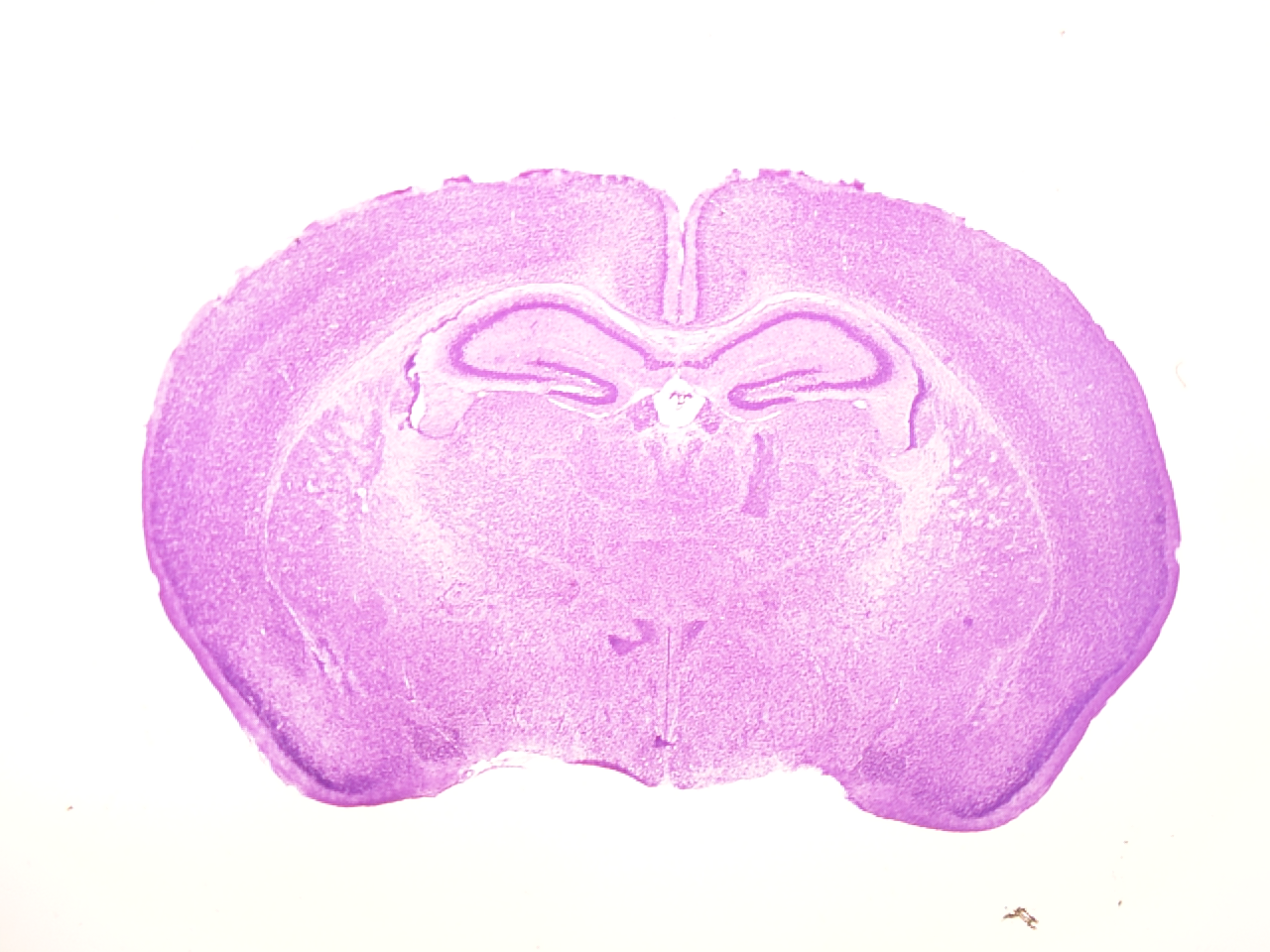

Supplement: Supplementary file 5 — Source data Fig. 4 [file 44321_2024_79_MOESM5_ESM.zip › Figure 4/4A/Saline whole brain.TIF]

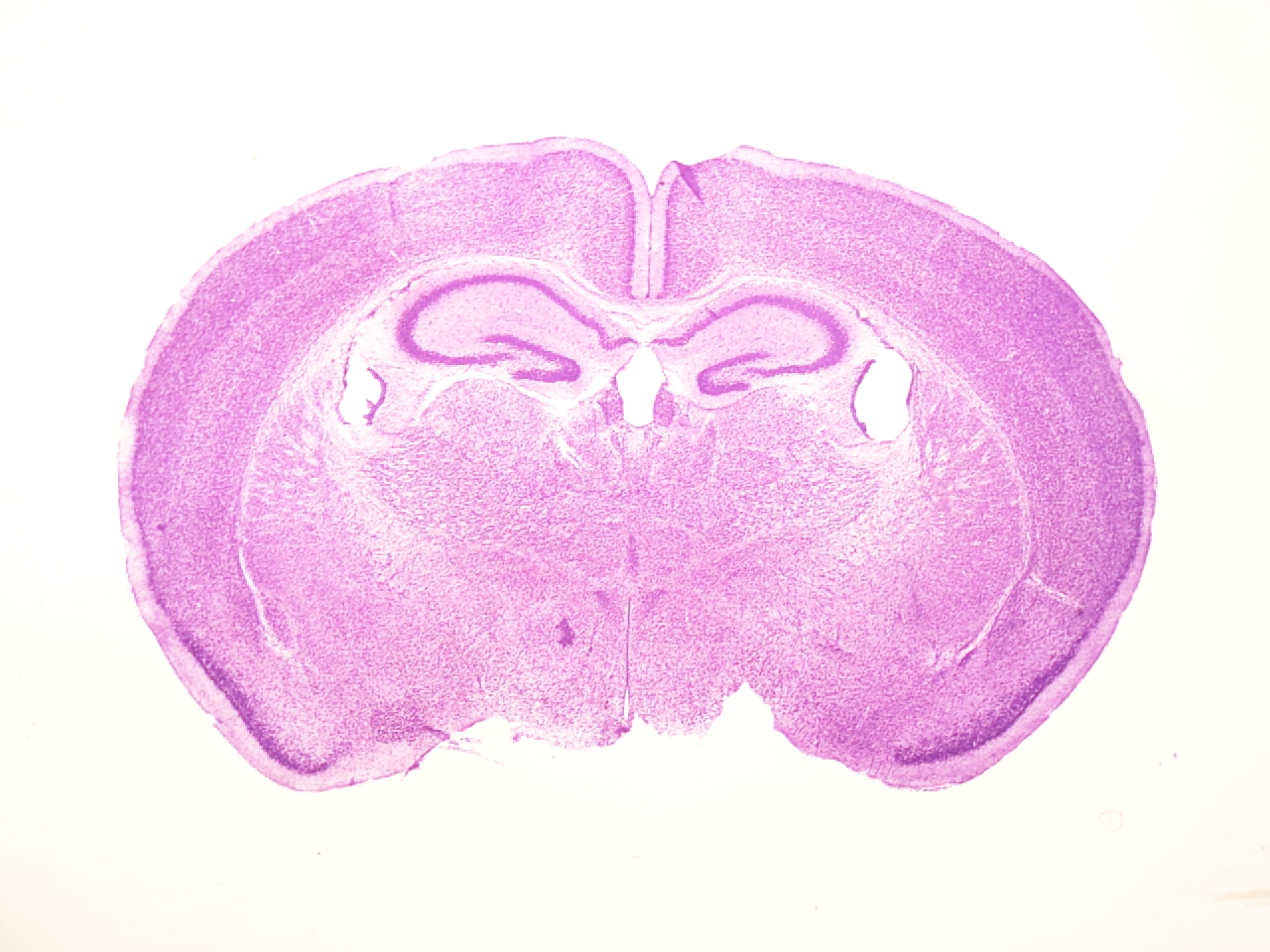

Supplement: Supplementary file 5 — Source data Fig. 4 [file 44321_2024_79_MOESM5_ESM.zip › Figure 4/4A/HI+Ex-4 whole brain.TIF]

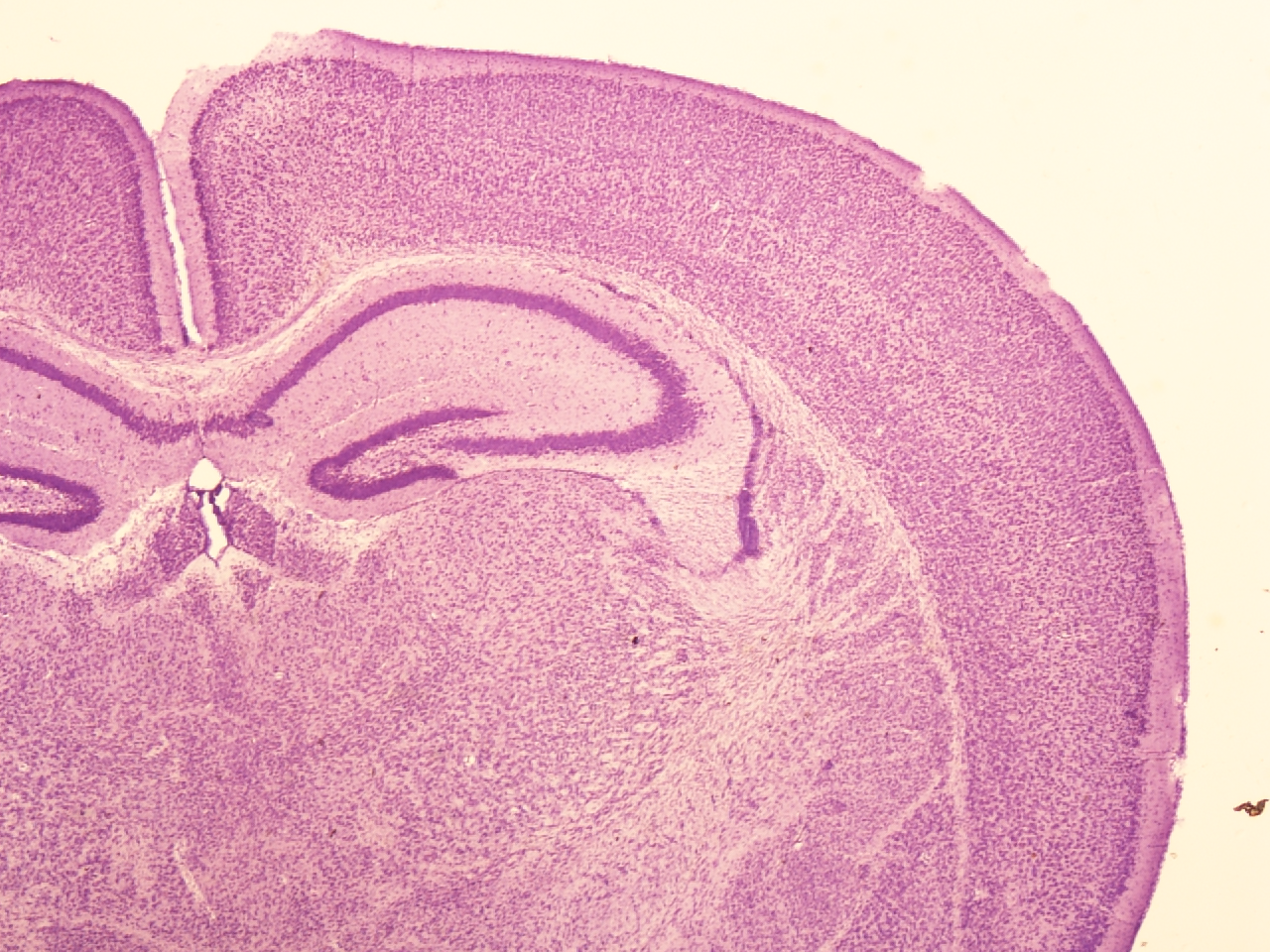

Supplement: Supplementary file 5 — Source data Fig. 4 [file 44321_2024_79_MOESM5_ESM.zip › Figure 4/4A/HI+Sem insert.TIF]

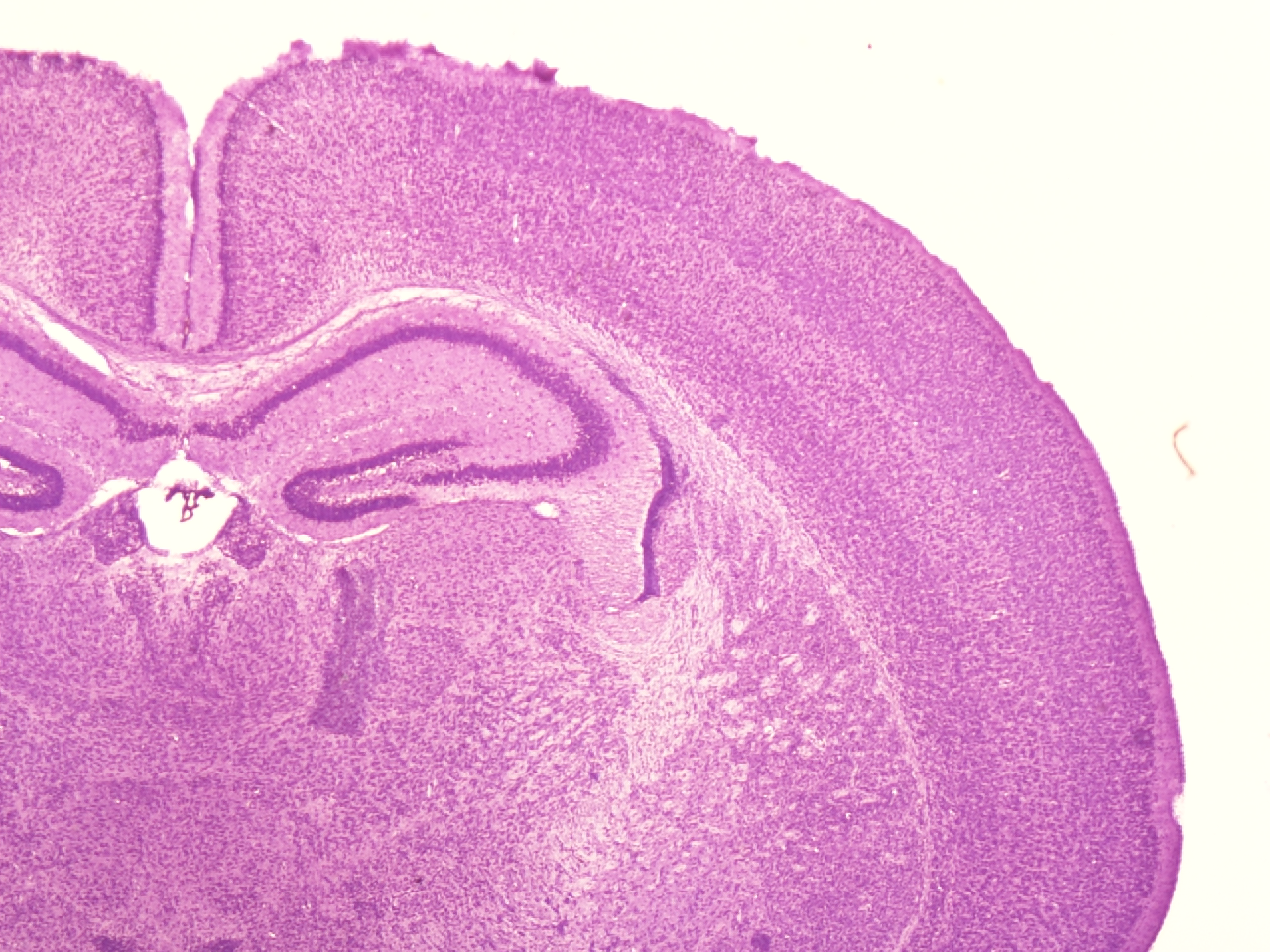

Supplement: Supplementary file 5 — Source data Fig. 4 [file 44321_2024_79_MOESM5_ESM.zip › Figure 4/4A/Saline insert.TIF]

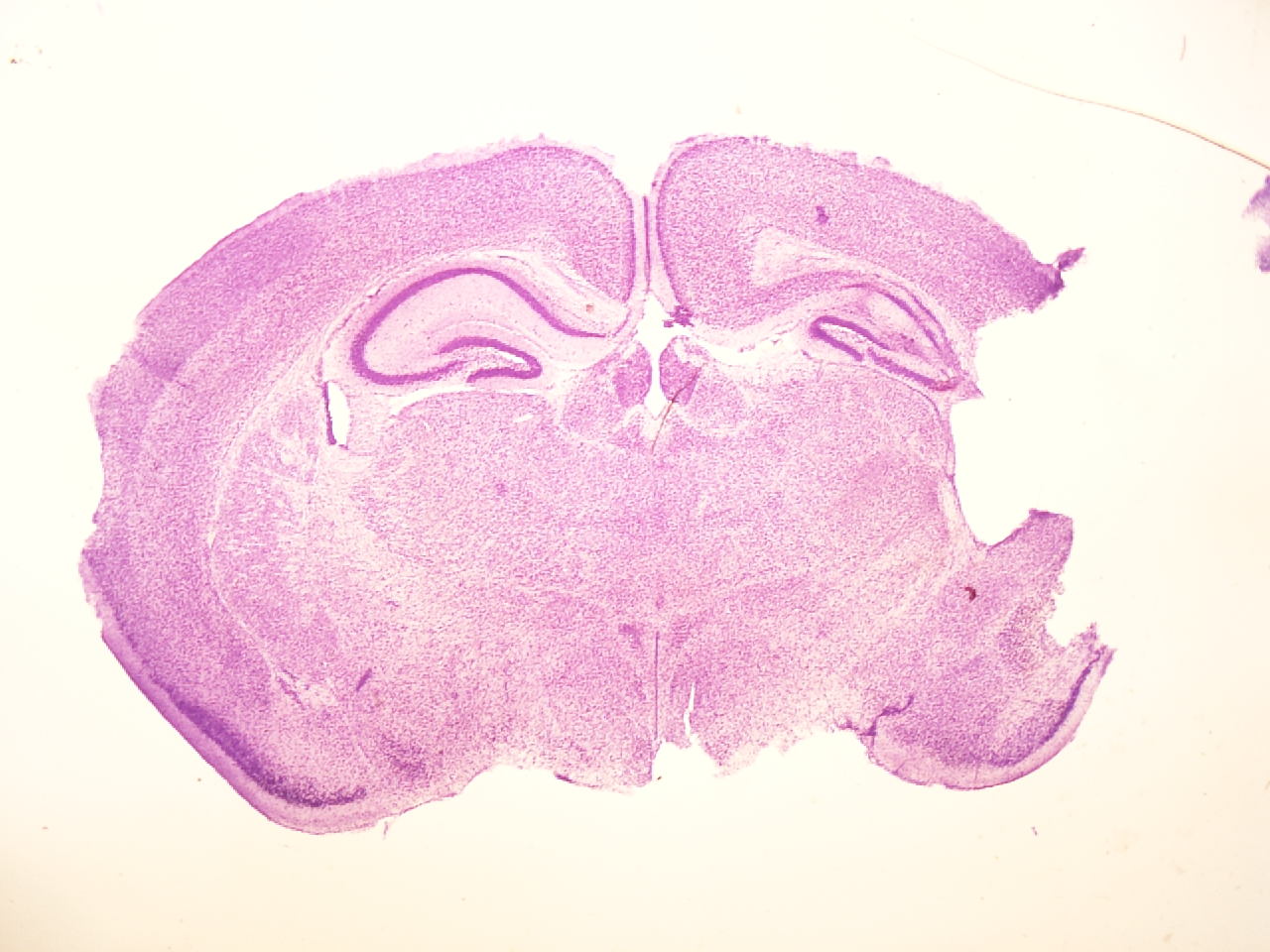

Supplement: Supplementary file 5 — Source data Fig. 4 [file 44321_2024_79_MOESM5_ESM.zip › Figure 4/4A/HI+Sal whole brain.TIF]

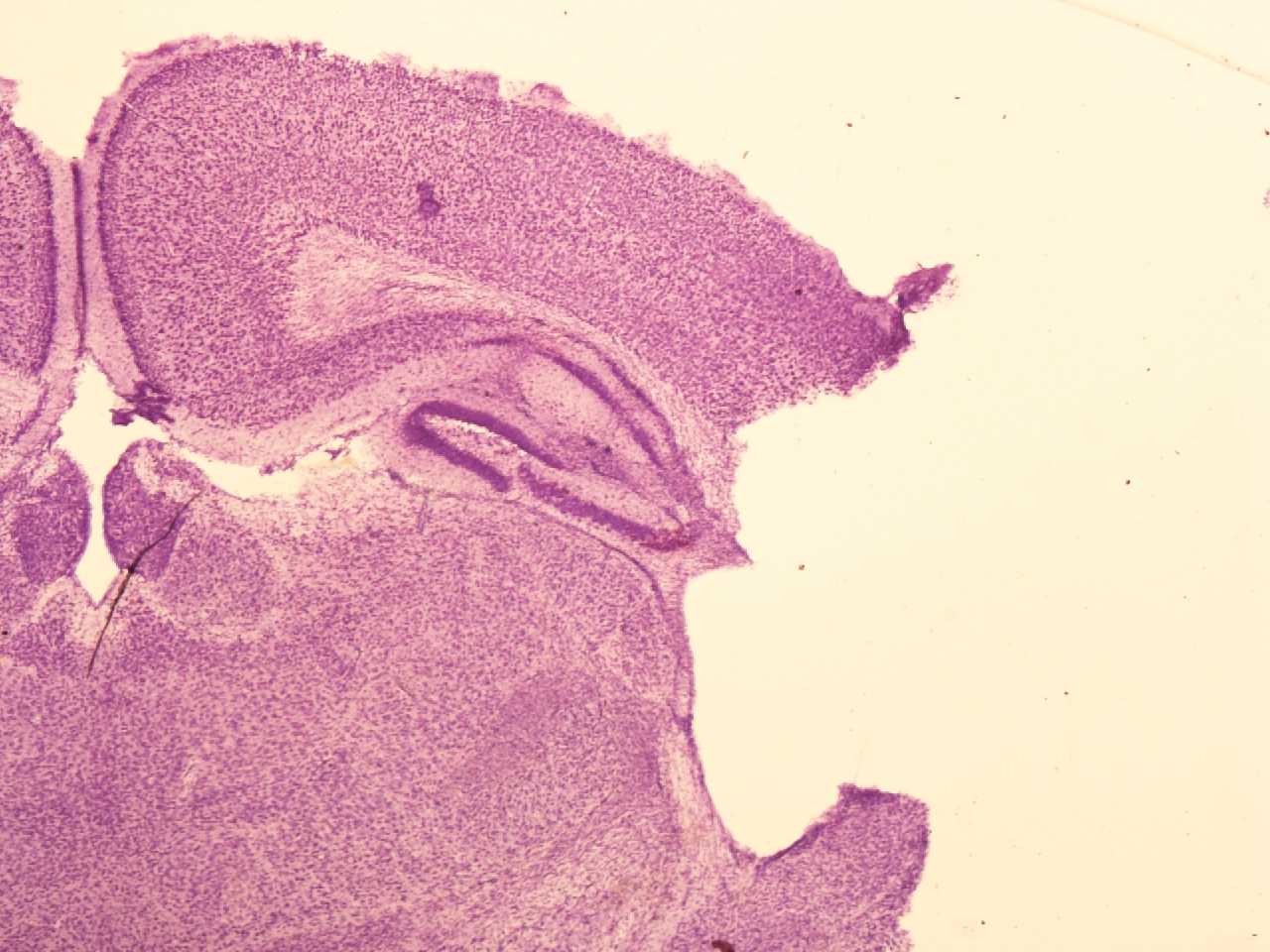

Supplement: Supplementary file 5 — Source data Fig. 4 [file 44321_2024_79_MOESM5_ESM.zip › Figure 4/4A/HI+Sal insert.TIF]

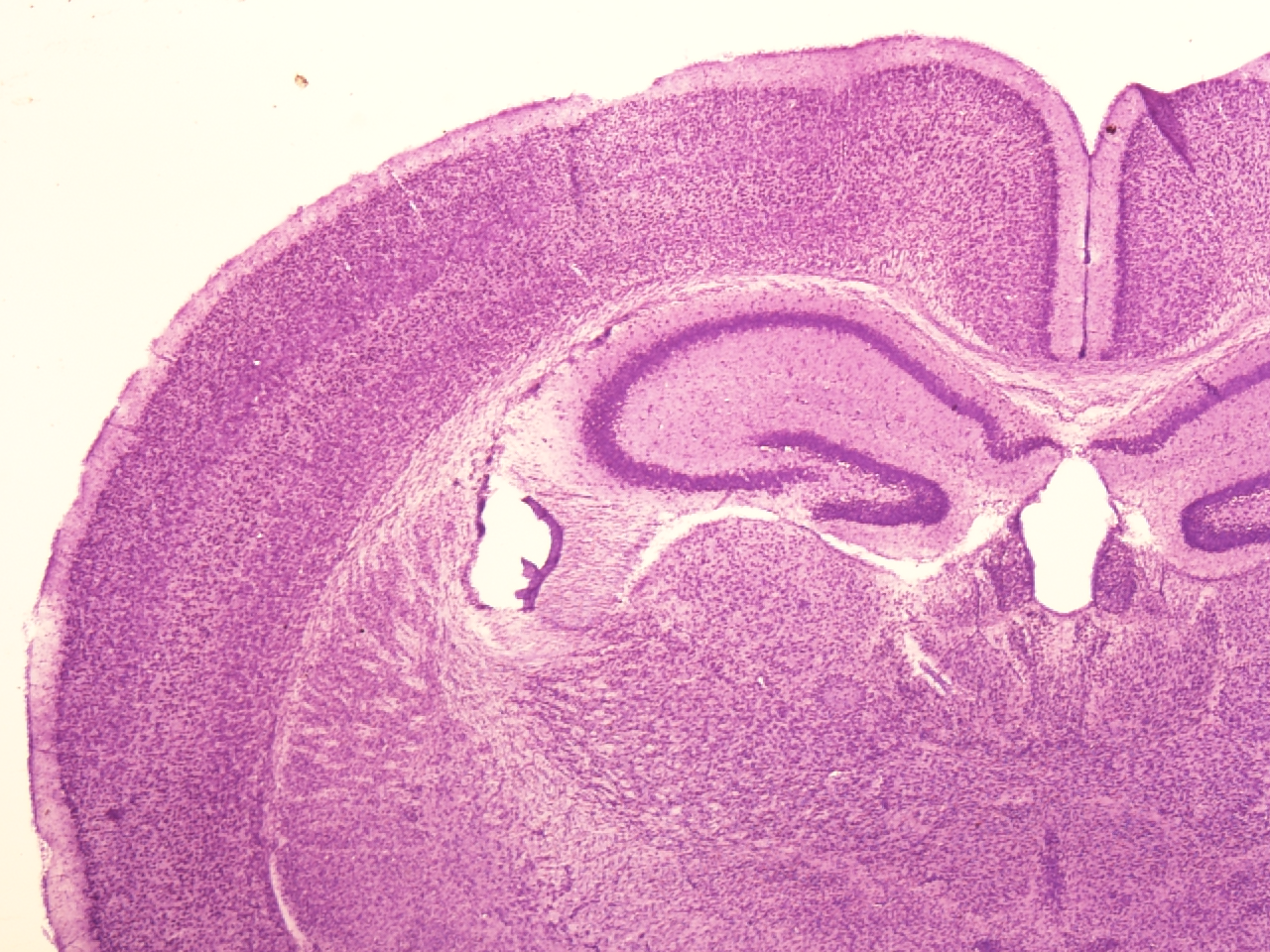

Supplement: Supplementary file 5 — Source data Fig. 4 [file 44321_2024_79_MOESM5_ESM.zip › Figure 4/4A/HI+Ex-4 insert.TIF]

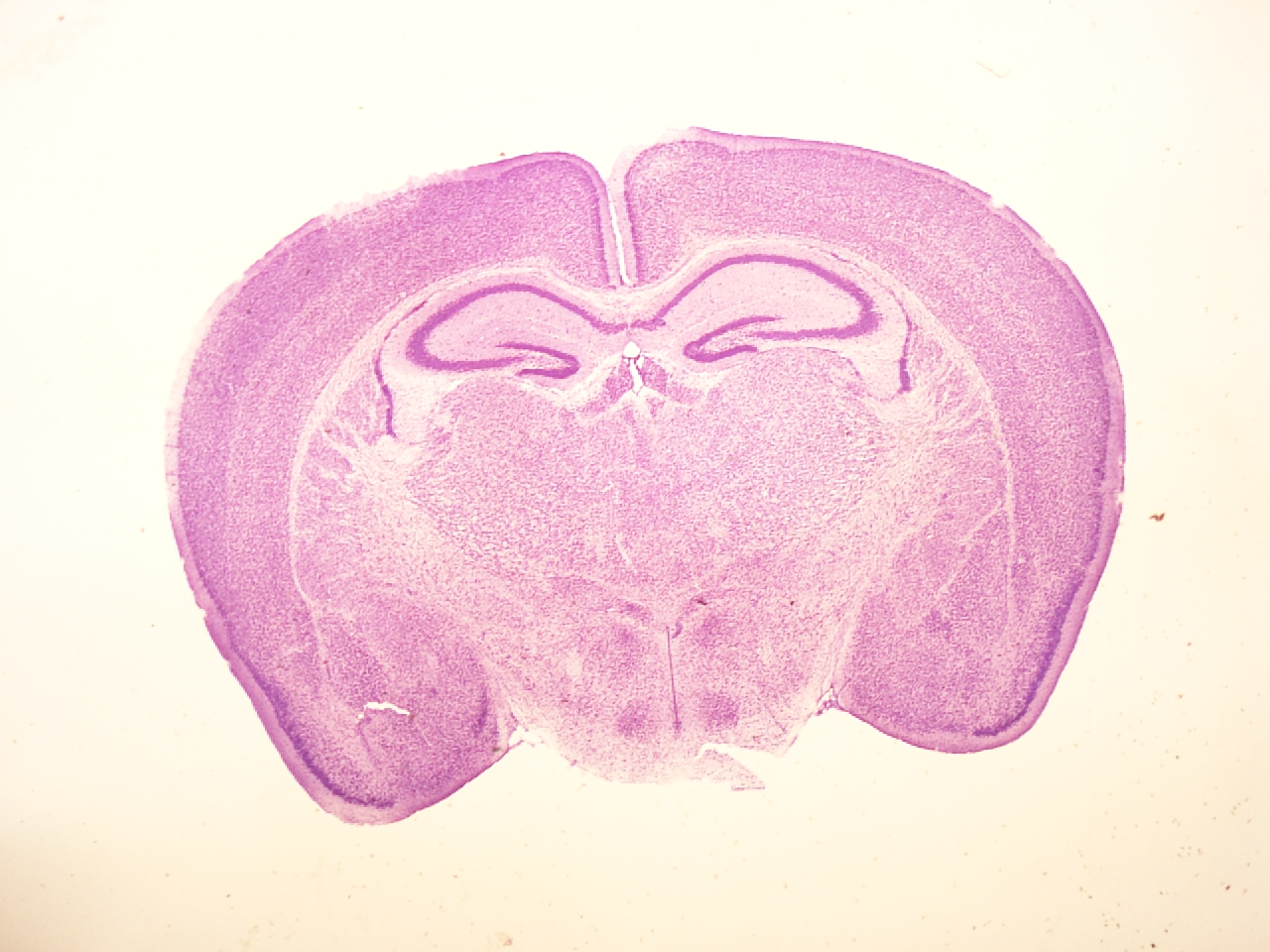

Supplement: Supplementary file 5 — Source data Fig. 4 [file 44321_2024_79_MOESM5_ESM.zip › Figure 4/4A/HI+Sem whole brain.TIF]

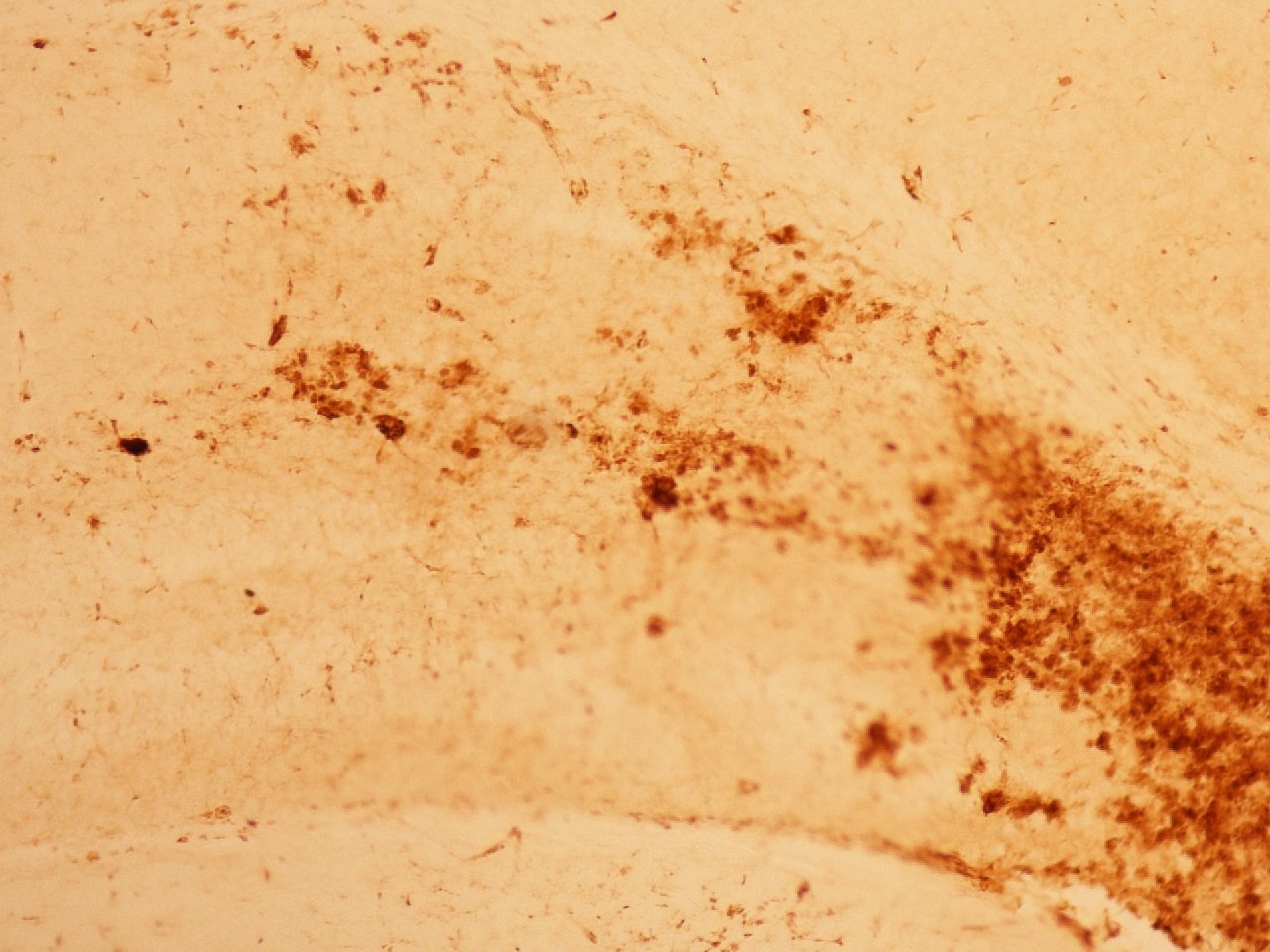

Supplement: Supplementary file 6 — Source data Fig. 5 [file 44321_2024_79_MOESM6_ESM.zip › Figure 5/5A/Hippocampus HI+Sal.TIF]

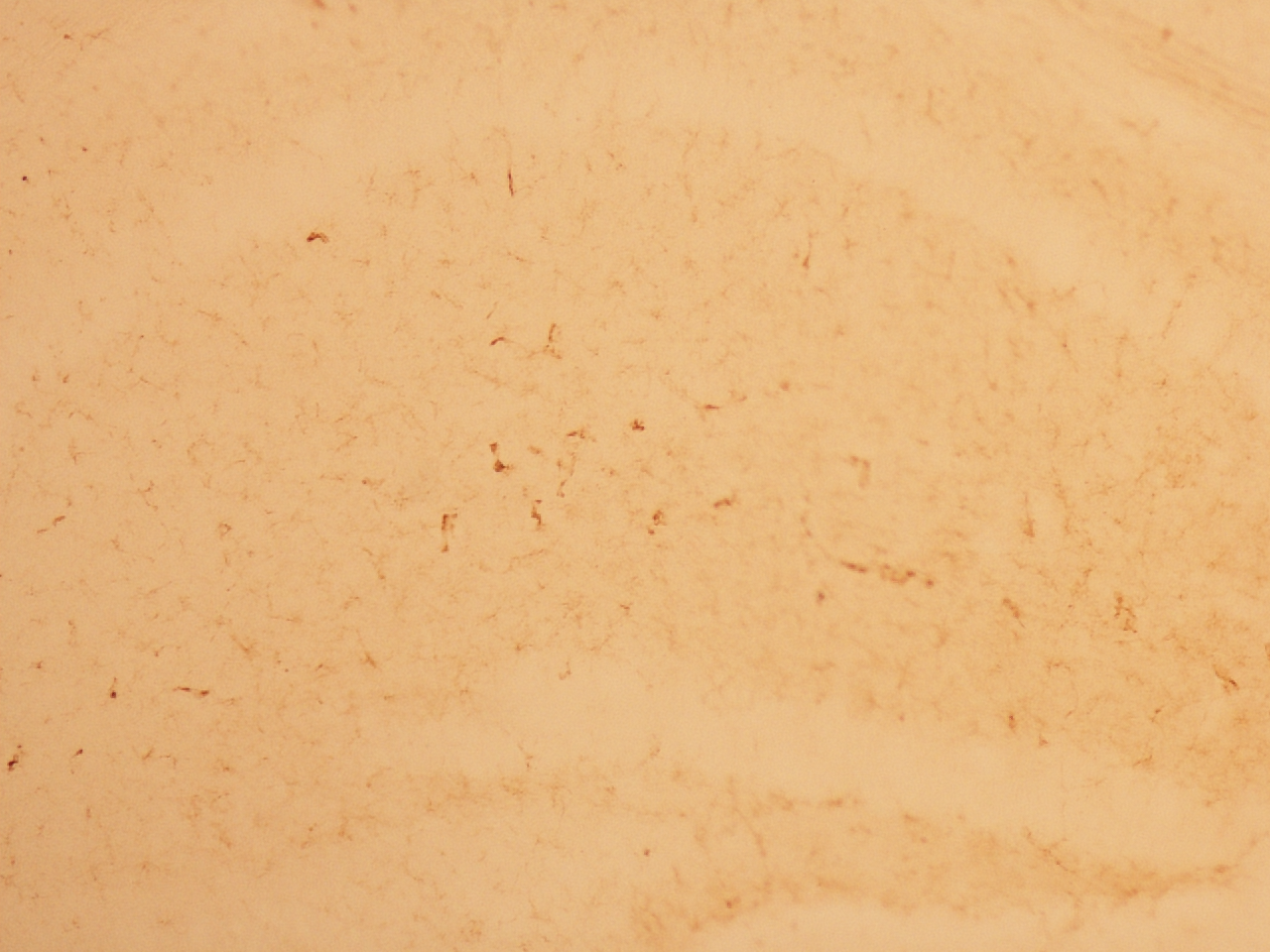

Supplement: Supplementary file 6 — Source data Fig. 5 [file 44321_2024_79_MOESM6_ESM.zip › Figure 5/5A/Hippocampus HI+Ex-4.TIF]

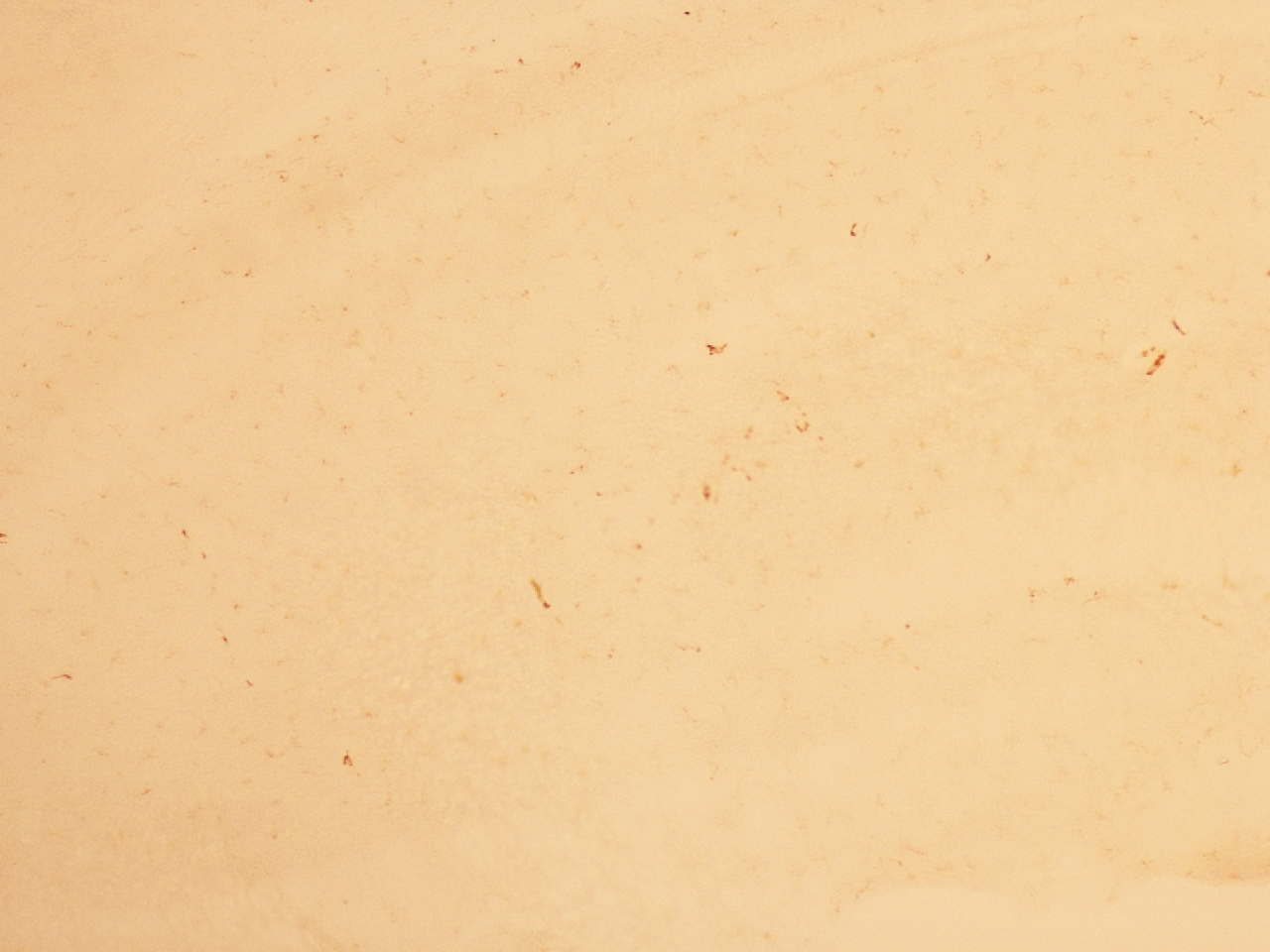

Supplement: Supplementary file 6 — Source data Fig. 5 [file 44321_2024_79_MOESM6_ESM.zip › Figure 5/5A/Hippocampus Saline.TIF]

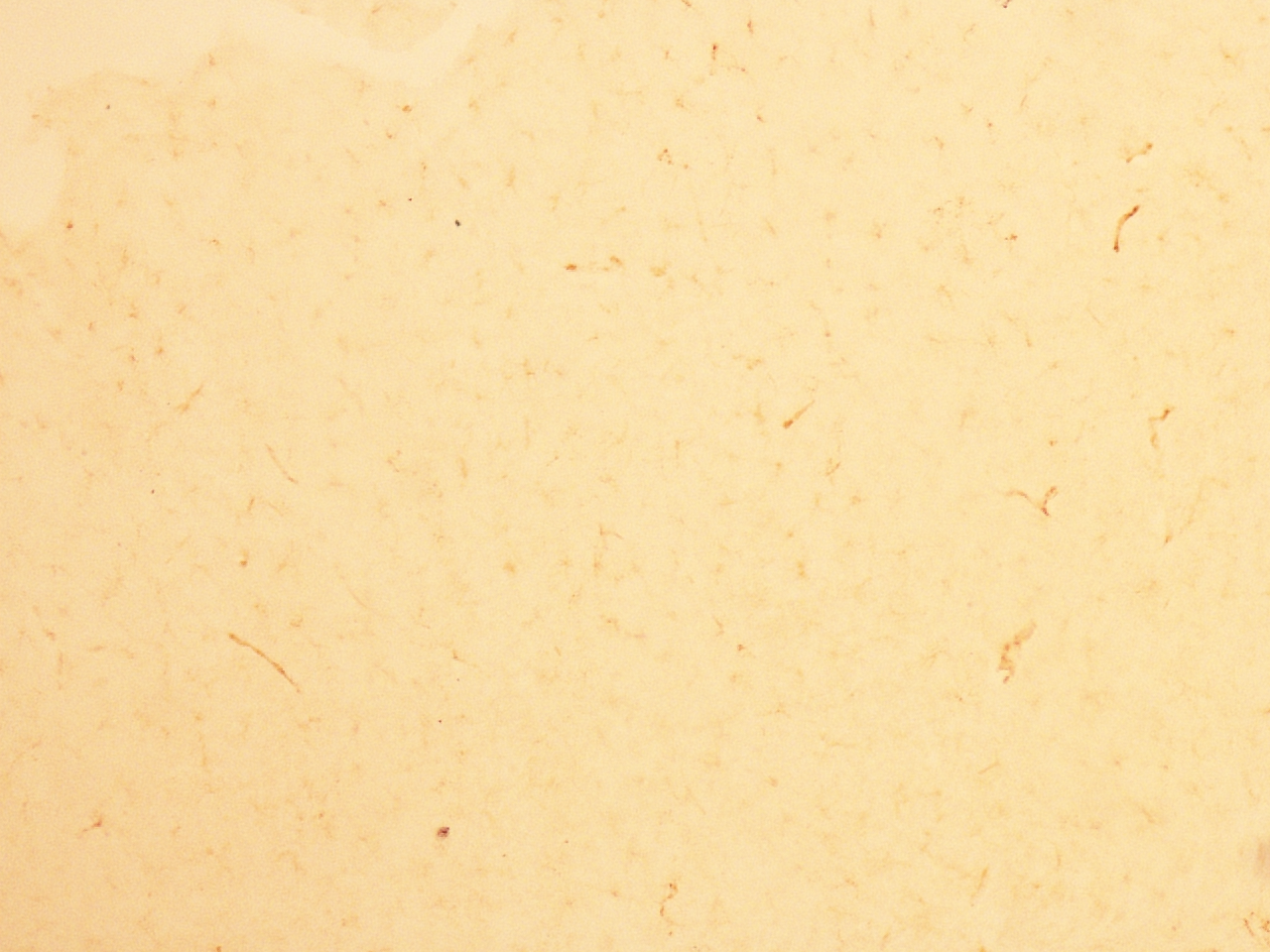

Supplement: Supplementary file 6 — Source data Fig. 5 [file 44321_2024_79_MOESM6_ESM.zip › Figure 5/5A/Cortex HI+Sem.TIF]

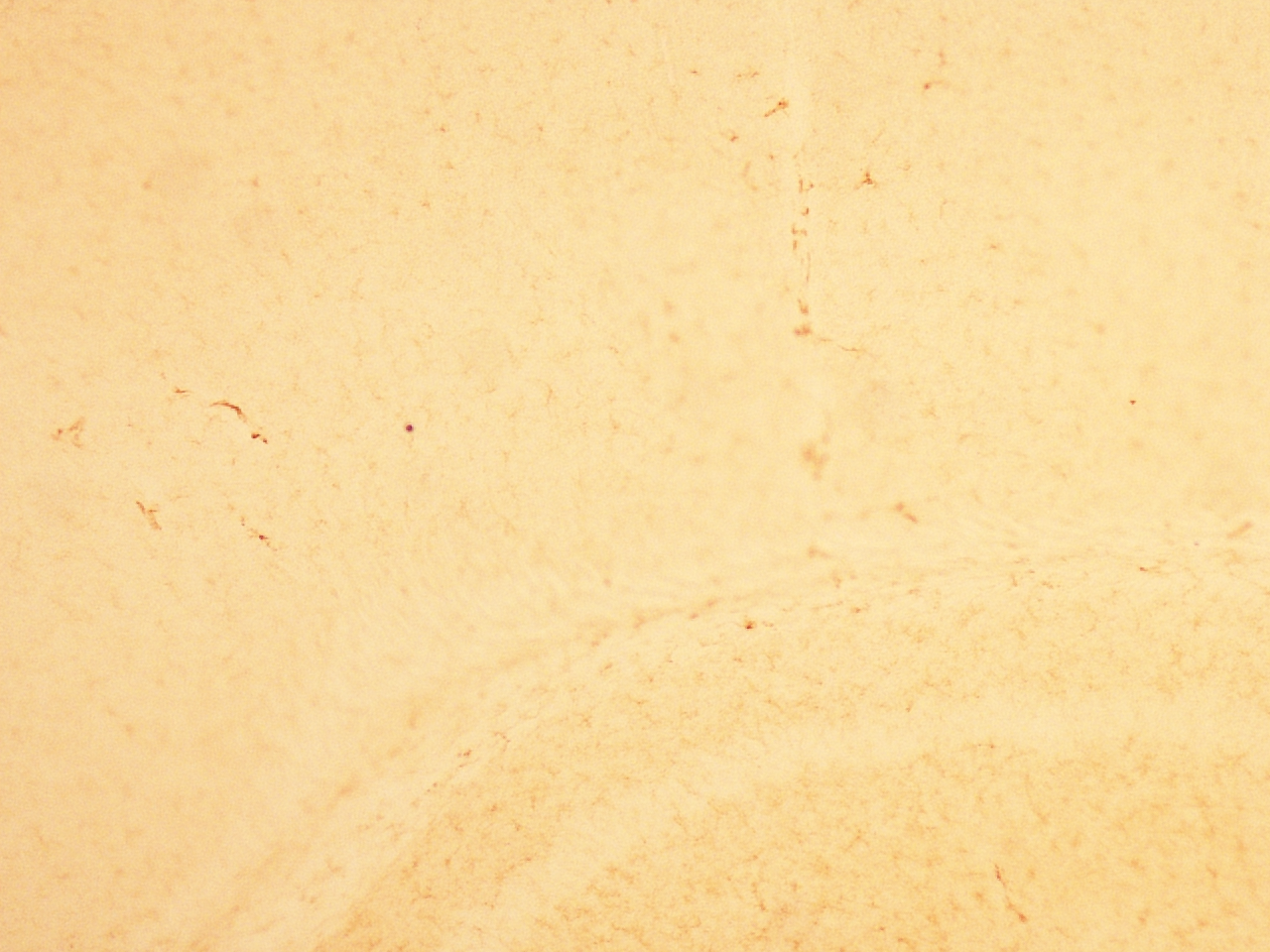

Supplement: Supplementary file 6 — Source data Fig. 5 [file 44321_2024_79_MOESM6_ESM.zip › Figure 5/5A/Hippocampus HI+Sem.TIF]

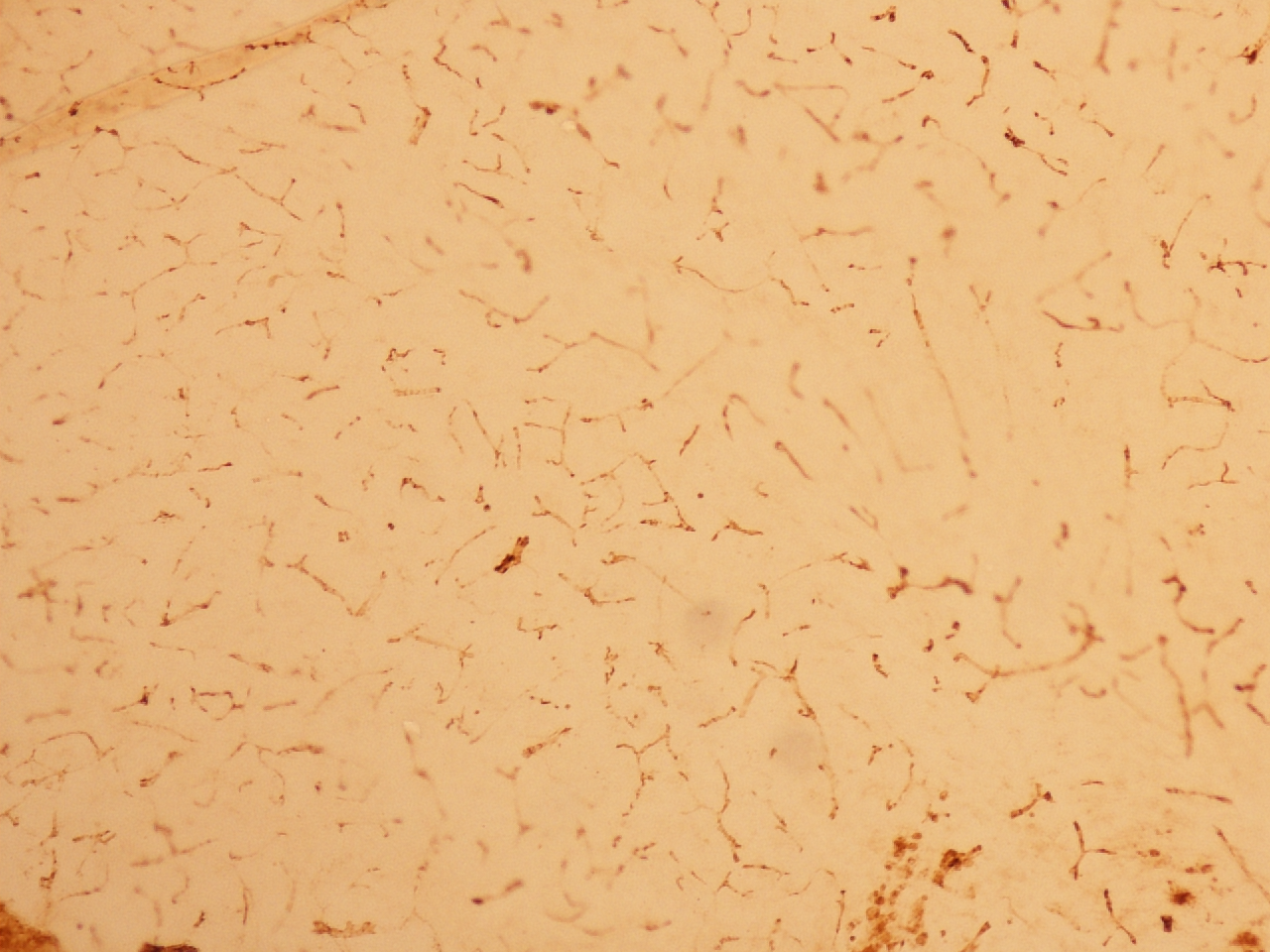

Supplement: Supplementary file 6 — Source data Fig. 5 [file 44321_2024_79_MOESM6_ESM.zip › Figure 5/5A/Cortex HI+Sal.TIF]

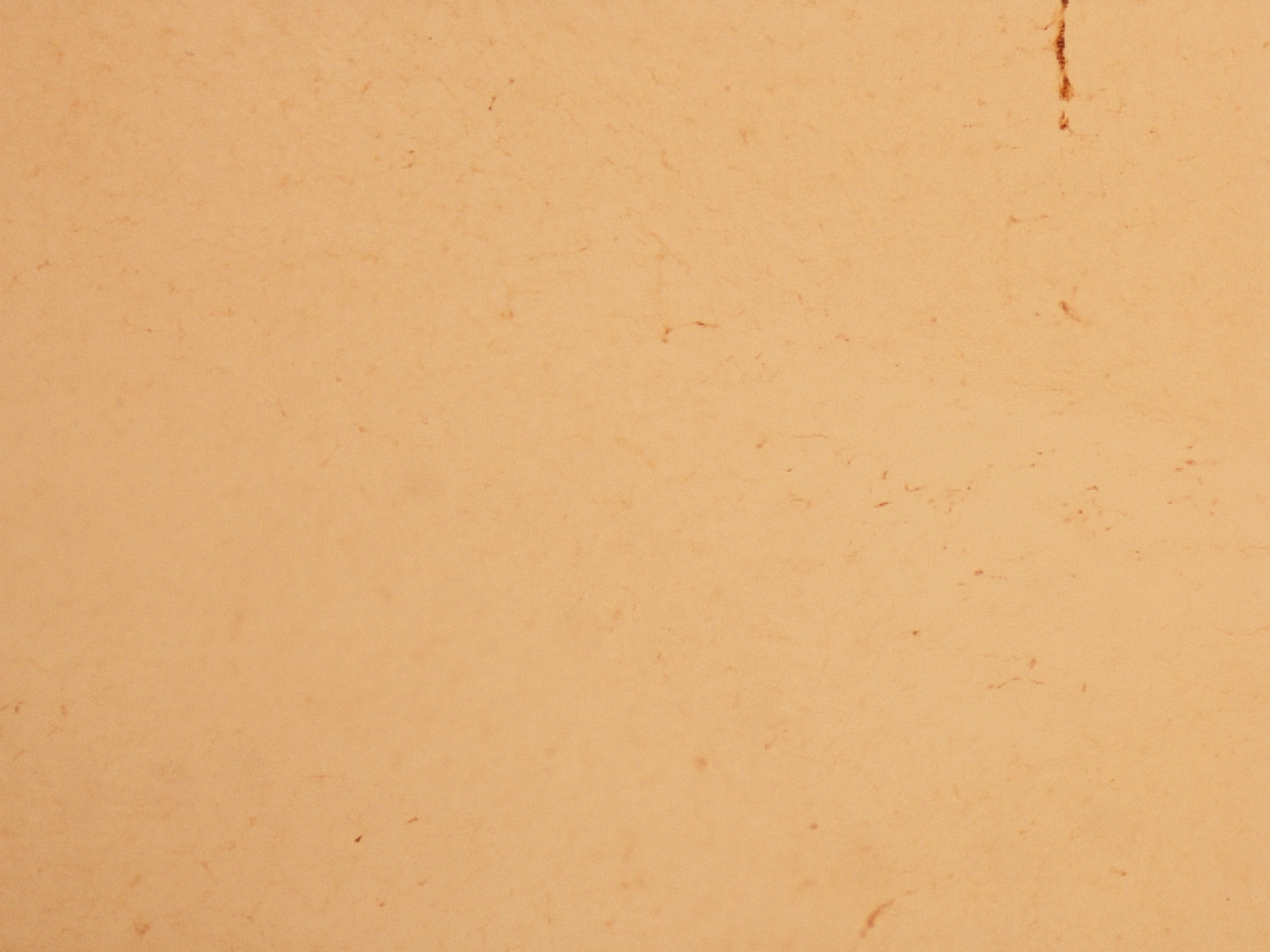

Supplement: Supplementary file 6 — Source data Fig. 5 [file 44321_2024_79_MOESM6_ESM.zip › Figure 5/5A/Cortex HI+Ex-4.TIF]

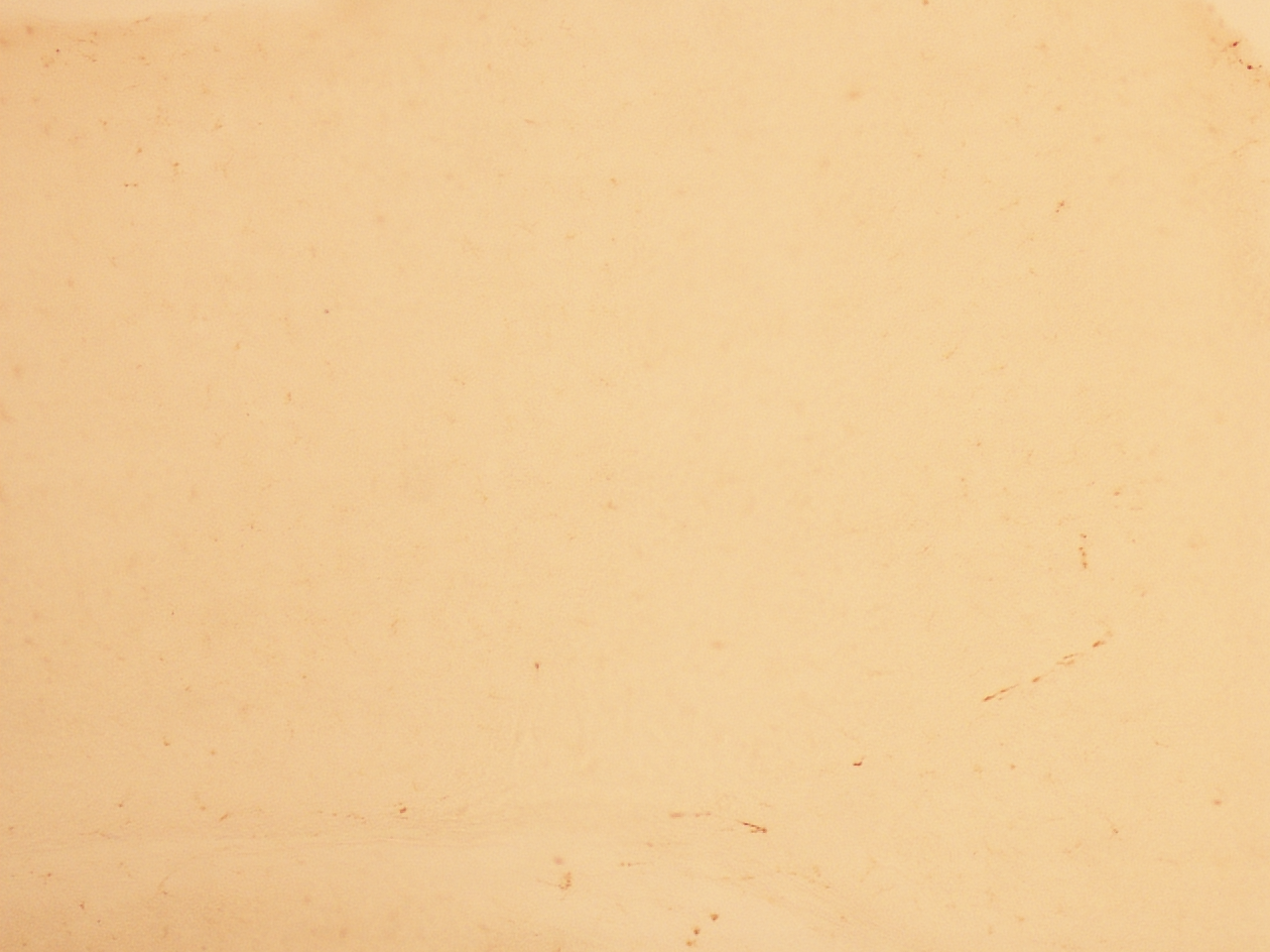

Supplement: Supplementary file 6 — Source data Fig. 5 [file 44321_2024_79_MOESM6_ESM.zip › Figure 5/5A/Cortex Saline.TIF]

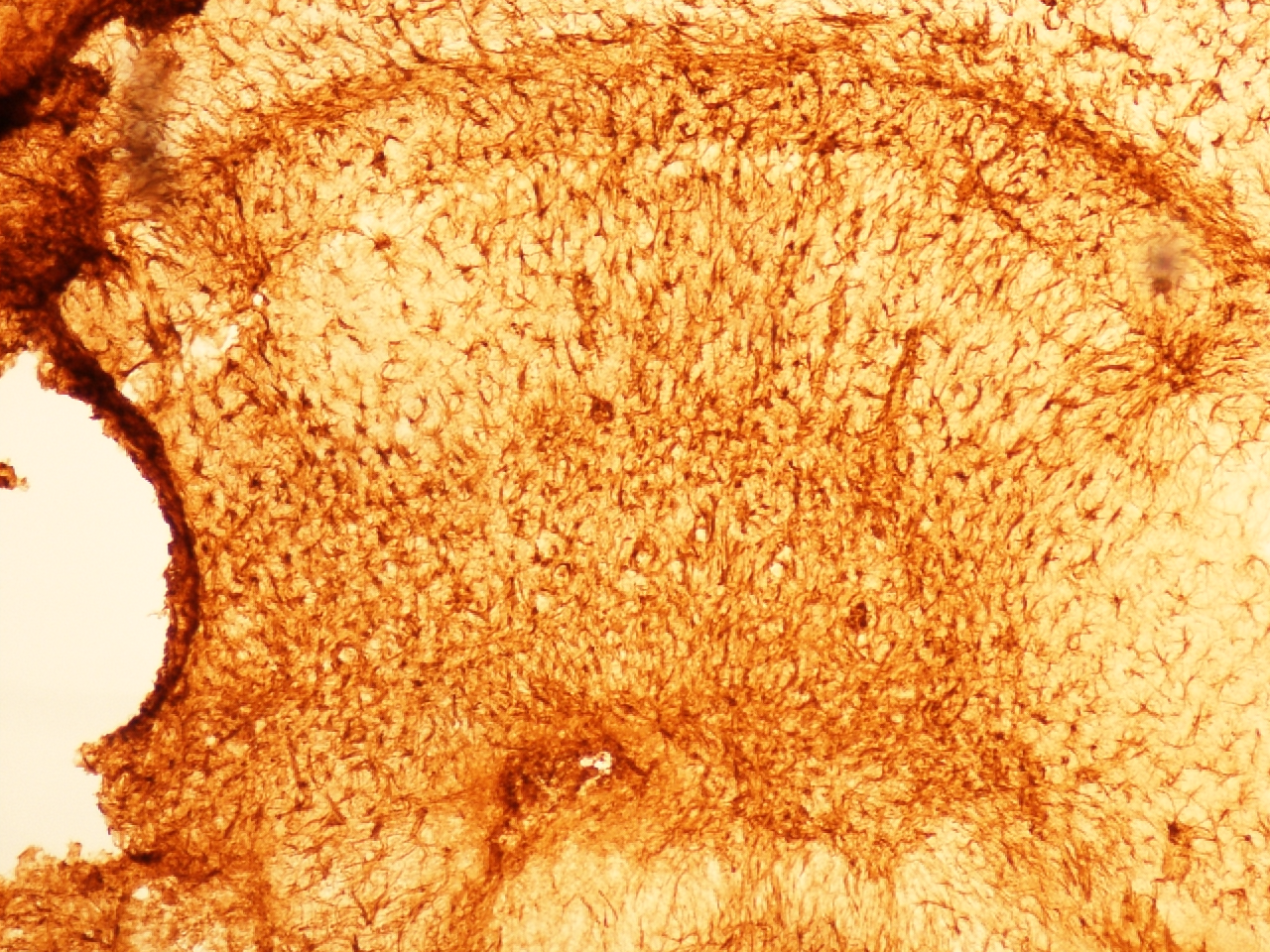

Supplement: Supplementary file 6 — Source data Fig. 5 [file 44321_2024_79_MOESM6_ESM.zip › Figure 5/5C/Hippocampus HI+Sal.TIF]

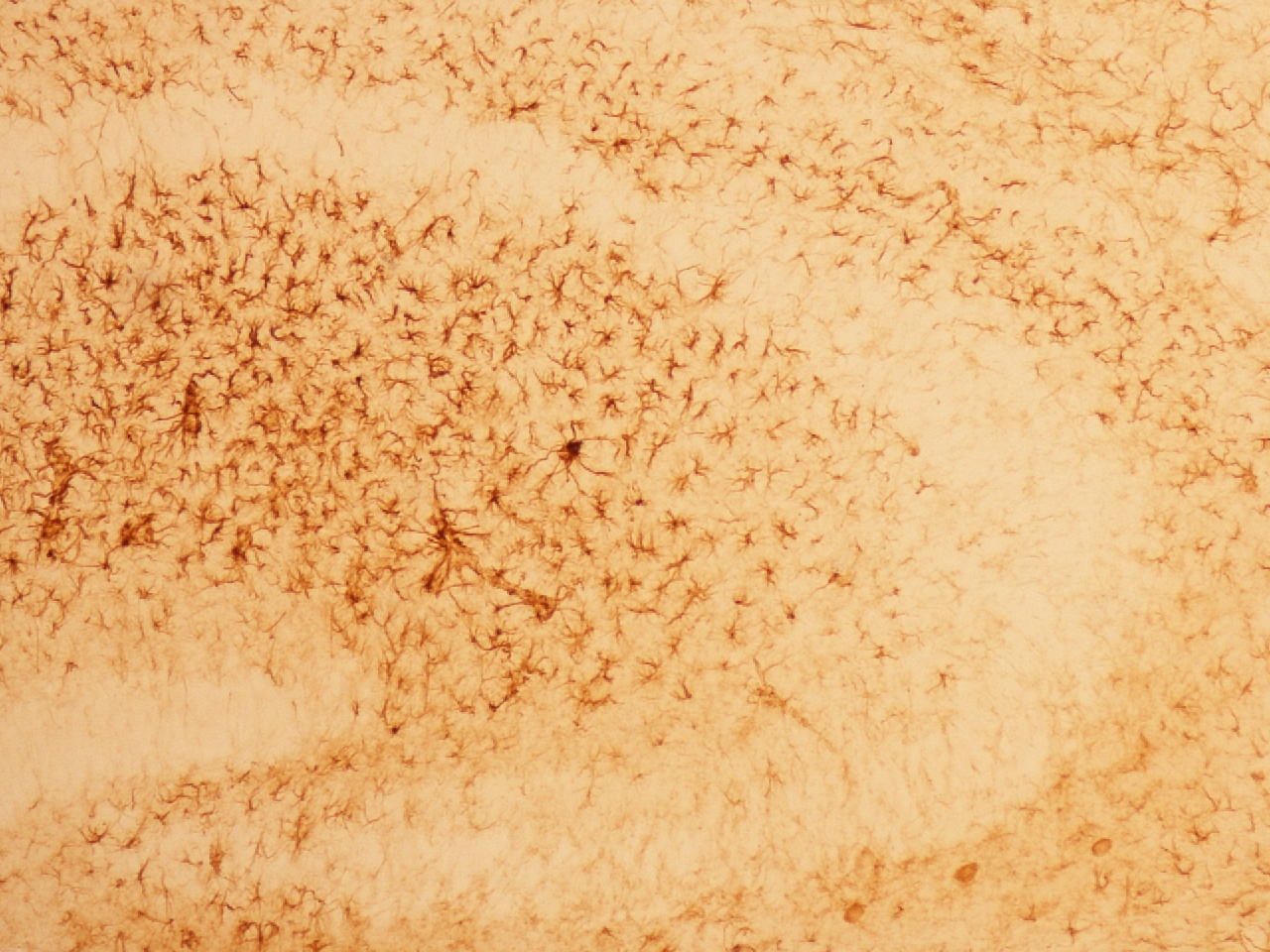

Supplement: Supplementary file 6 — Source data Fig. 5 [file 44321_2024_79_MOESM6_ESM.zip › Figure 5/5C/Hippocampus HI+Ex-4.TIF]

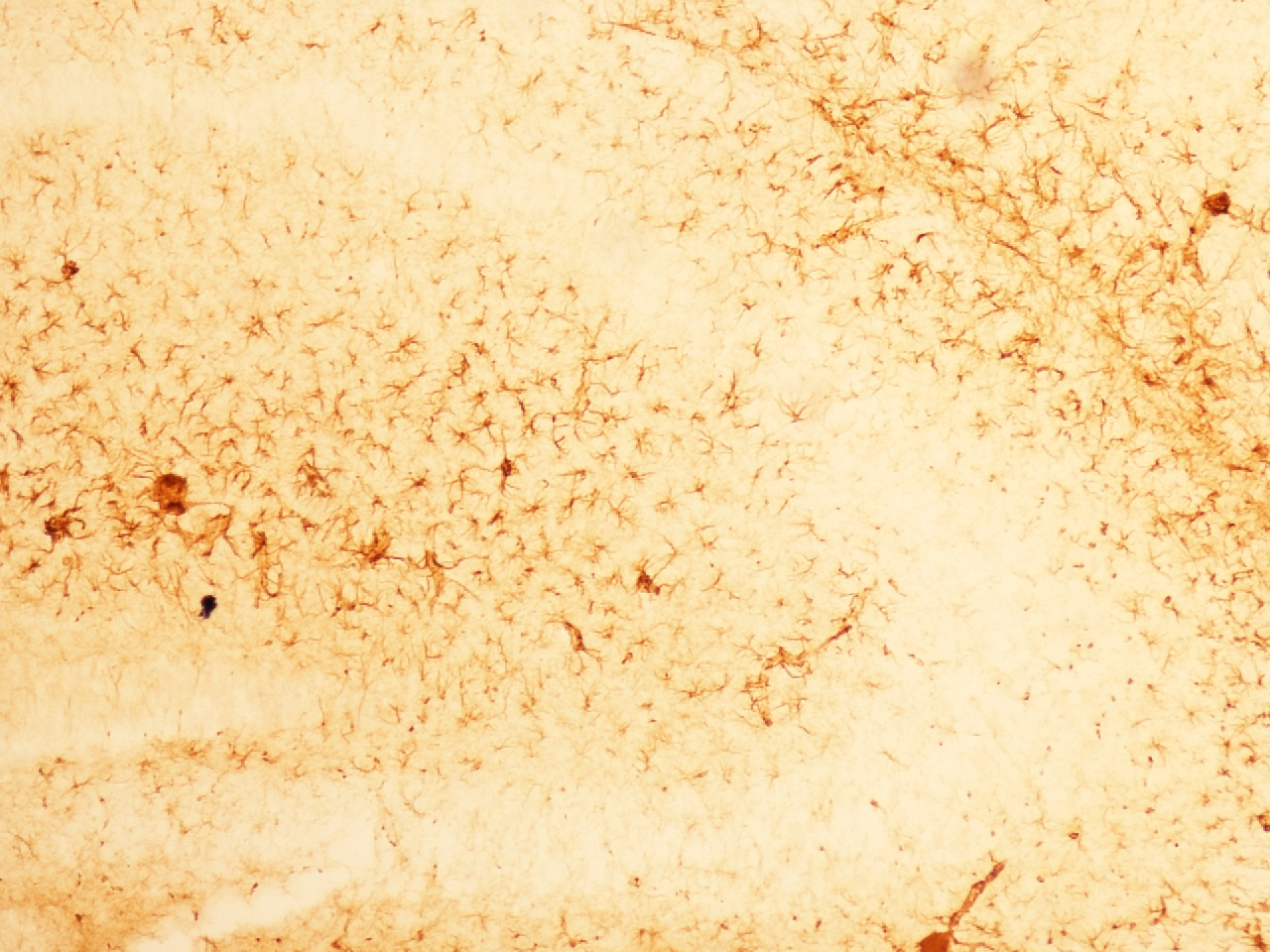

Supplement: Supplementary file 6 — Source data Fig. 5 [file 44321_2024_79_MOESM6_ESM.zip › Figure 5/5C/Hippocampus Saline.TIF]

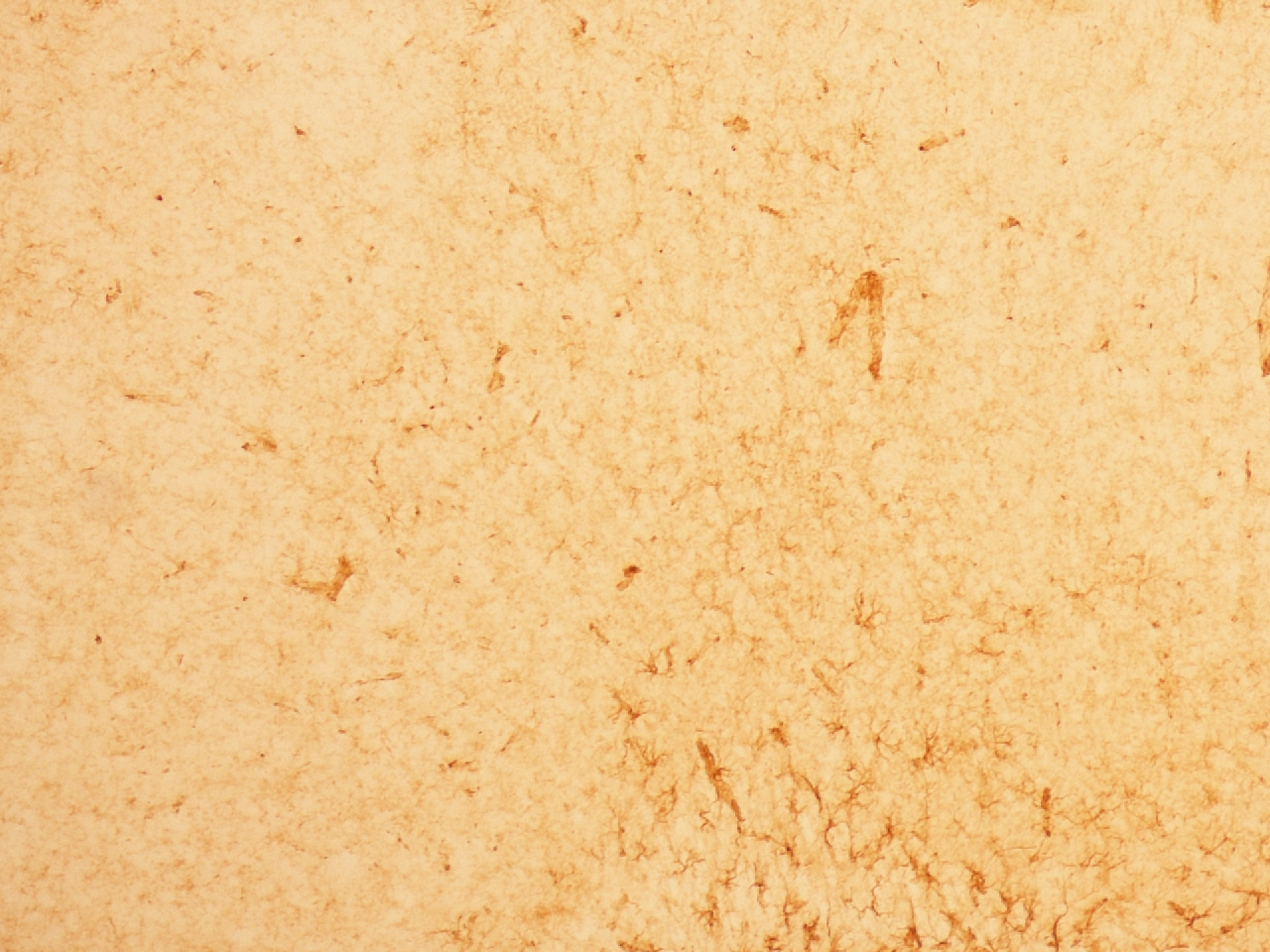

Supplement: Supplementary file 6 — Source data Fig. 5 [file 44321_2024_79_MOESM6_ESM.zip › Figure 5/5C/Cortex HI+Sem.TIF]

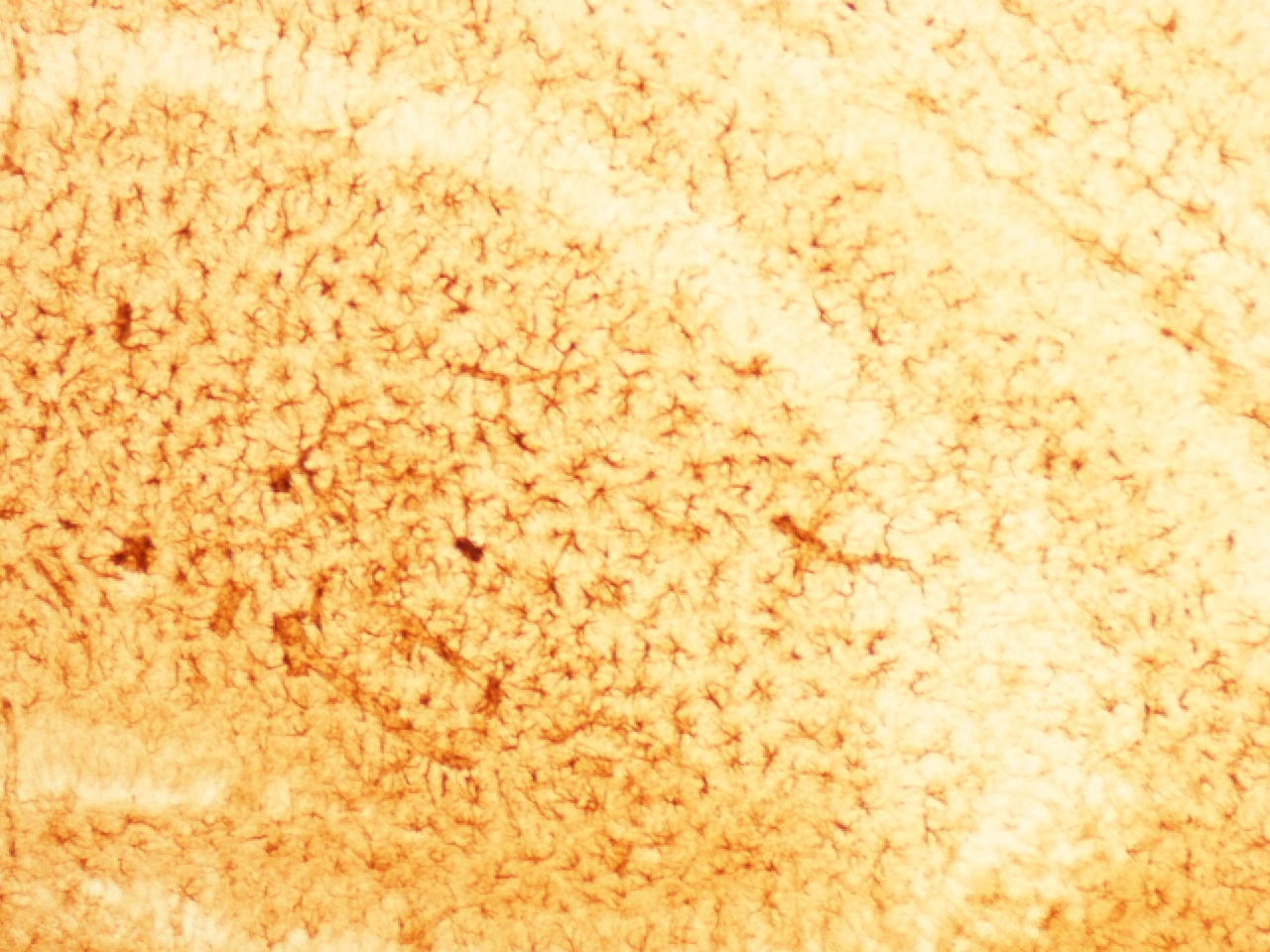

Supplement: Supplementary file 6 — Source data Fig. 5 [file 44321_2024_79_MOESM6_ESM.zip › Figure 5/5C/Hippocampus HI+Sem.TIF]

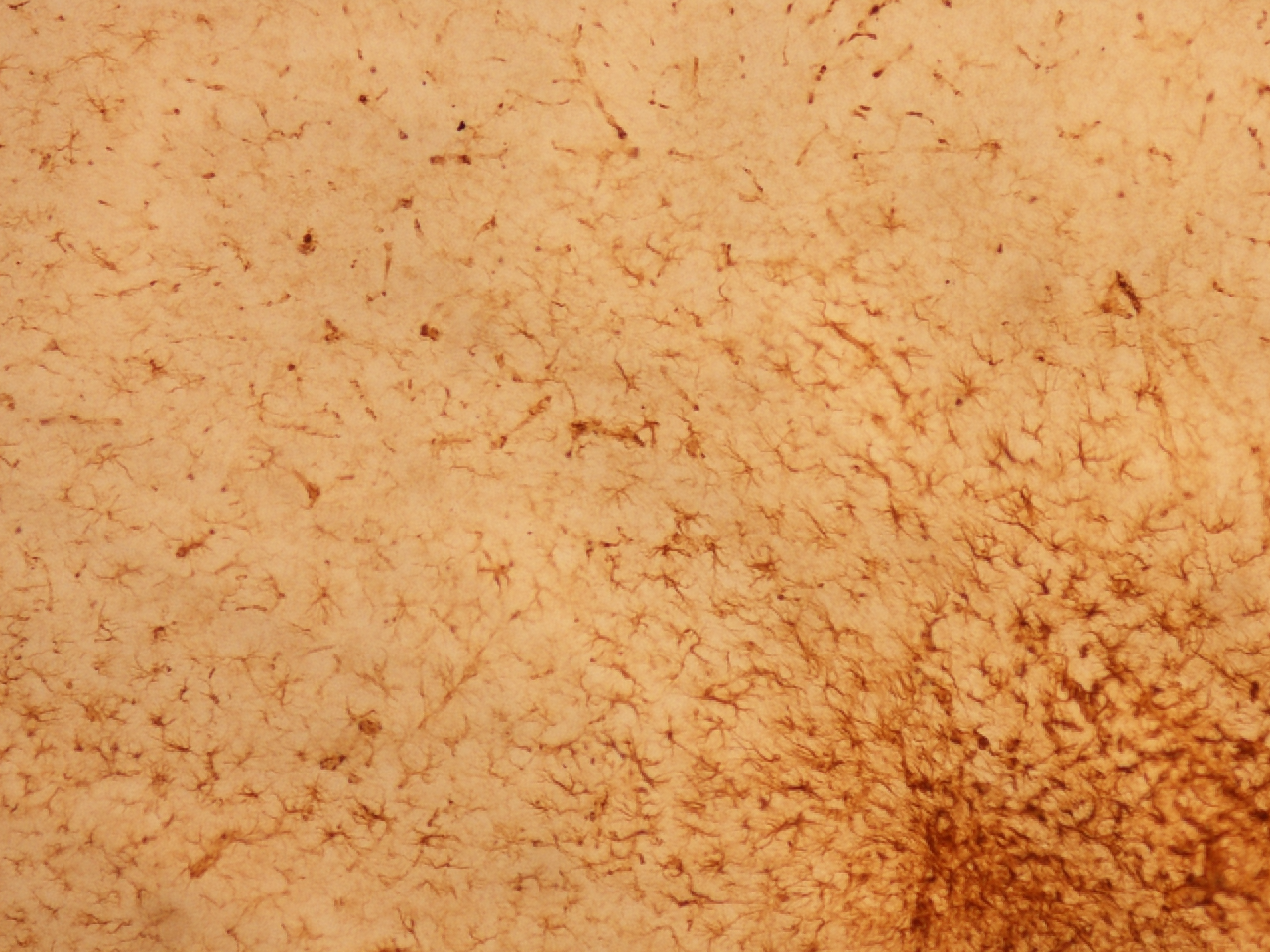

Supplement: Supplementary file 6 — Source data Fig. 5 [file 44321_2024_79_MOESM6_ESM.zip › Figure 5/5C/Cortex HI+Sal.TIF]

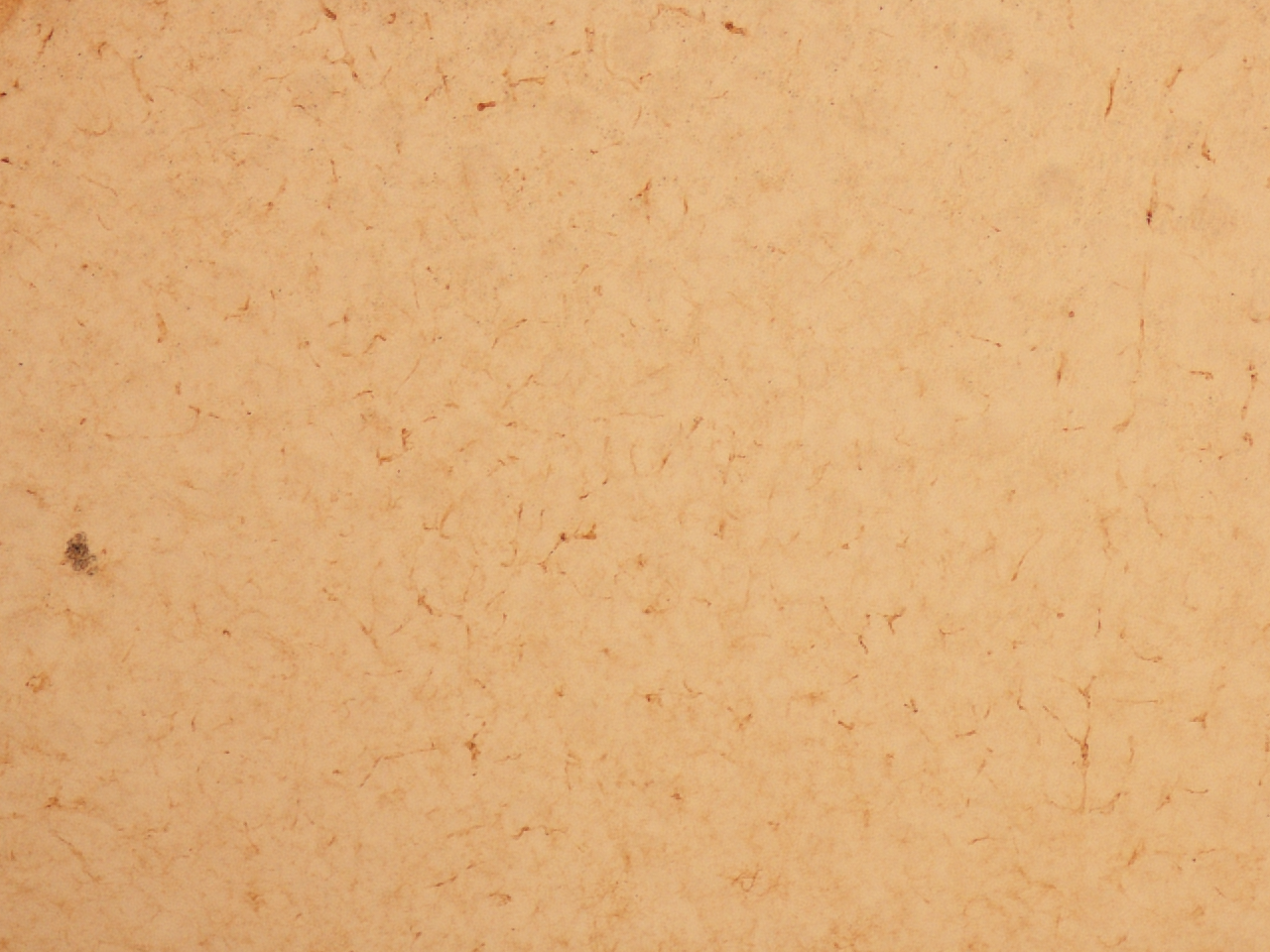

Supplement: Supplementary file 6 — Source data Fig. 5 [file 44321_2024_79_MOESM6_ESM.zip › Figure 5/5C/Cortex HI+Ex-4.TIF]

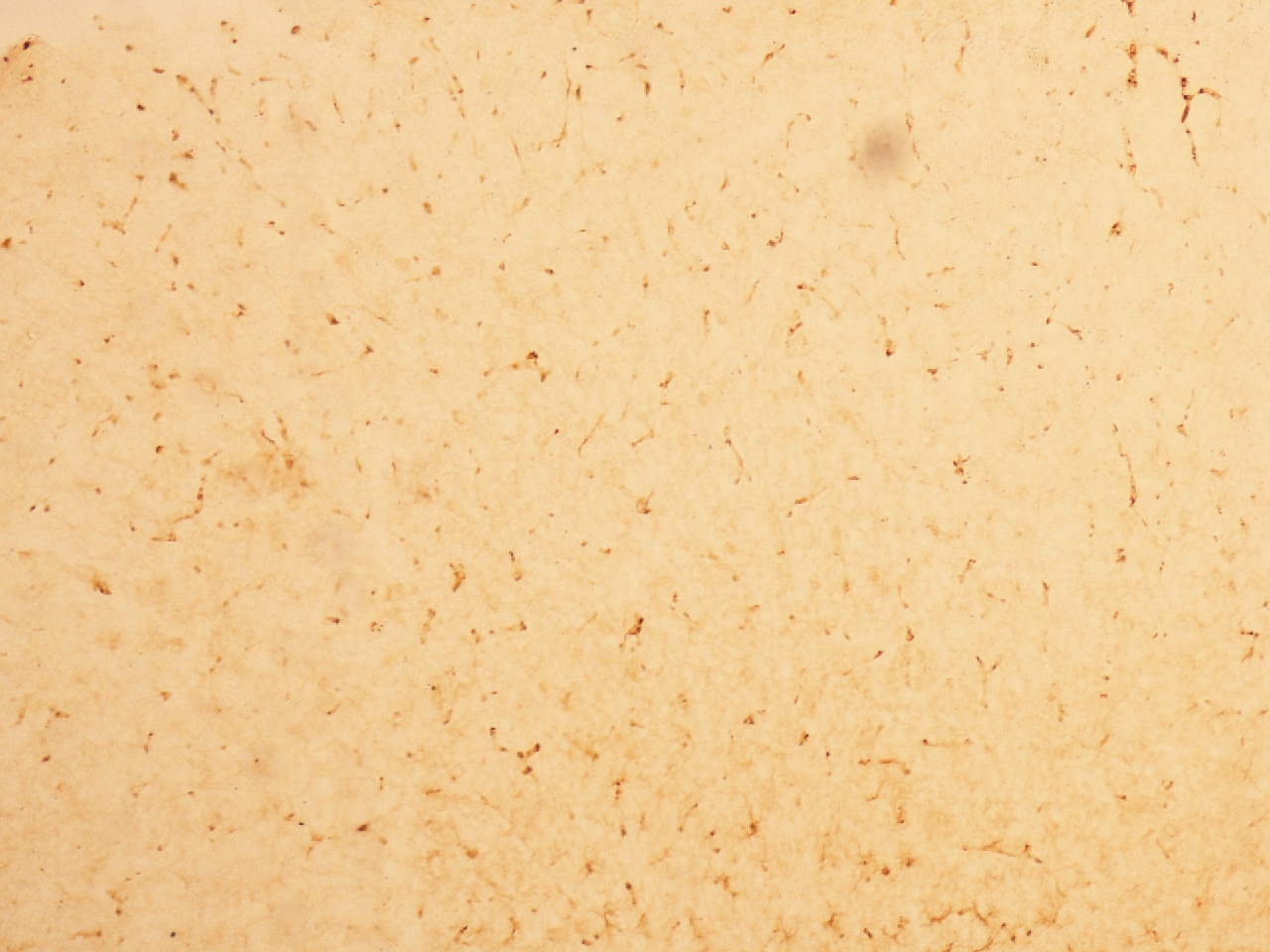

Supplement: Supplementary file 6 — Source data Fig. 5 [file 44321_2024_79_MOESM6_ESM.zip › Figure 5/5C/Cortex Saline.TIF]

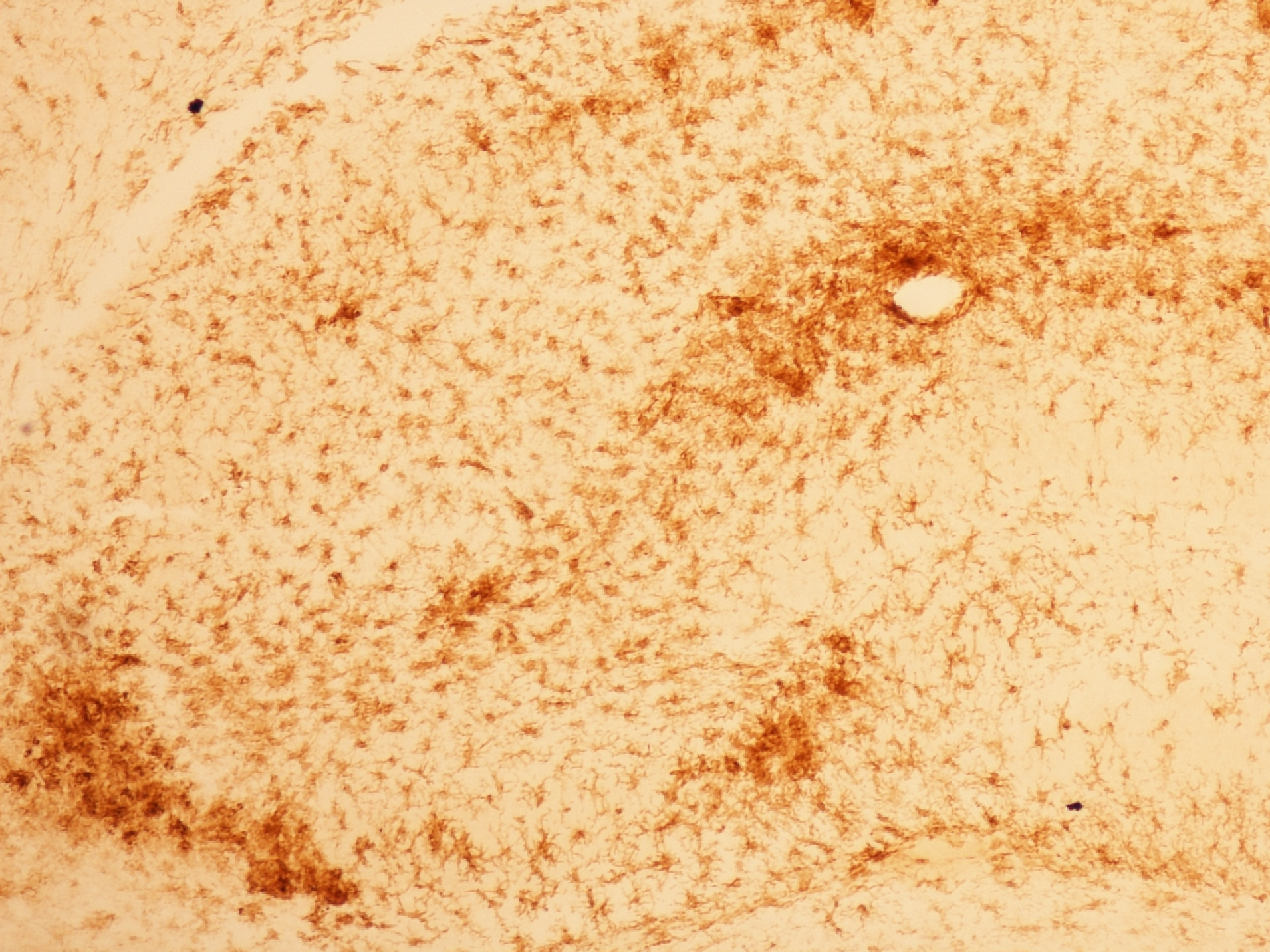

Supplement: Supplementary file 6 — Source data Fig. 5 [file 44321_2024_79_MOESM6_ESM.zip › Figure 5/5B/Hippocampus HI+Sal.TIF]

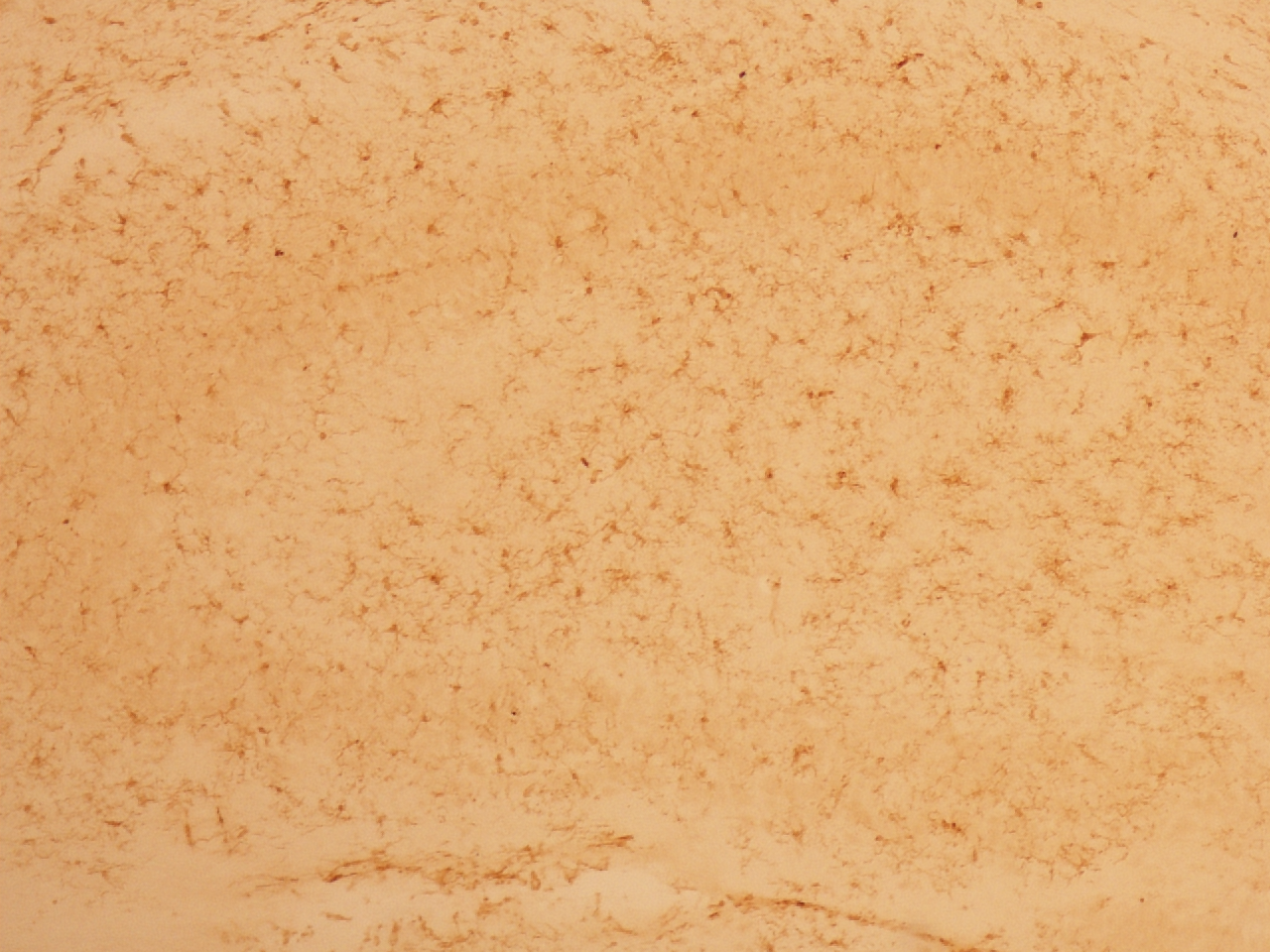

Supplement: Supplementary file 6 — Source data Fig. 5 [file 44321_2024_79_MOESM6_ESM.zip › Figure 5/5B/Hippocampus HI+Ex-4.TIF]

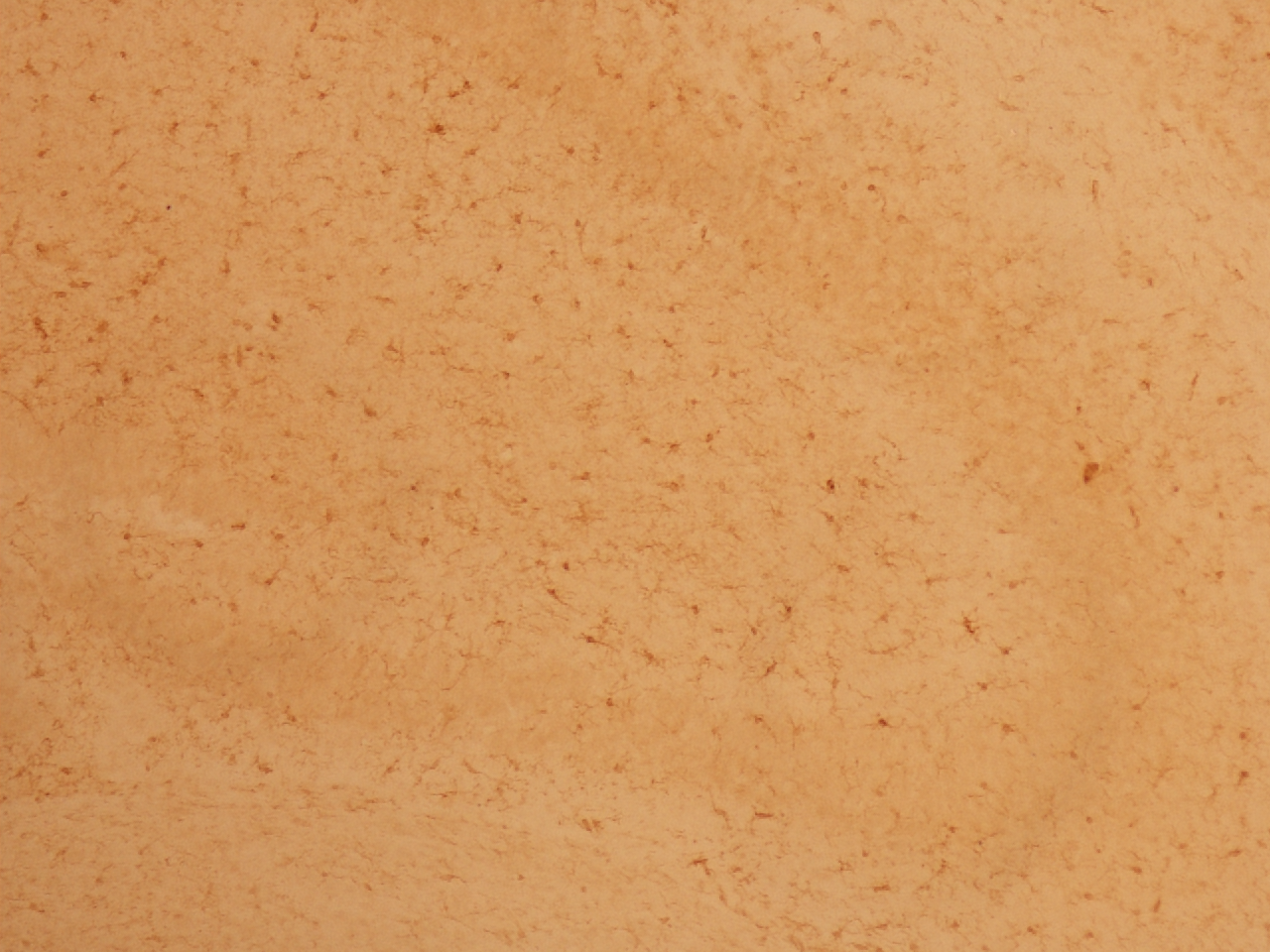

Supplement: Supplementary file 6 — Source data Fig. 5 [file 44321_2024_79_MOESM6_ESM.zip › Figure 5/5B/Hippocampus Saline.TIF]

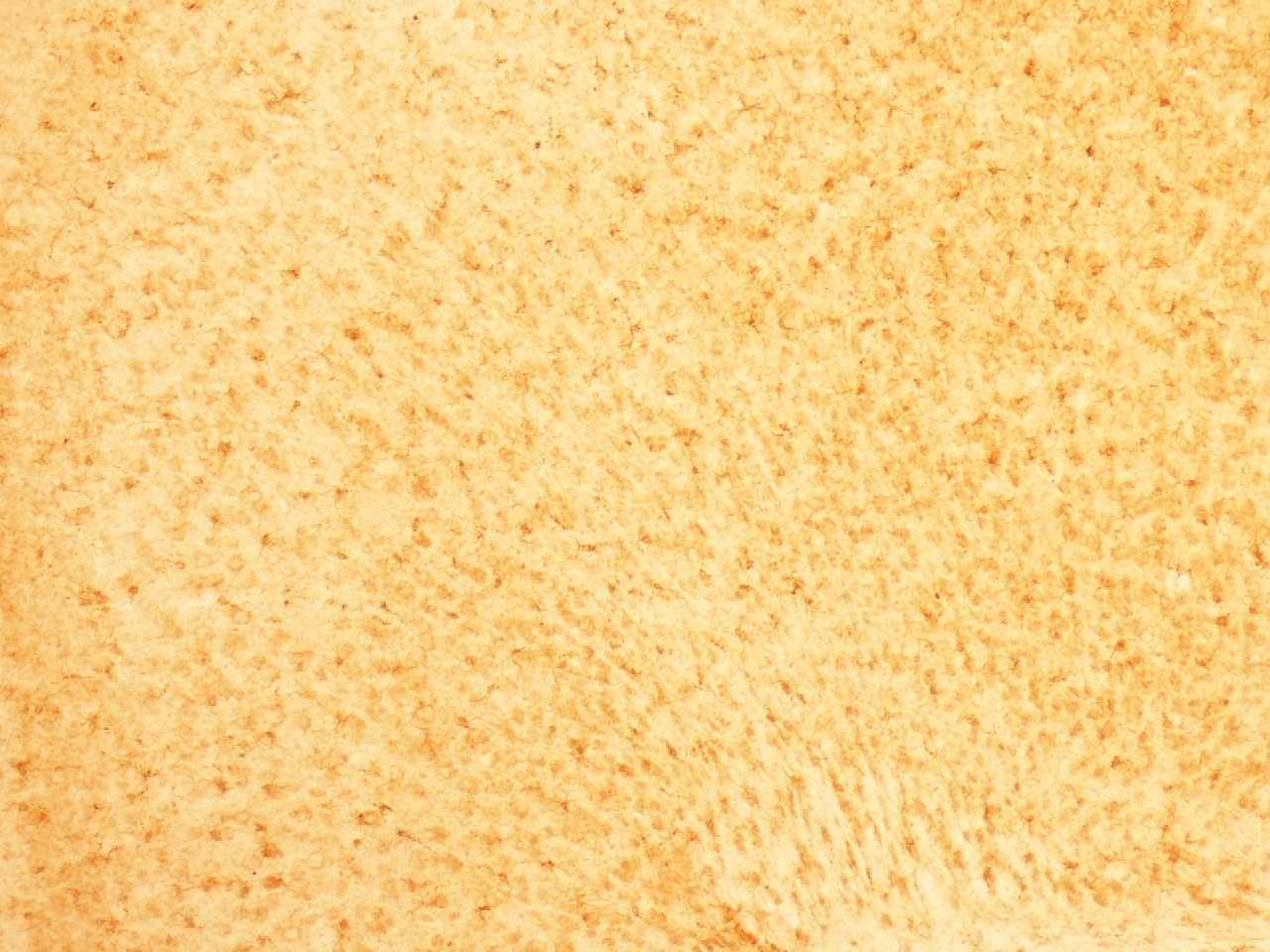

Supplement: Supplementary file 6 — Source data Fig. 5 [file 44321_2024_79_MOESM6_ESM.zip › Figure 5/5B/Cortex HI+Sem.TIF]

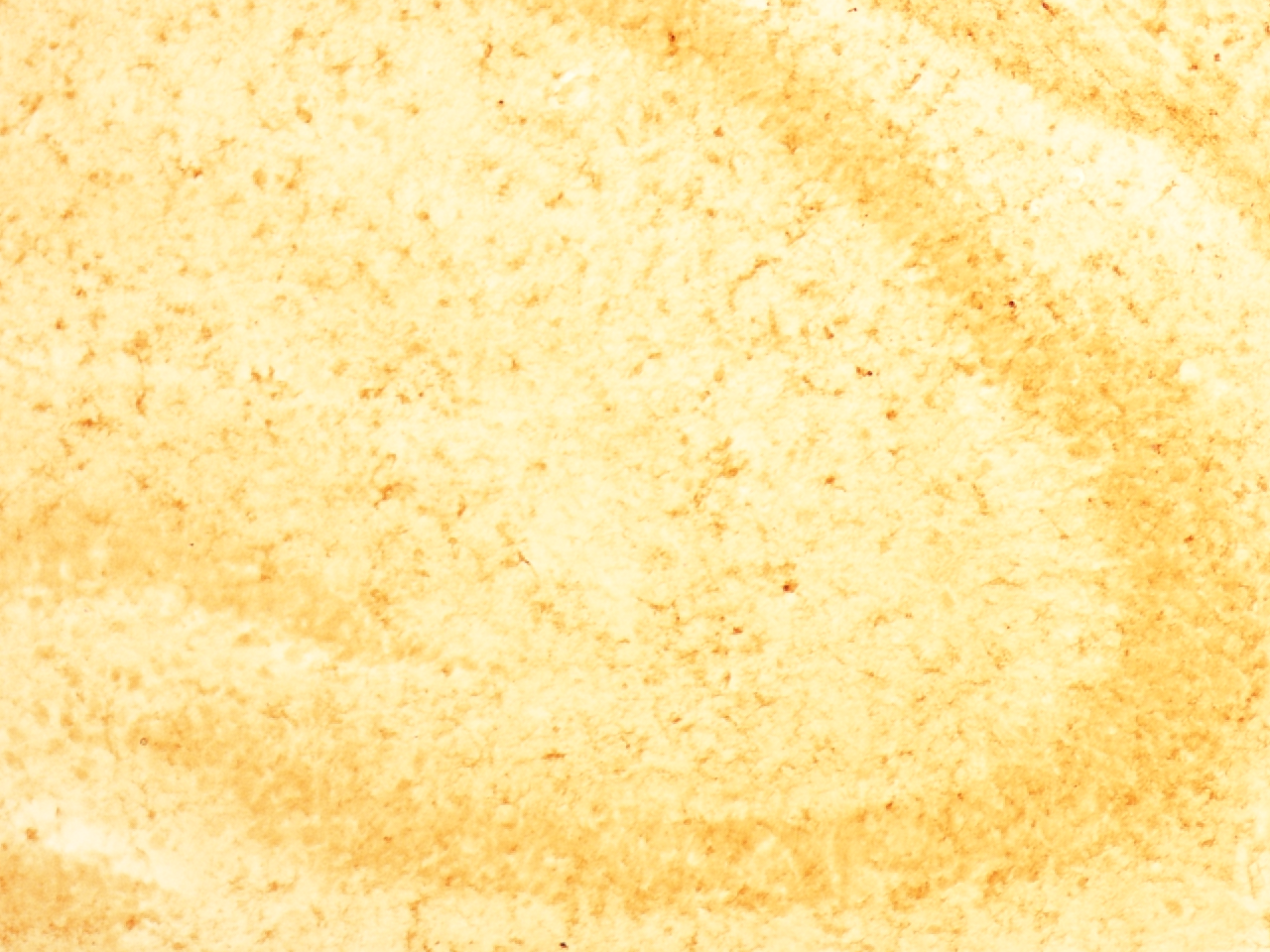

Supplement: Supplementary file 6 — Source data Fig. 5 [file 44321_2024_79_MOESM6_ESM.zip › Figure 5/5B/Hippocampus HI+Sem.TIF]

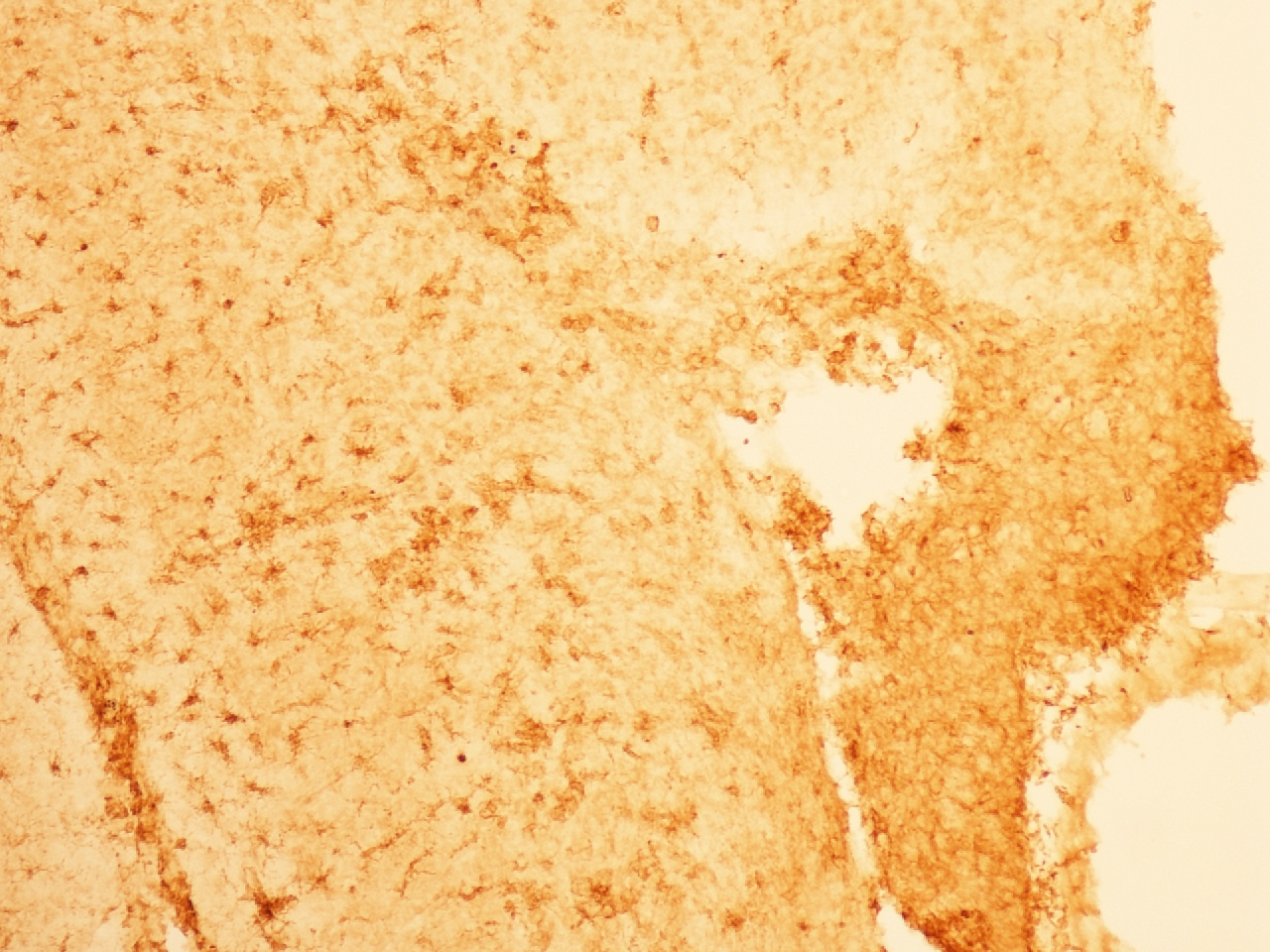

Supplement: Supplementary file 6 — Source data Fig. 5 [file 44321_2024_79_MOESM6_ESM.zip › Figure 5/5B/Cortex HI+Sal.TIF]

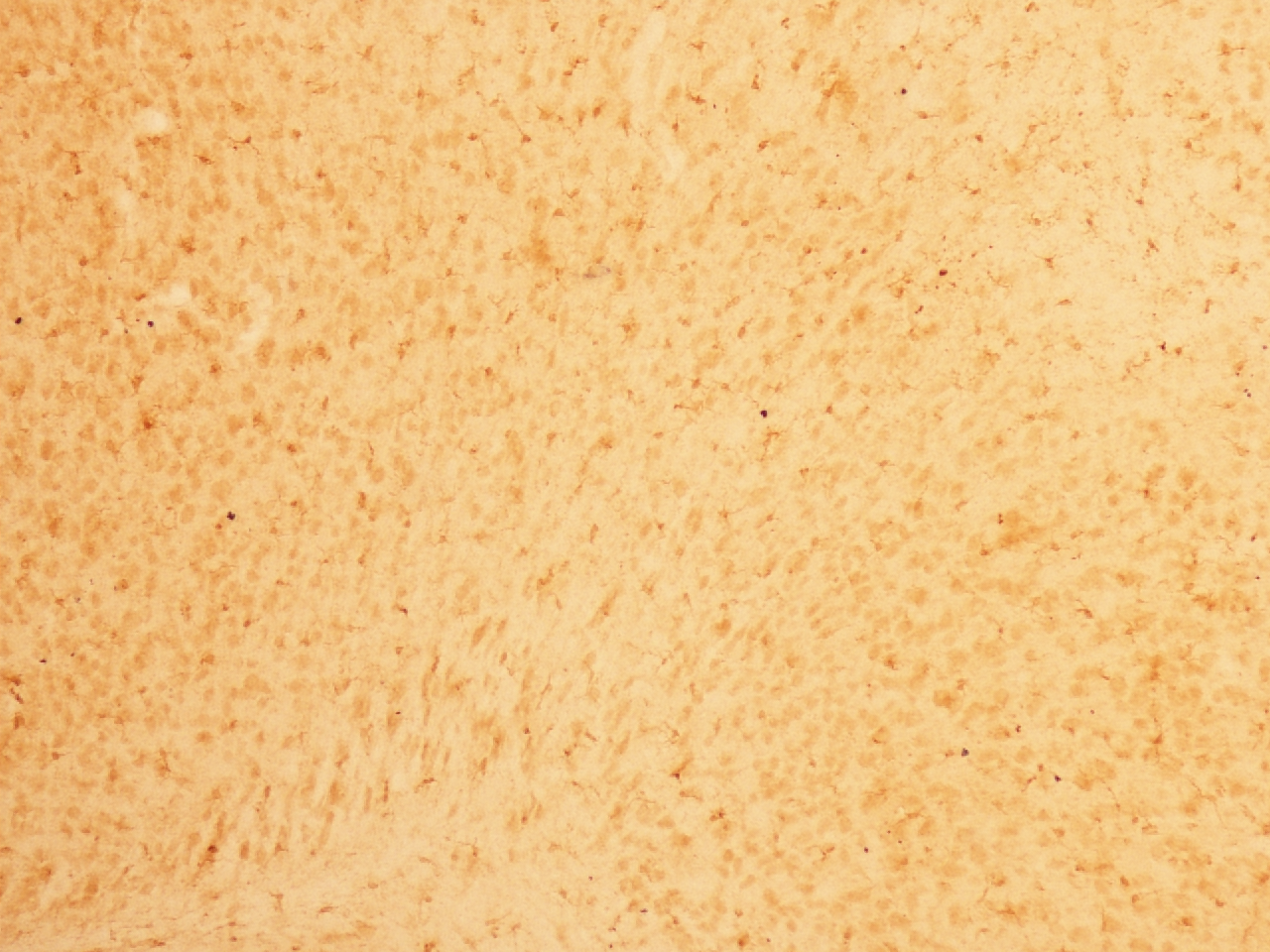

Supplement: Supplementary file 6 — Source data Fig. 5 [file 44321_2024_79_MOESM6_ESM.zip › Figure 5/5B/Cortex HI+Ex-4.TIF]

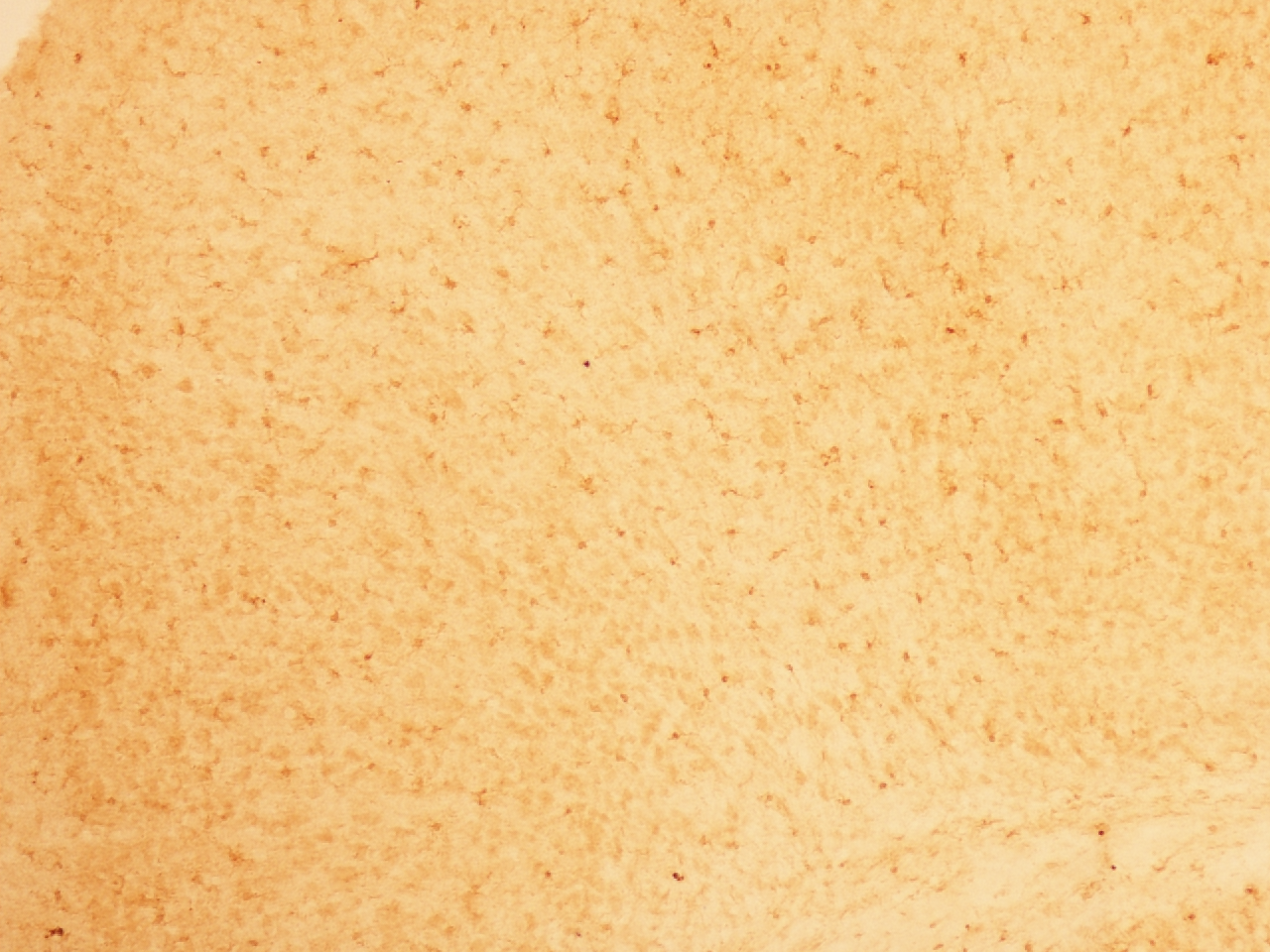

Supplement: Supplementary file 6 — Source data Fig. 5 [file 44321_2024_79_MOESM6_ESM.zip › Figure 5/5B/Cortex Saline.TIF]

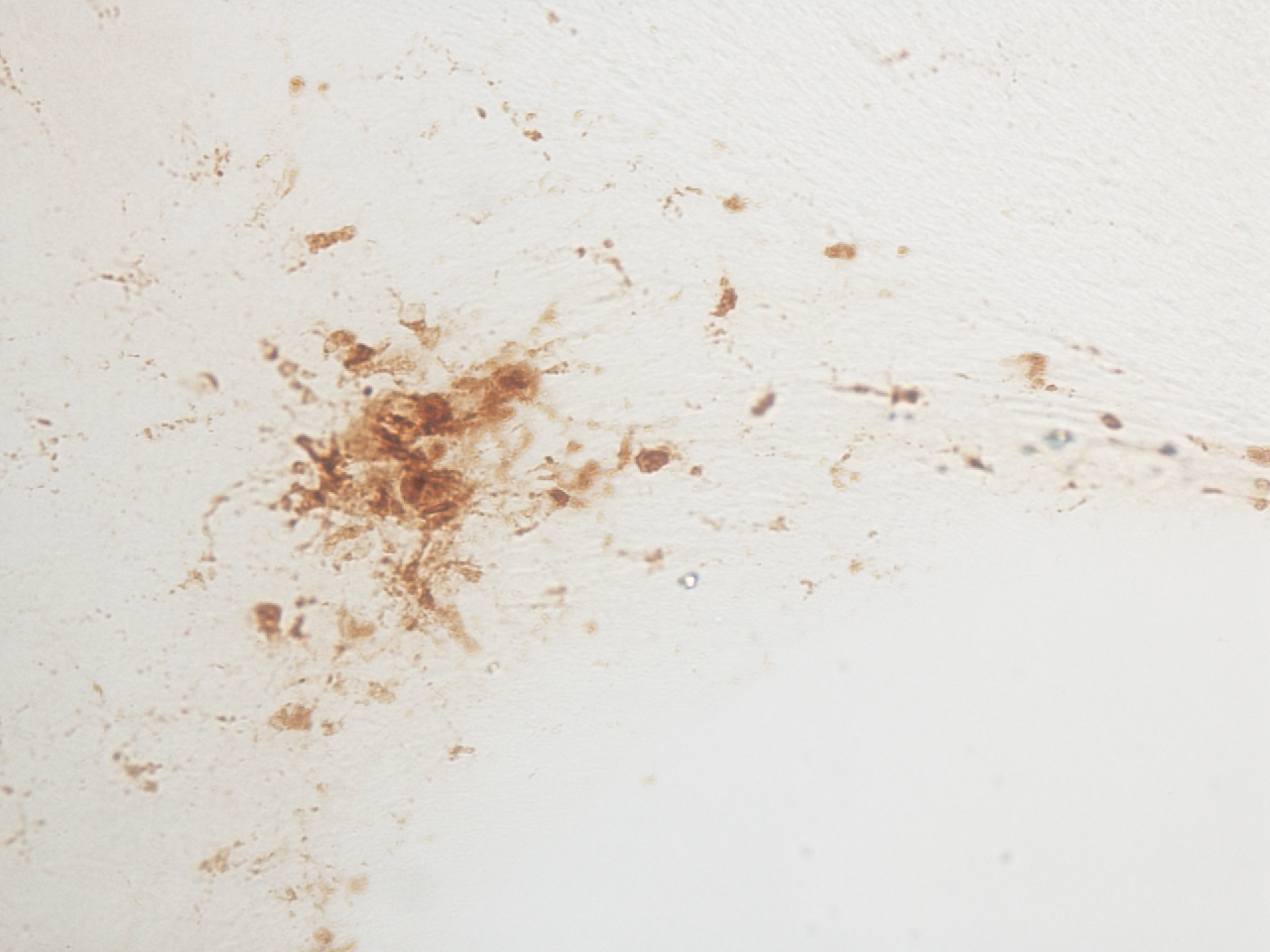

Supplement: Supplementary file 7 — Source data Fig. 6 [file 44321_2024_79_MOESM7_ESM.zip › Figure 6/6A/Hippocampus HI+Sal.TIF]

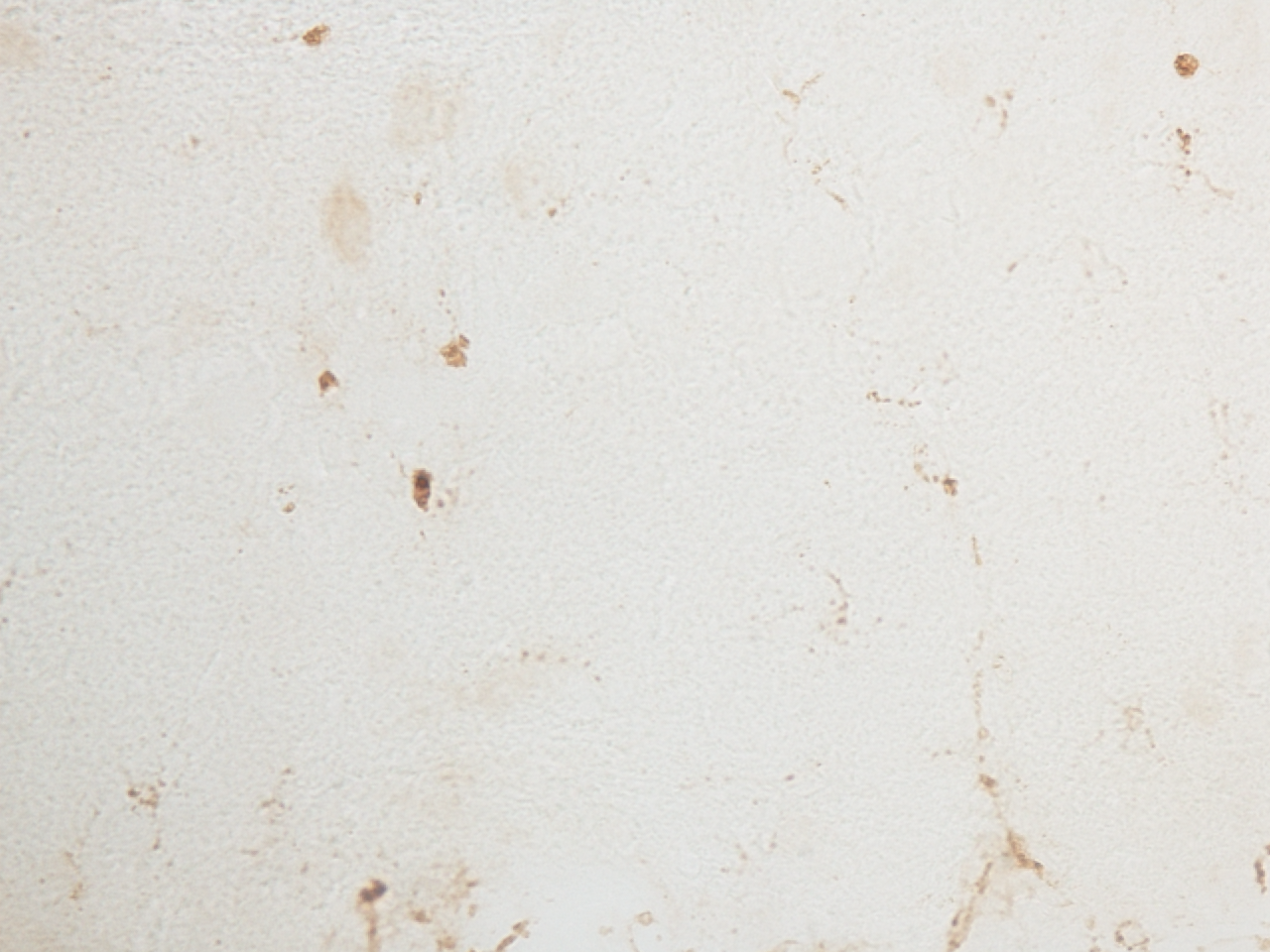

Supplement: Supplementary file 7 — Source data Fig. 6 [file 44321_2024_79_MOESM7_ESM.zip › Figure 6/6A/Hippocampus HI+Ex-4.TIF]

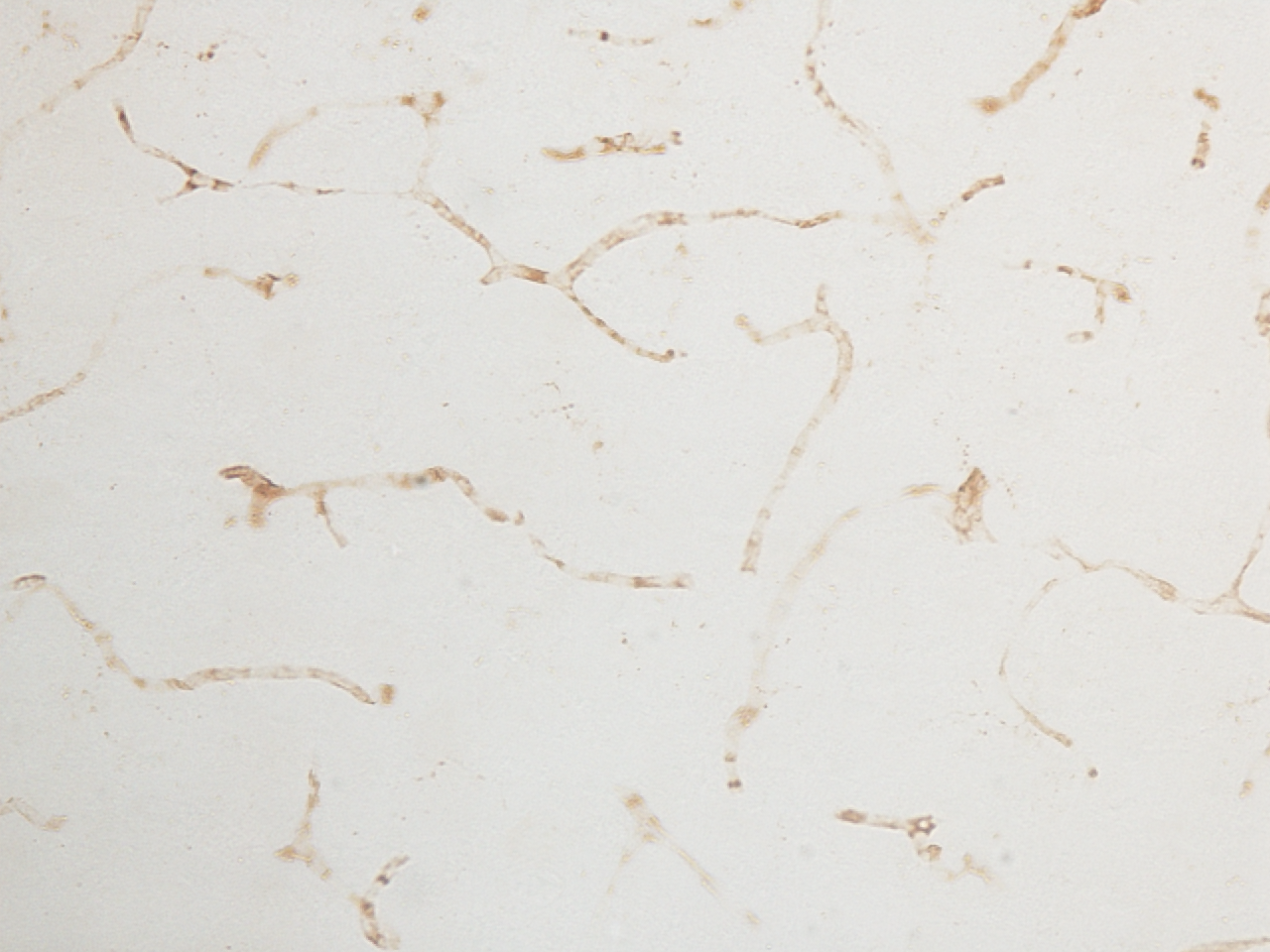

Supplement: Supplementary file 7 — Source data Fig. 6 [file 44321_2024_79_MOESM7_ESM.zip › Figure 6/6A/Cortex HI+Sema.TIF]

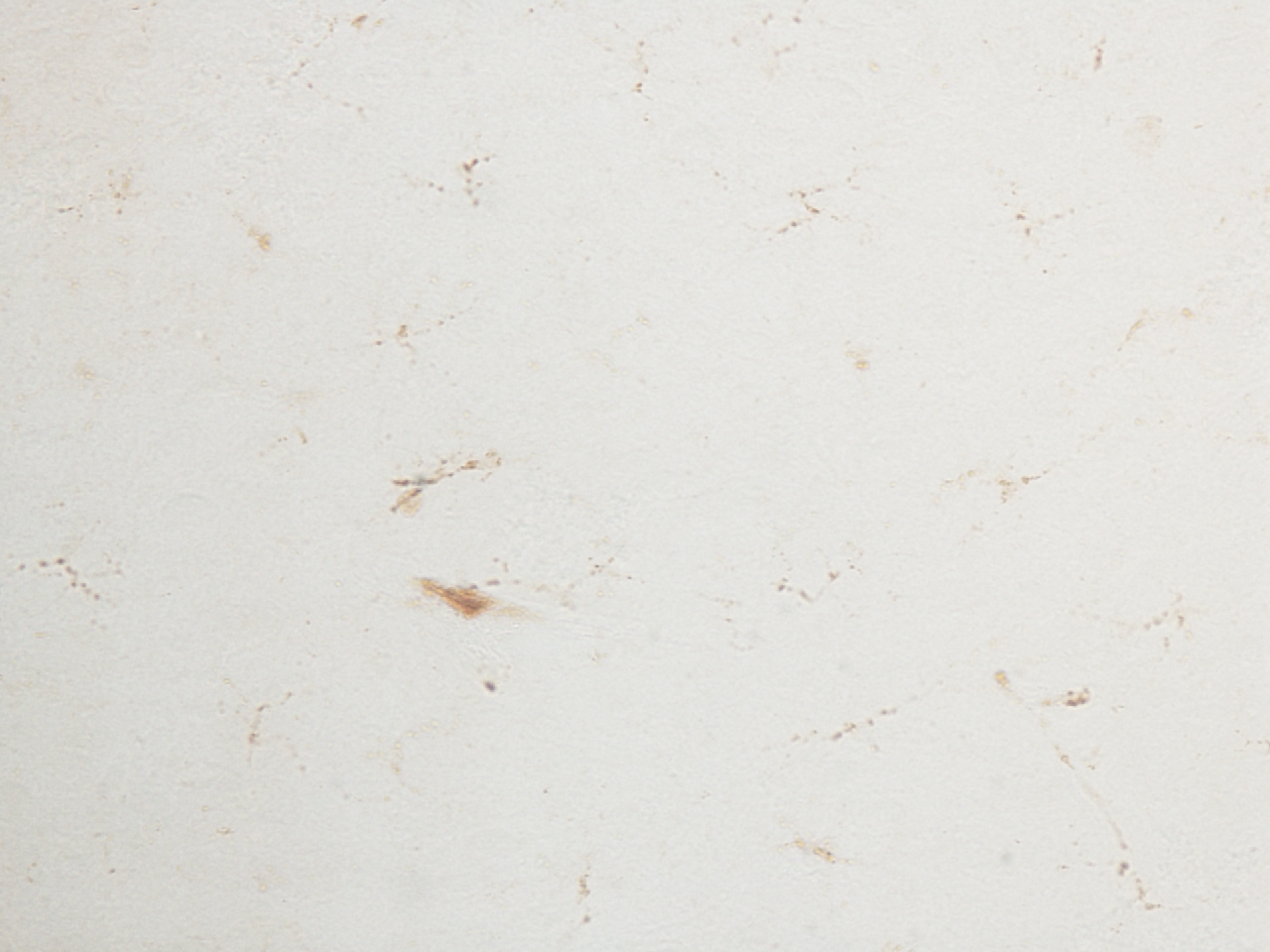

Supplement: Supplementary file 7 — Source data Fig. 6 [file 44321_2024_79_MOESM7_ESM.zip › Figure 6/6A/Hippocampus Sal.TIF]

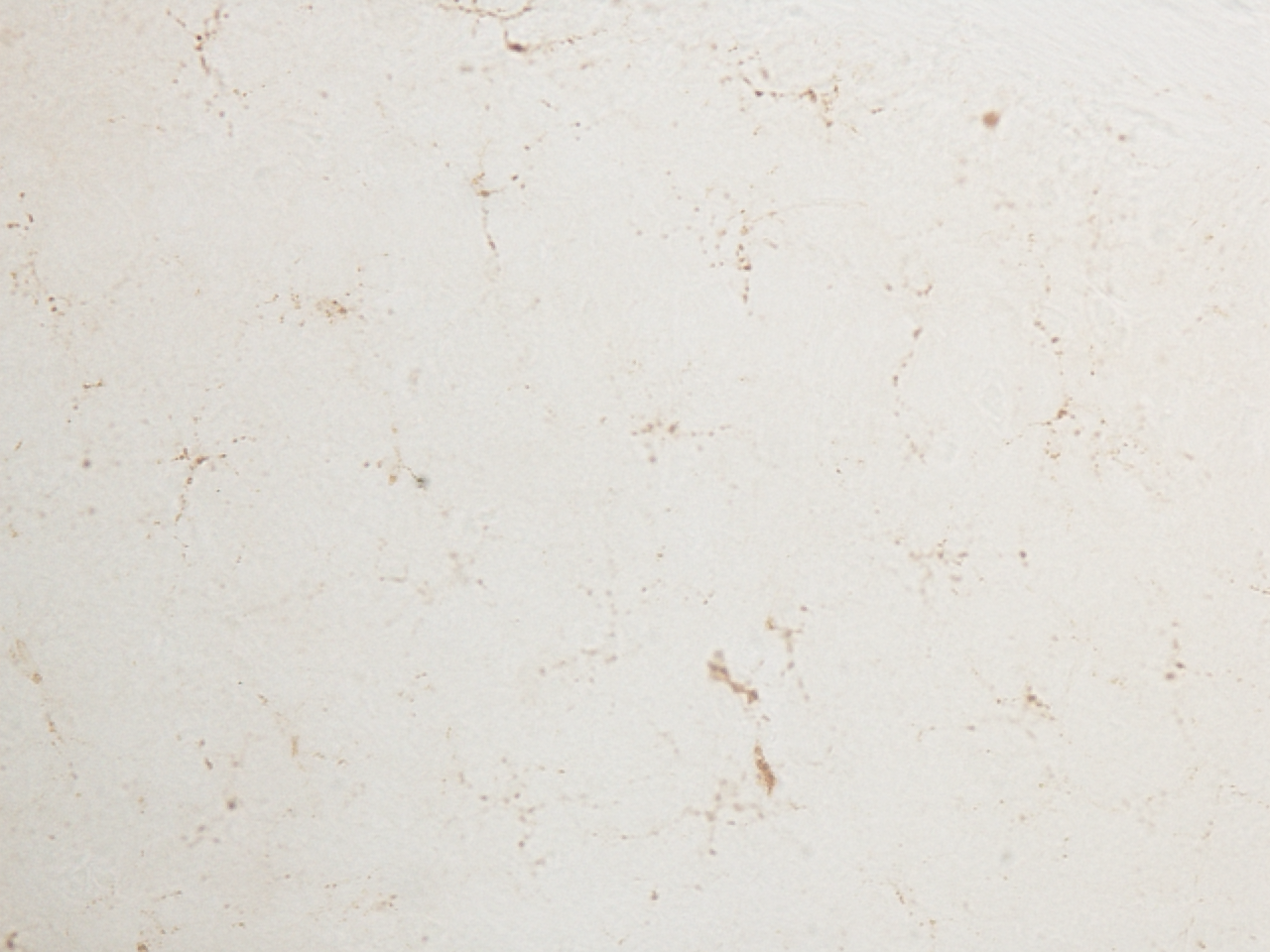

Supplement: Supplementary file 7 — Source data Fig. 6 [file 44321_2024_79_MOESM7_ESM.zip › Figure 6/6A/Hippocampus HI+Sema.TIF]

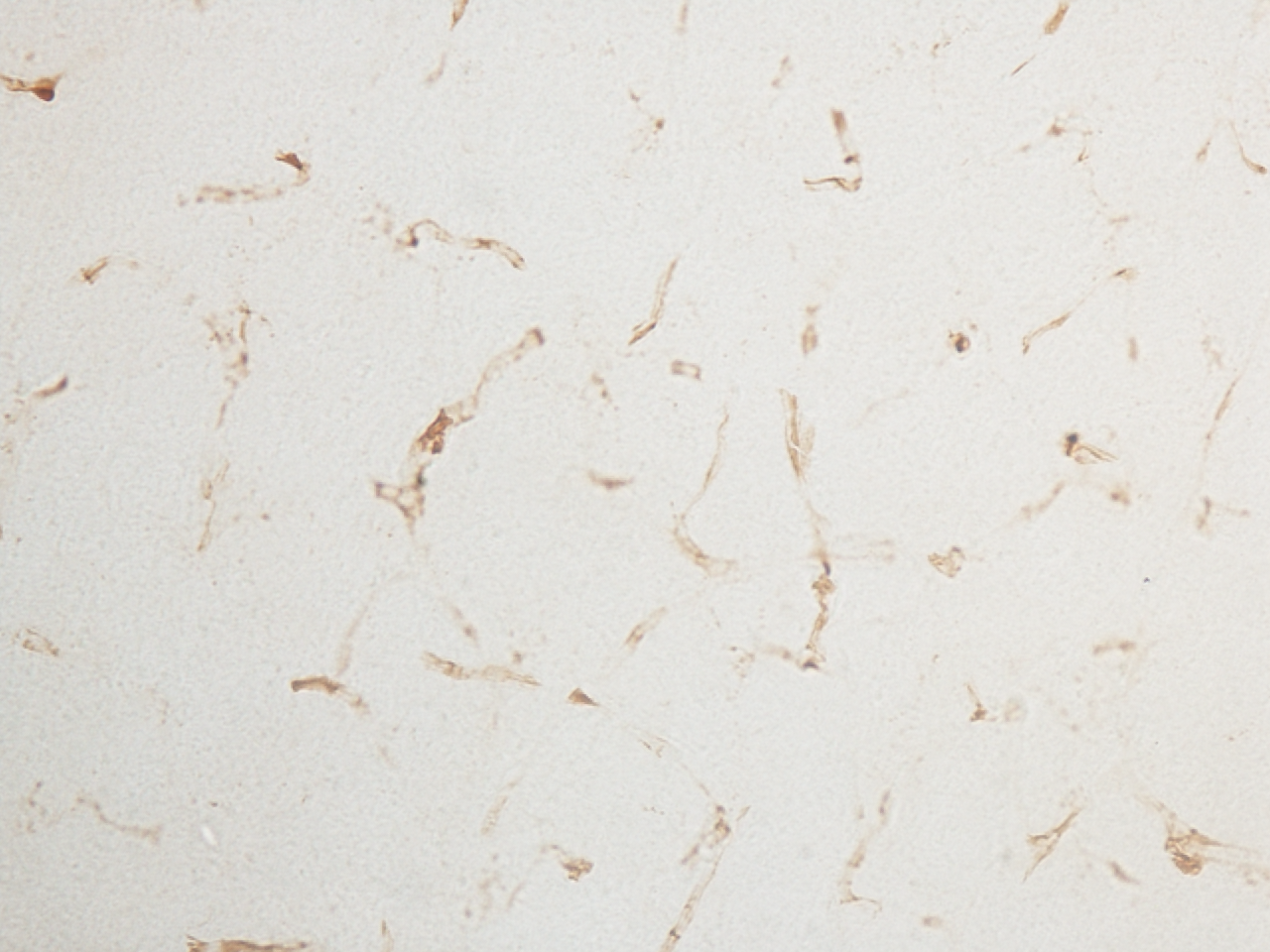

Supplement: Supplementary file 7 — Source data Fig. 6 [file 44321_2024_79_MOESM7_ESM.zip › Figure 6/6A/Cortex HI+Sal.TIF]

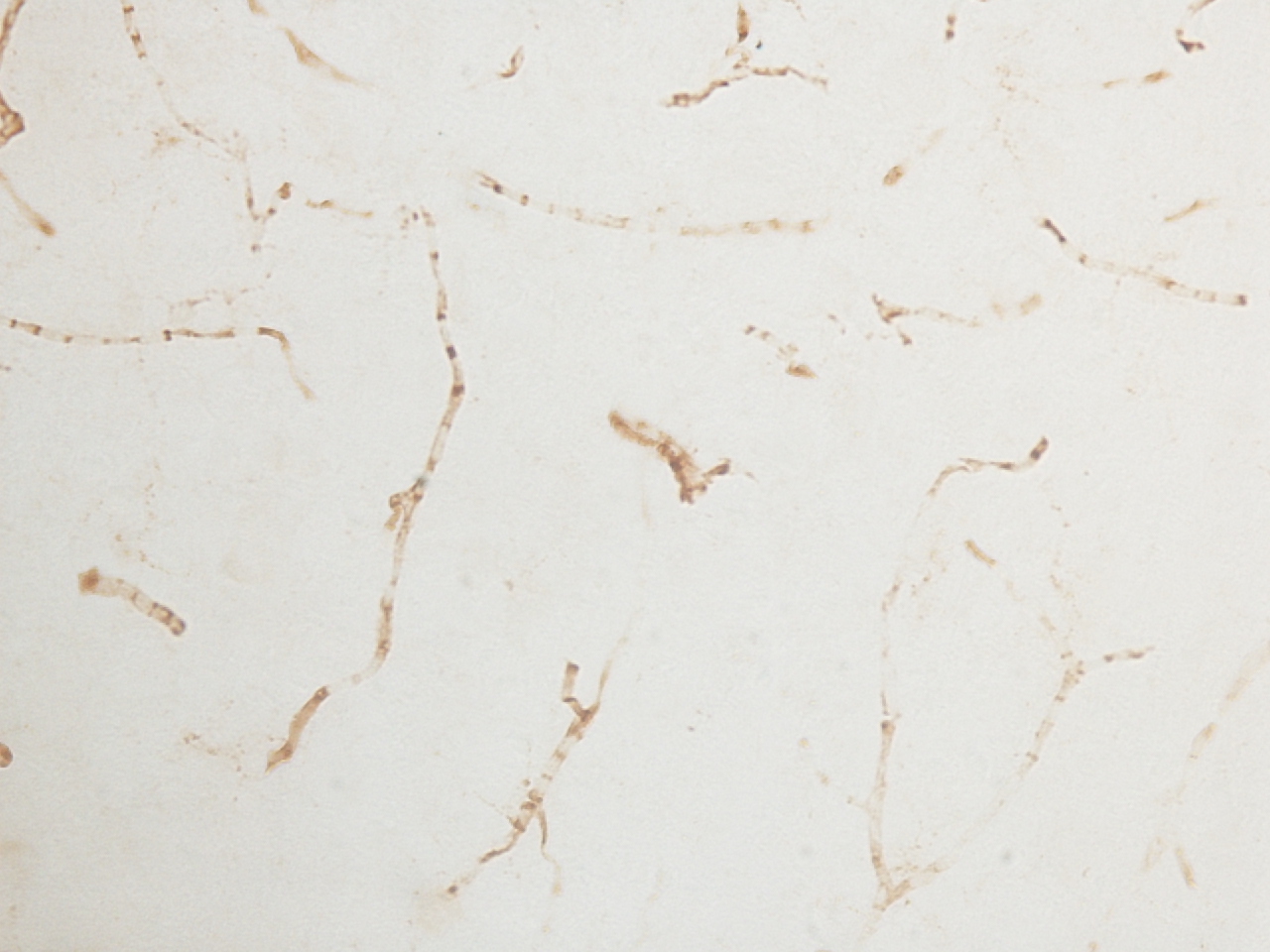

Supplement: Supplementary file 7 — Source data Fig. 6 [file 44321_2024_79_MOESM7_ESM.zip › Figure 6/6A/Cortex Sal.TIF]

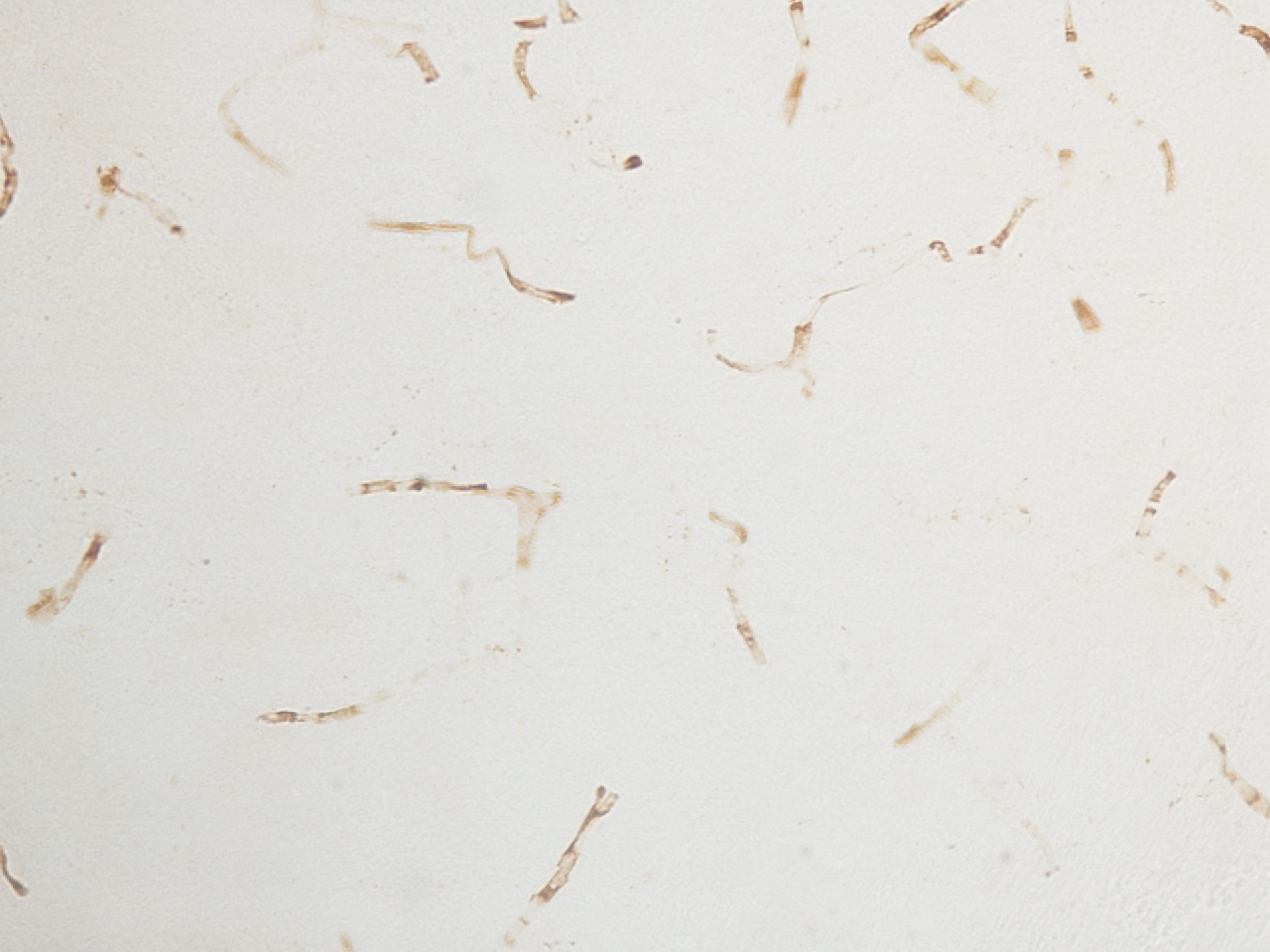

Supplement: Supplementary file 7 — Source data Fig. 6 [file 44321_2024_79_MOESM7_ESM.zip › Figure 6/6A/Cortex HI+Ex-4.TIF]

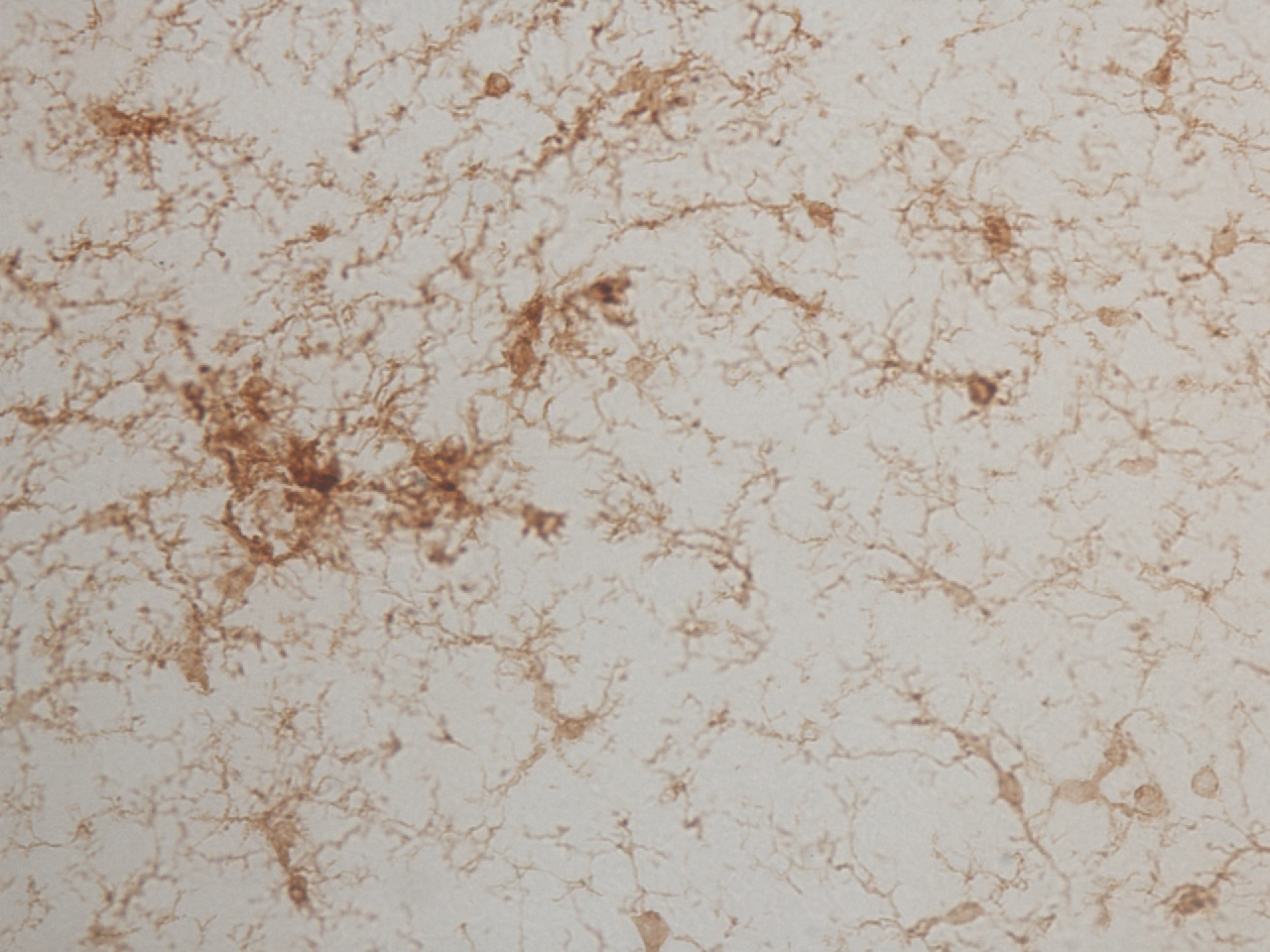

Supplement: Supplementary file 7 — Source data Fig. 6 [file 44321_2024_79_MOESM7_ESM.zip › Figure 6/6B/Hippocampus HI+Sal.TIF]

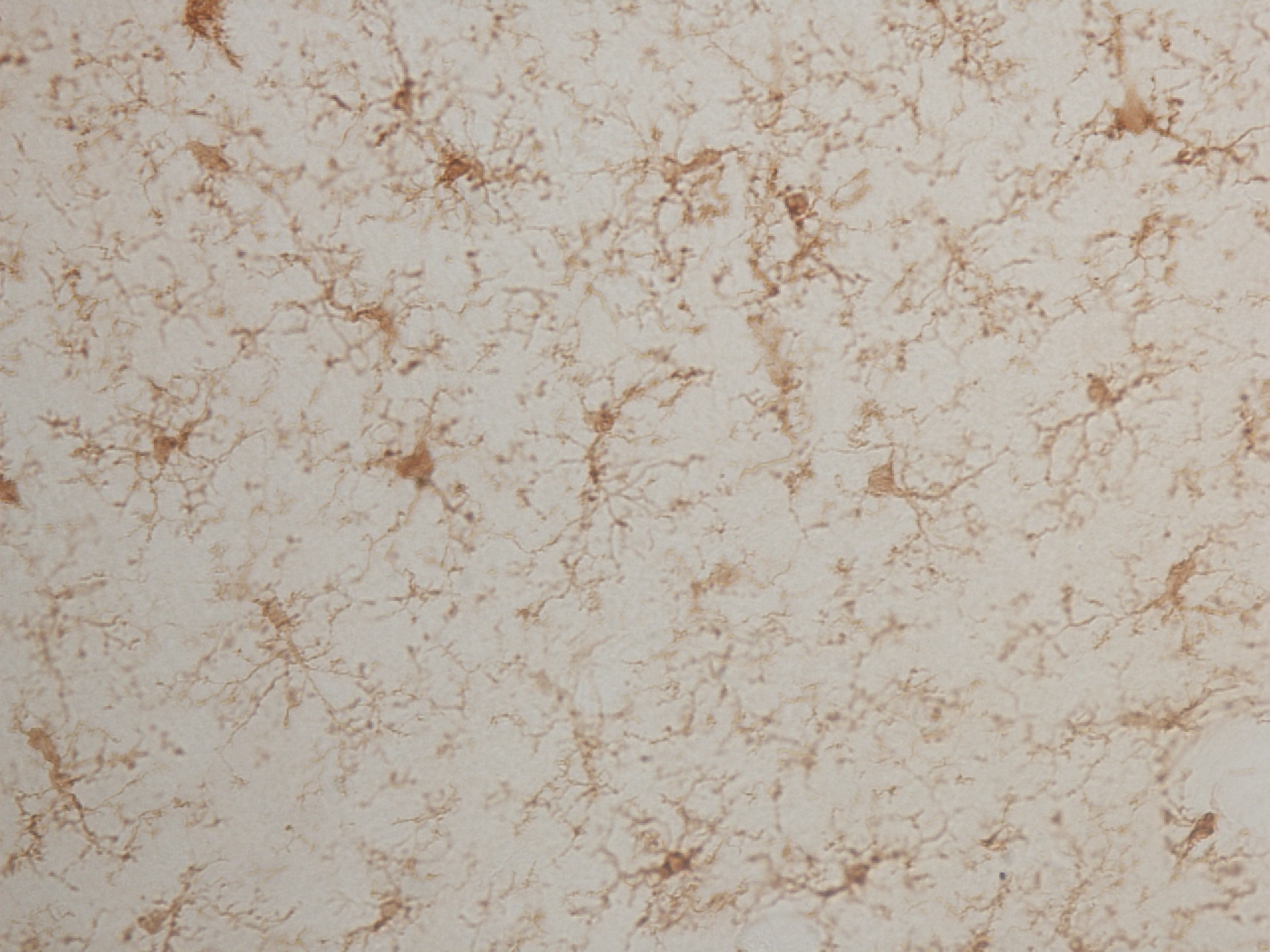

Supplement: Supplementary file 7 — Source data Fig. 6 [file 44321_2024_79_MOESM7_ESM.zip › Figure 6/6B/Hippocampus HI+Ex-4.TIF]

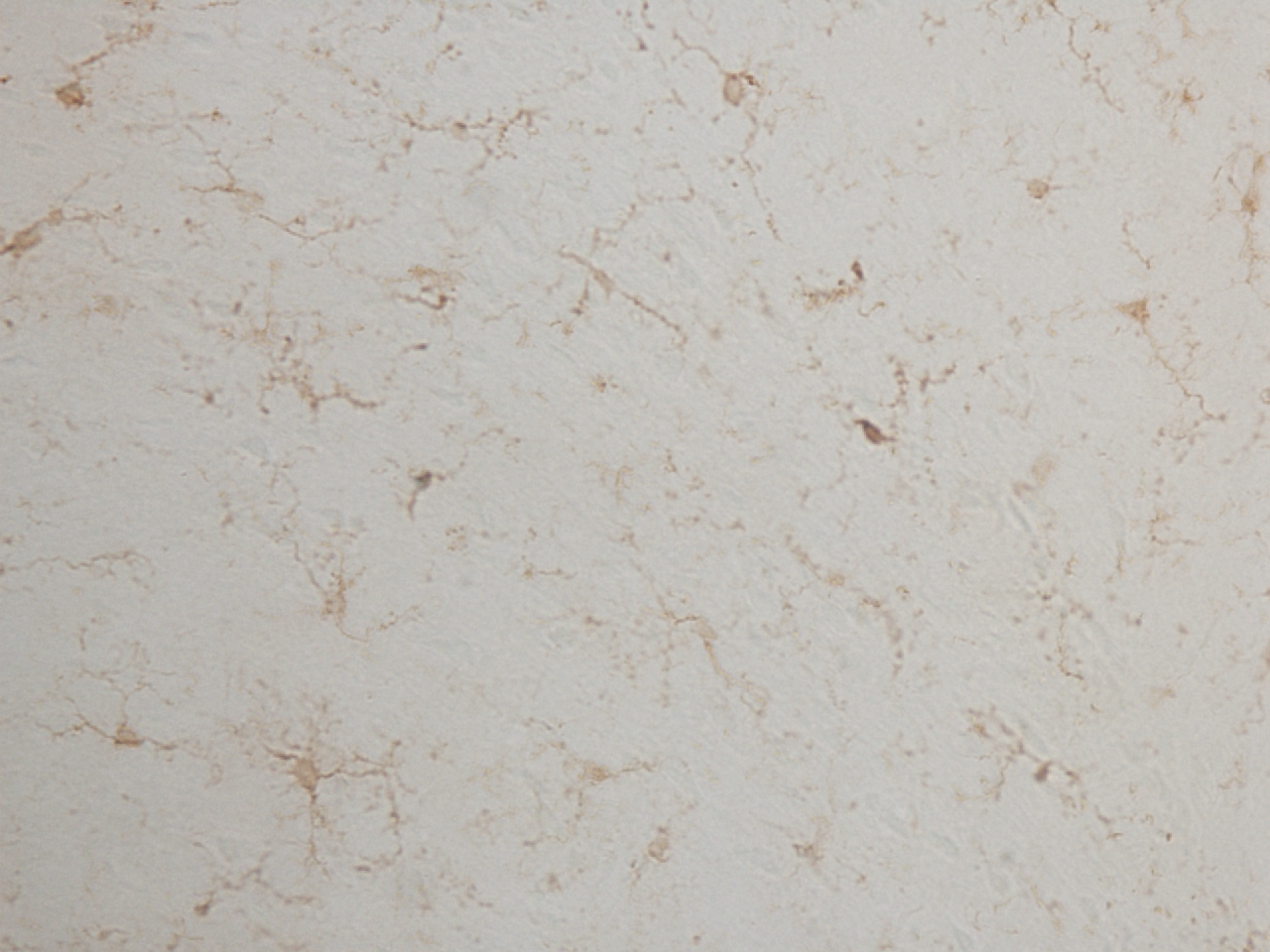

Supplement: Supplementary file 7 — Source data Fig. 6 [file 44321_2024_79_MOESM7_ESM.zip › Figure 6/6B/Cortex HI+Sema.TIF]

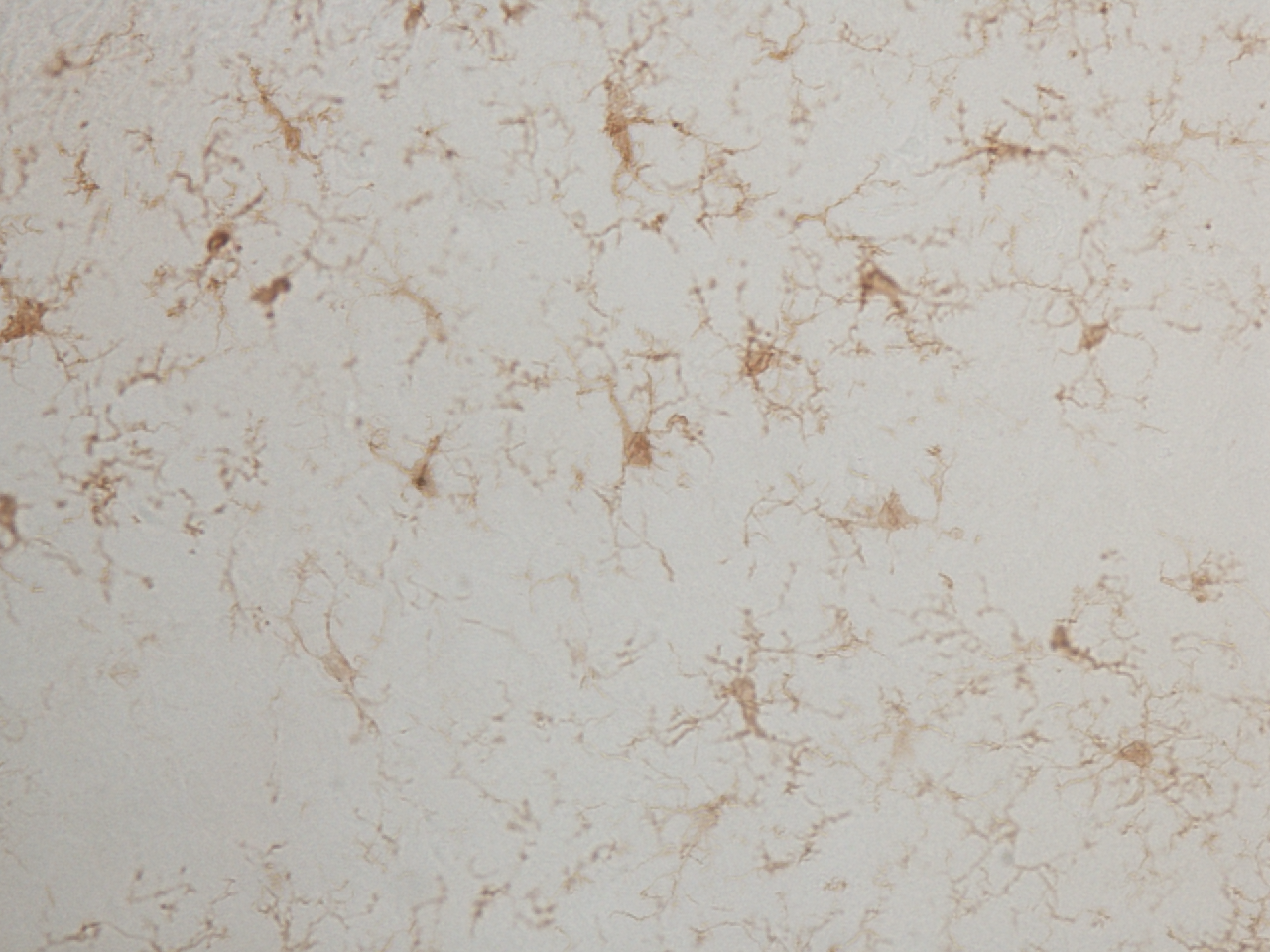

Supplement: Supplementary file 7 — Source data Fig. 6 [file 44321_2024_79_MOESM7_ESM.zip › Figure 6/6B/Hippocampus Sal.TIF]

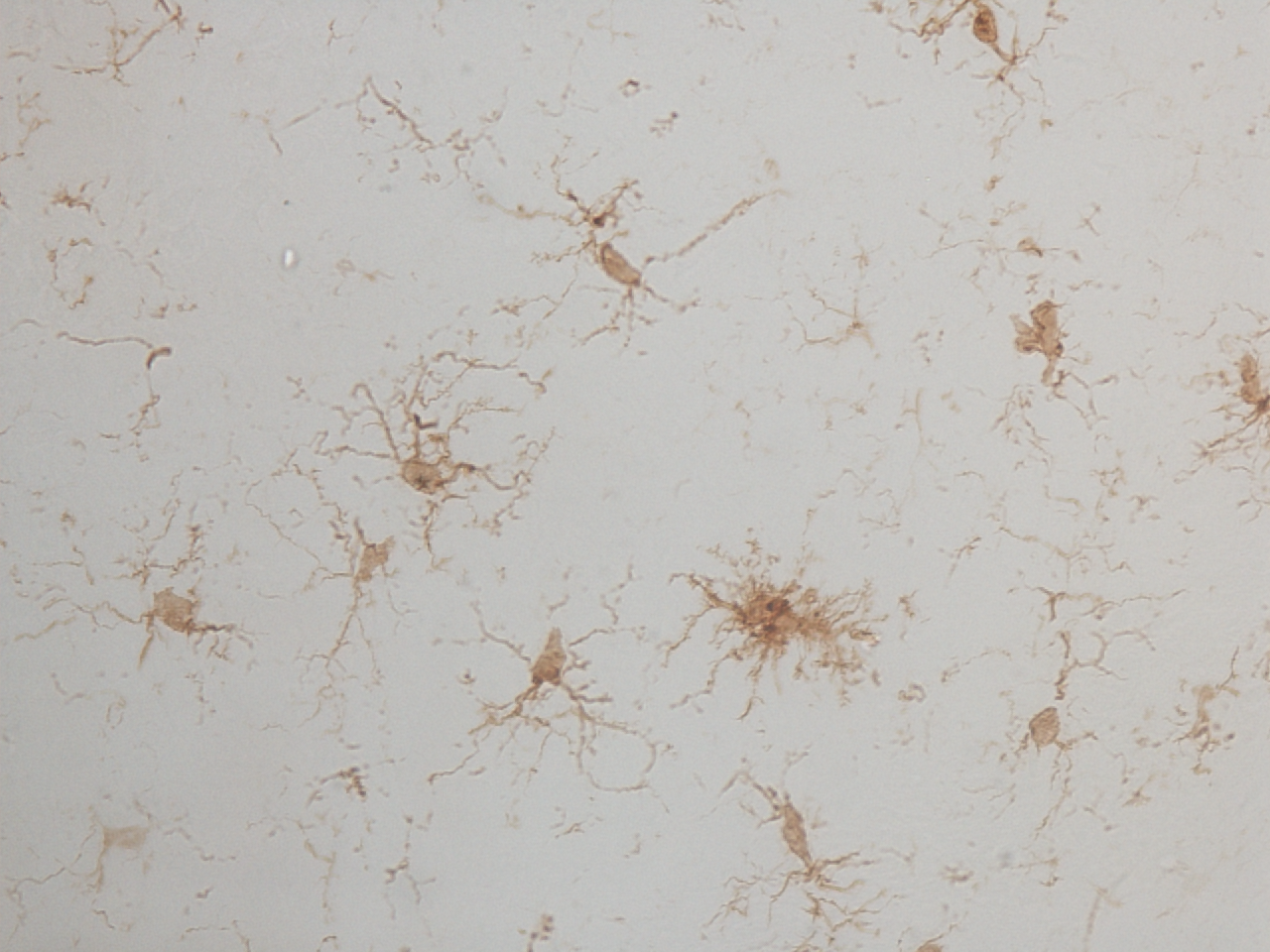

Supplement: Supplementary file 7 — Source data Fig. 6 [file 44321_2024_79_MOESM7_ESM.zip › Figure 6/6B/Hippocampus HI+Sema.TIF]

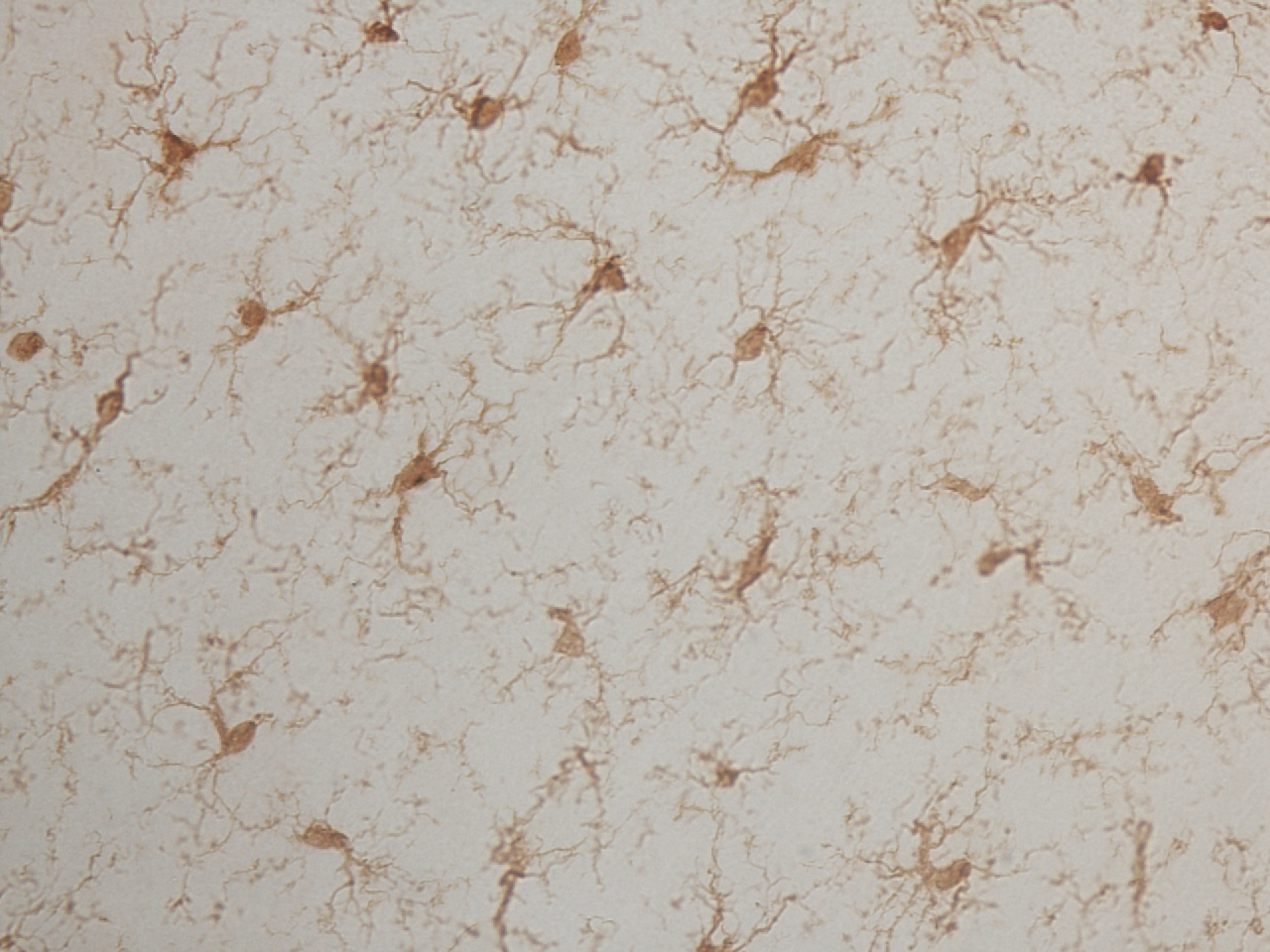

Supplement: Supplementary file 7 — Source data Fig. 6 [file 44321_2024_79_MOESM7_ESM.zip › Figure 6/6B/Cortex HI+Sal.TIF]

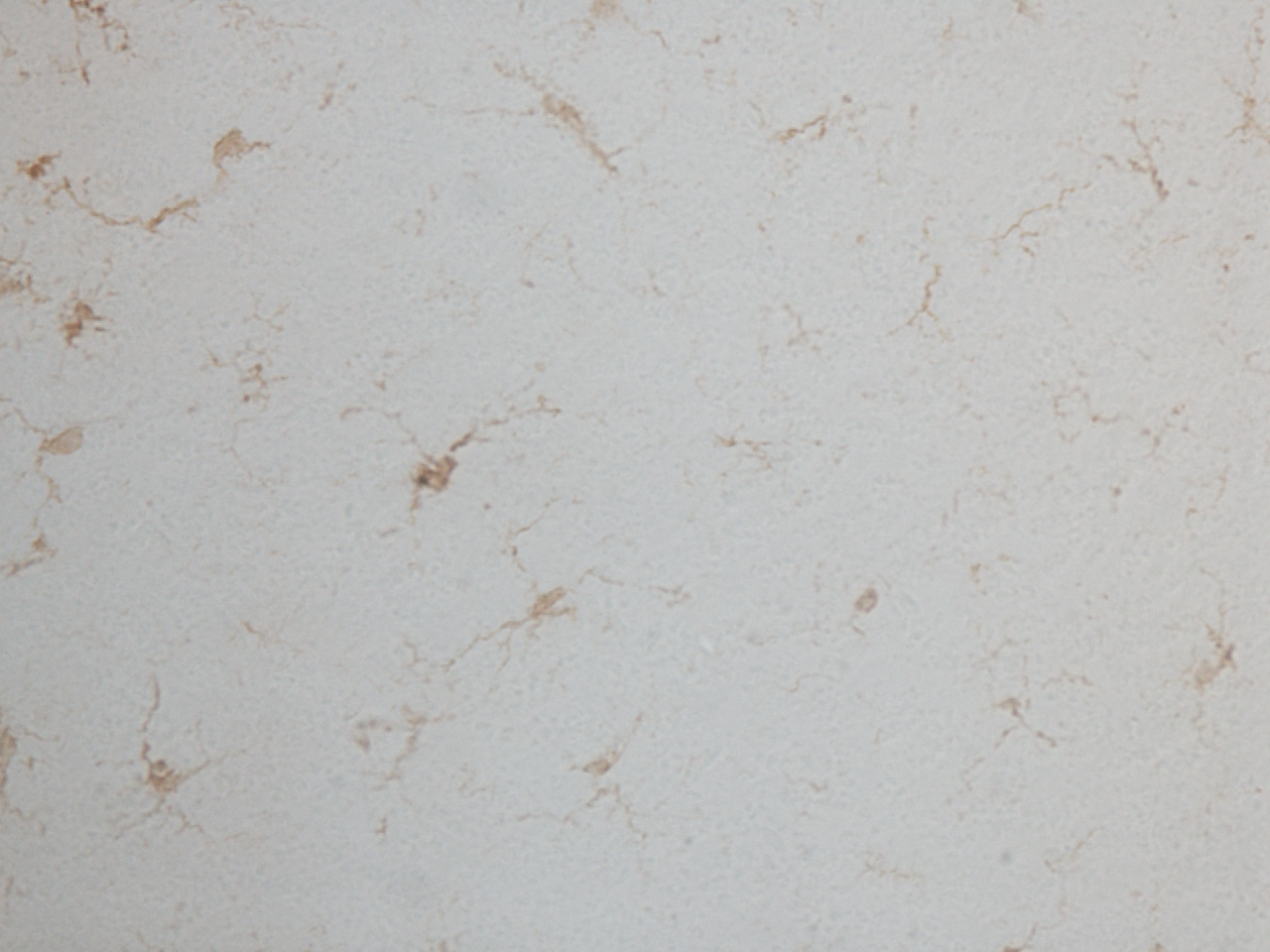

Supplement: Supplementary file 7 — Source data Fig. 6 [file 44321_2024_79_MOESM7_ESM.zip › Figure 6/6B/Cortex Sal.TIF]

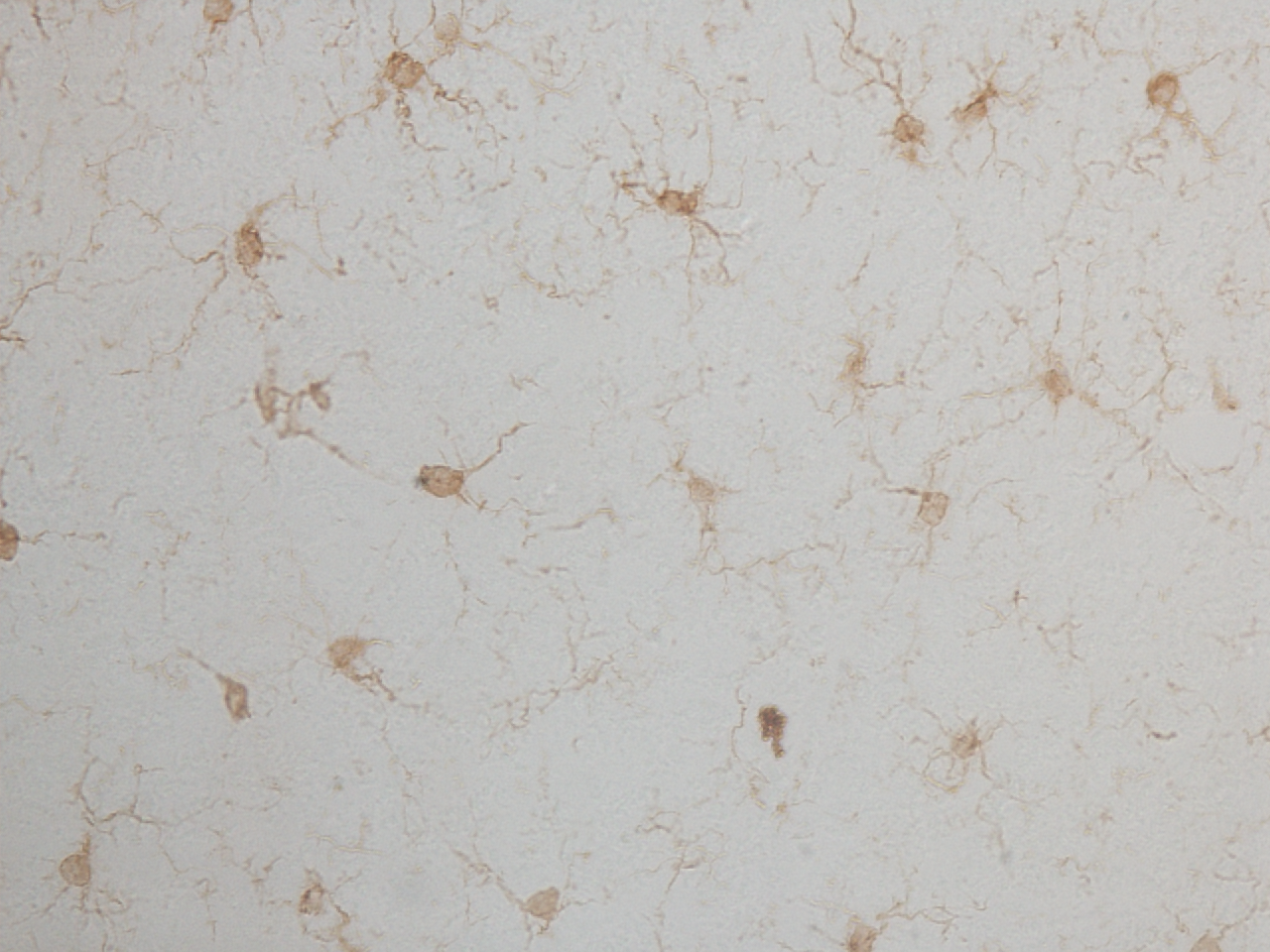

Supplement: Supplementary file 7 — Source data Fig. 6 [file 44321_2024_79_MOESM7_ESM.zip › Figure 6/6B/Cortex HI+Ex-4.TIF]

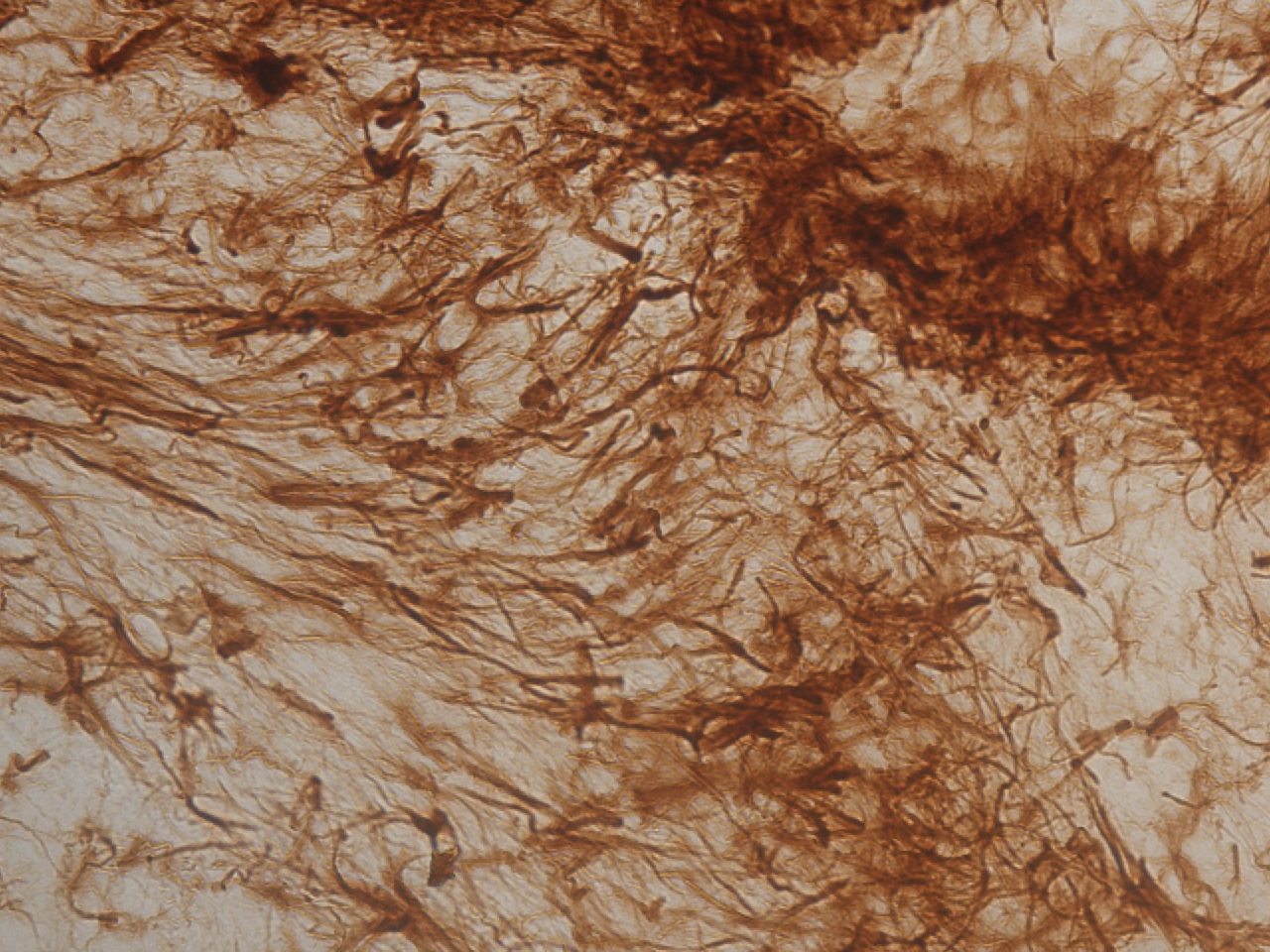

Supplement: Supplementary file 7 — Source data Fig. 6 [file 44321_2024_79_MOESM7_ESM.zip › Figure 6/6C/Hippocampus HI+Sal.TIF]

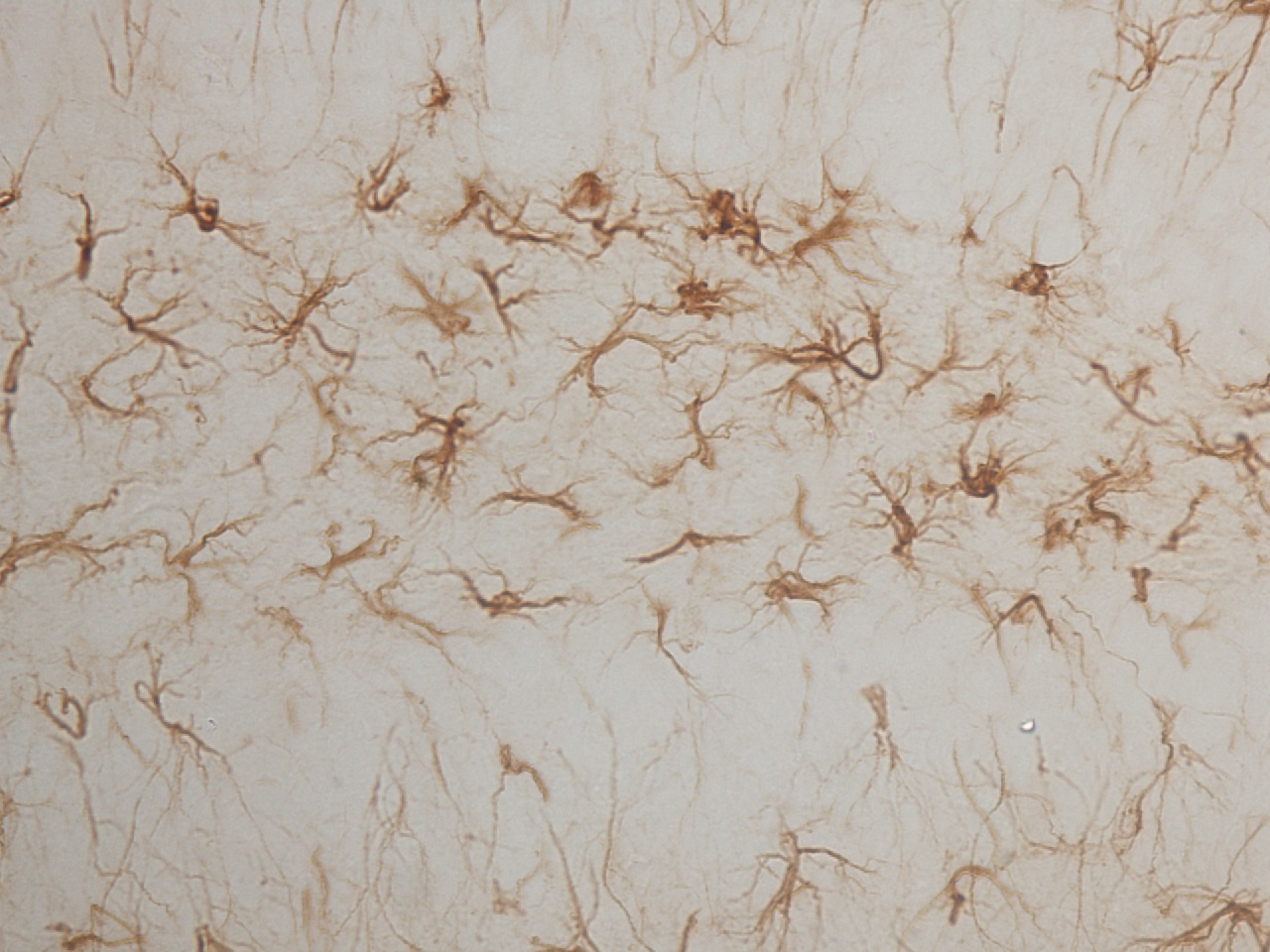

Supplement: Supplementary file 7 — Source data Fig. 6 [file 44321_2024_79_MOESM7_ESM.zip › Figure 6/6C/Hippocampus HI+Ex-4.TIF]

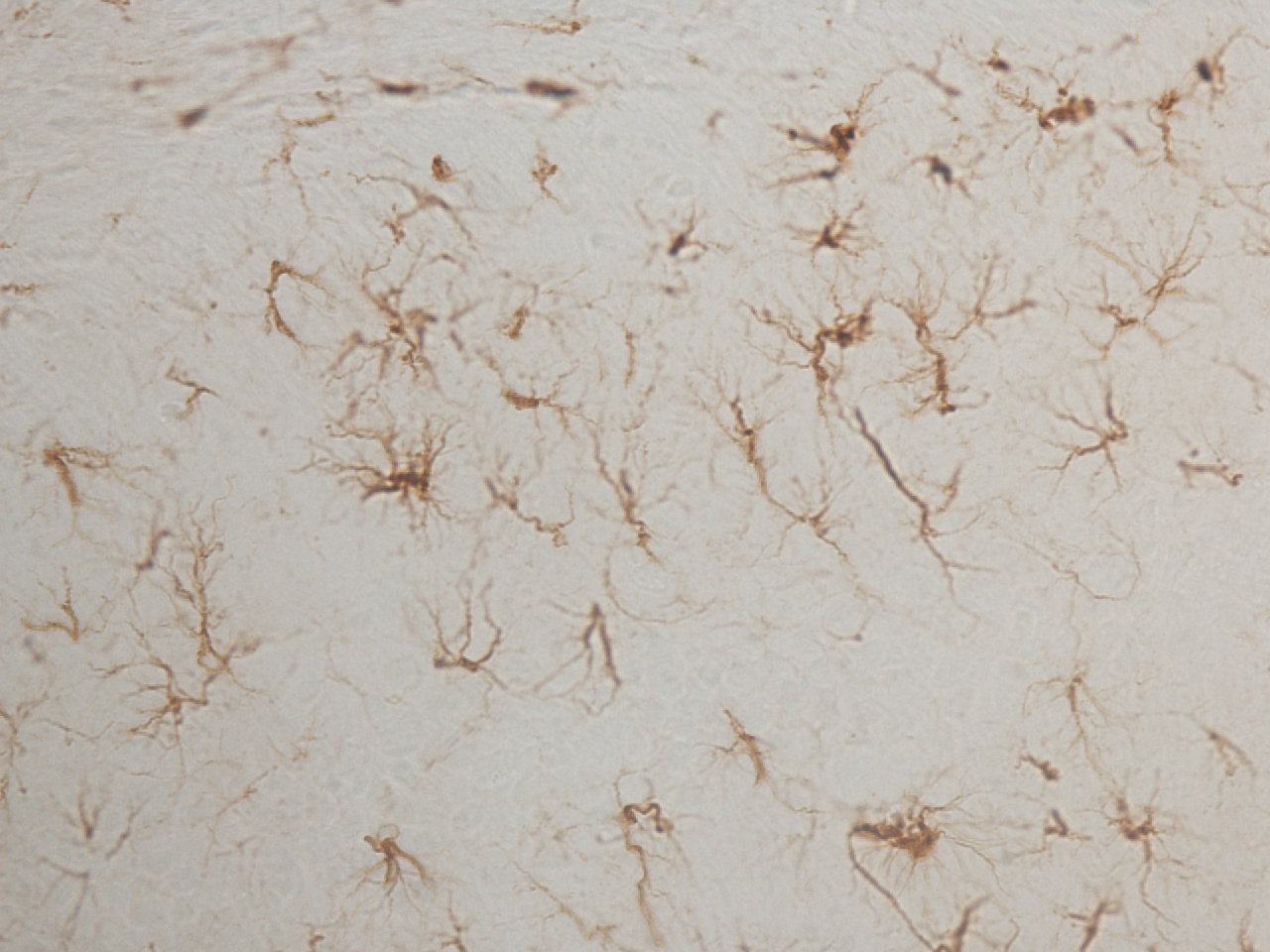

Supplement: Supplementary file 7 — Source data Fig. 6 [file 44321_2024_79_MOESM7_ESM.zip › Figure 6/6C/Hippocampus Saline.TIF]

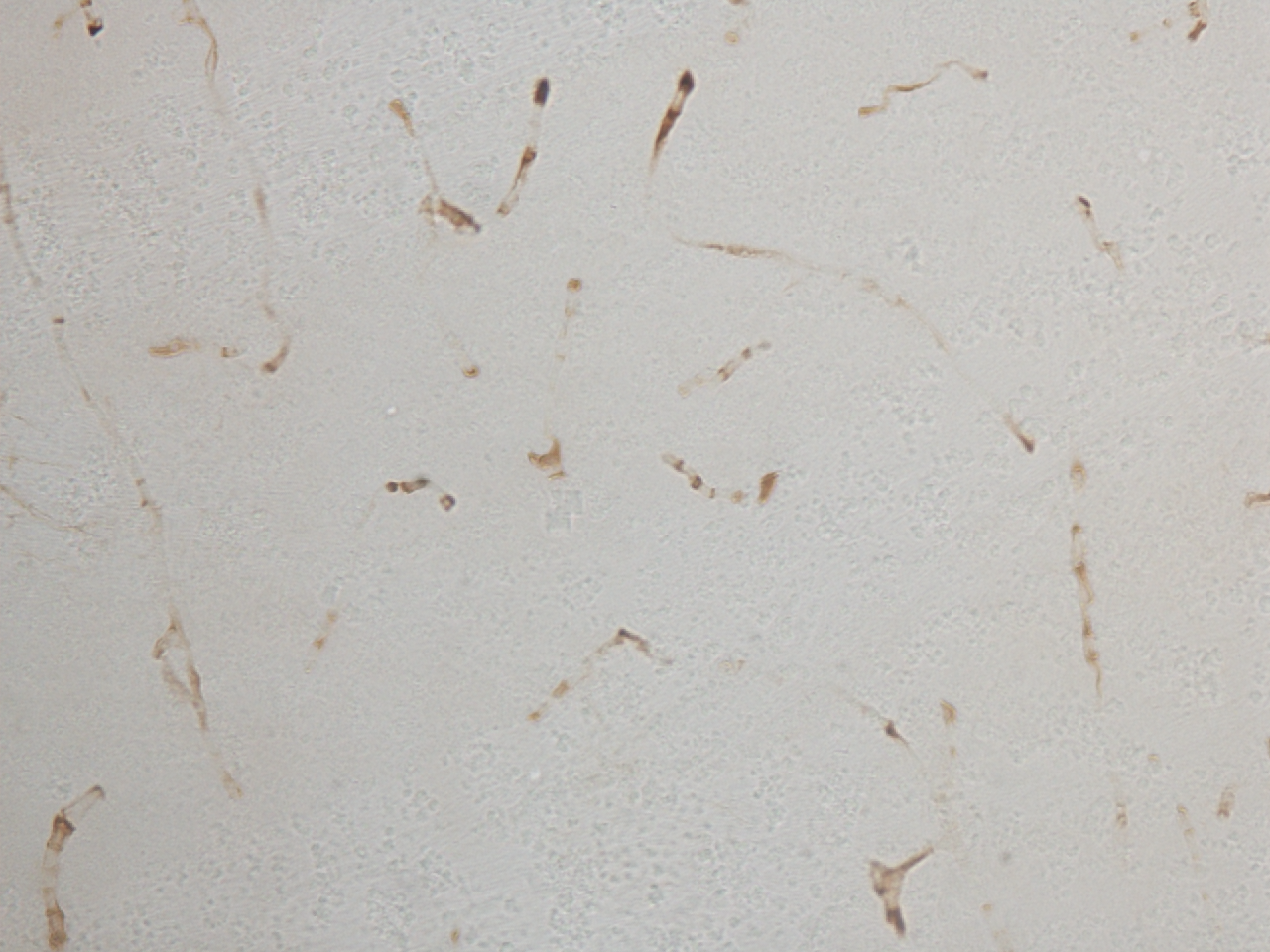

Supplement: Supplementary file 7 — Source data Fig. 6 [file 44321_2024_79_MOESM7_ESM.zip › Figure 6/6C/Cortex HI+Sema.TIF]

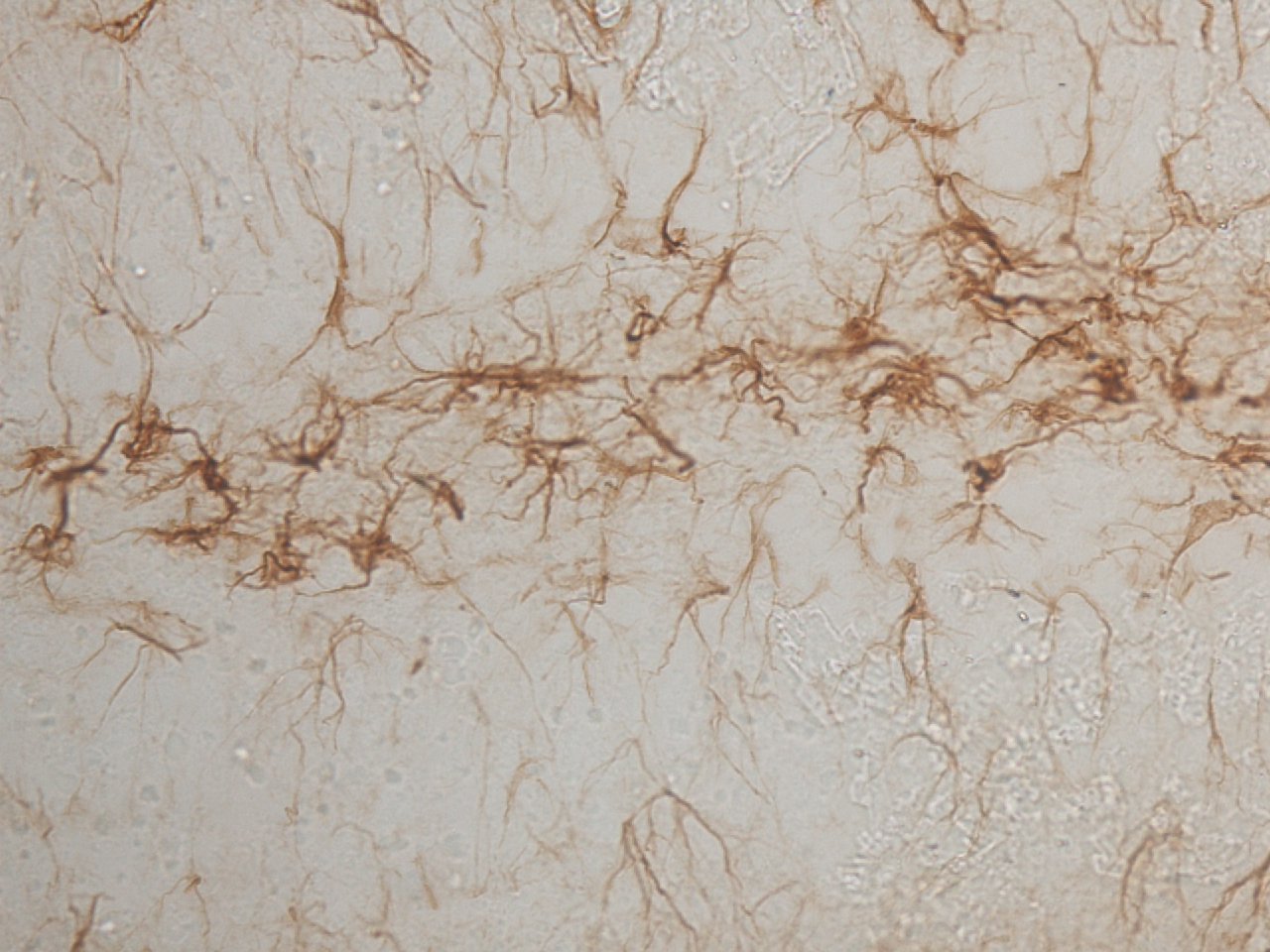

Supplement: Supplementary file 7 — Source data Fig. 6 [file 44321_2024_79_MOESM7_ESM.zip › Figure 6/6C/Hippocampus HI+Sema.TIF]

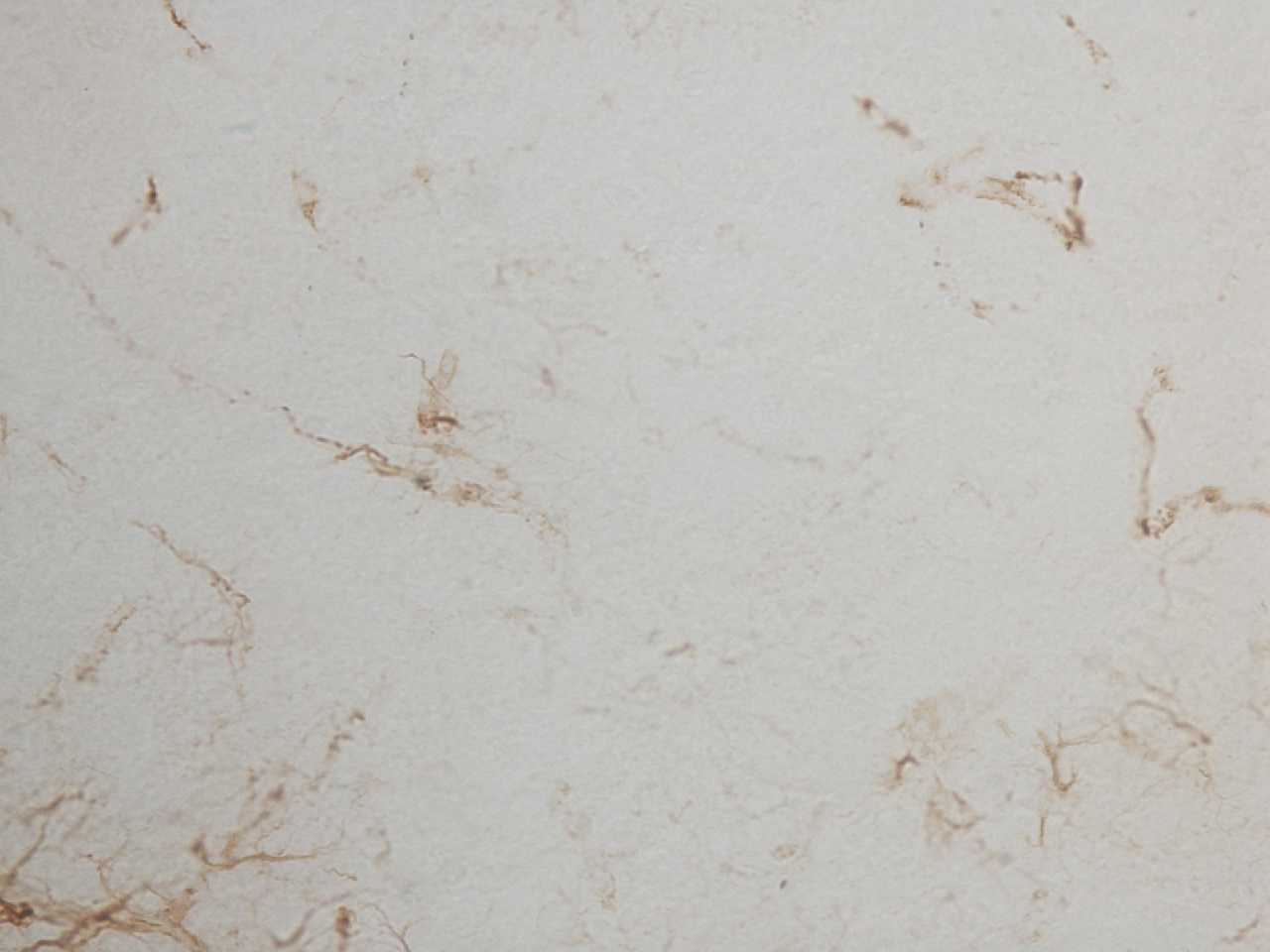

Supplement: Supplementary file 7 — Source data Fig. 6 [file 44321_2024_79_MOESM7_ESM.zip › Figure 6/6C/Cortex HI+Sal.TIF]

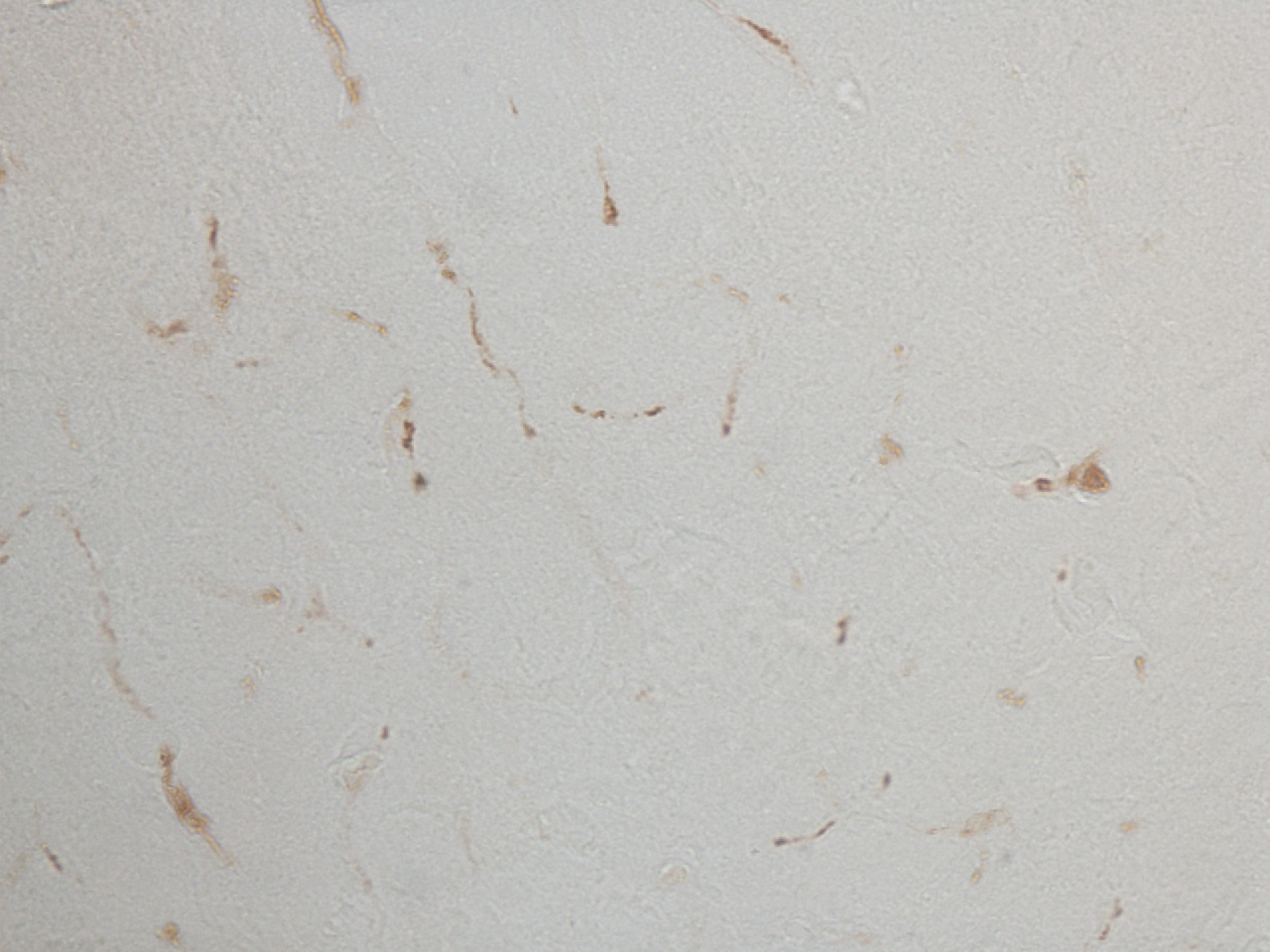

Supplement: Supplementary file 7 — Source data Fig. 6 [file 44321_2024_79_MOESM7_ESM.zip › Figure 6/6C/Cortex HI+Ex-4.TIF]

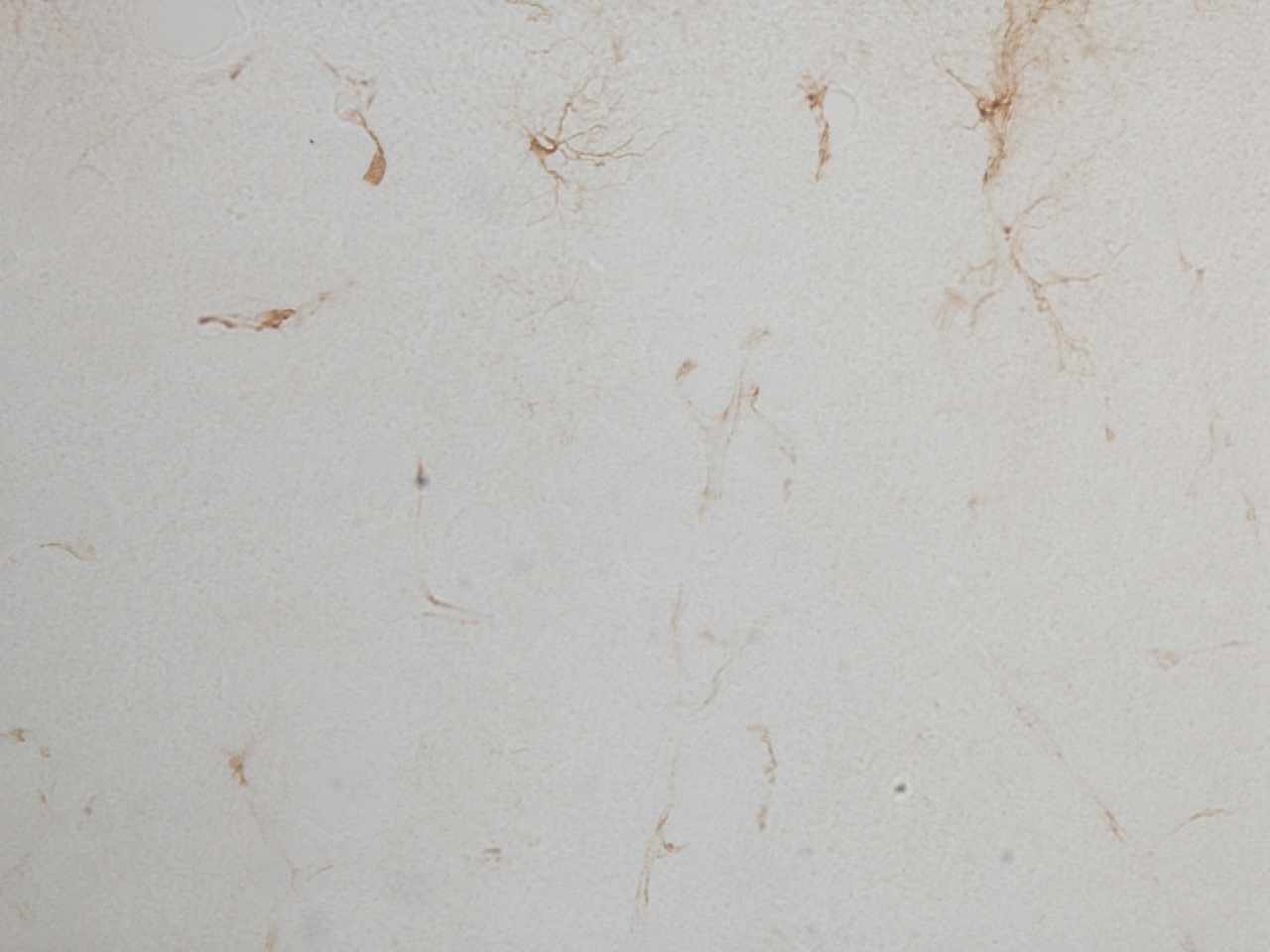

Supplement: Supplementary file 7 — Source data Fig. 6 [file 44321_2024_79_MOESM7_ESM.zip › Figure 6/6C/Cortex Saline.TIF]

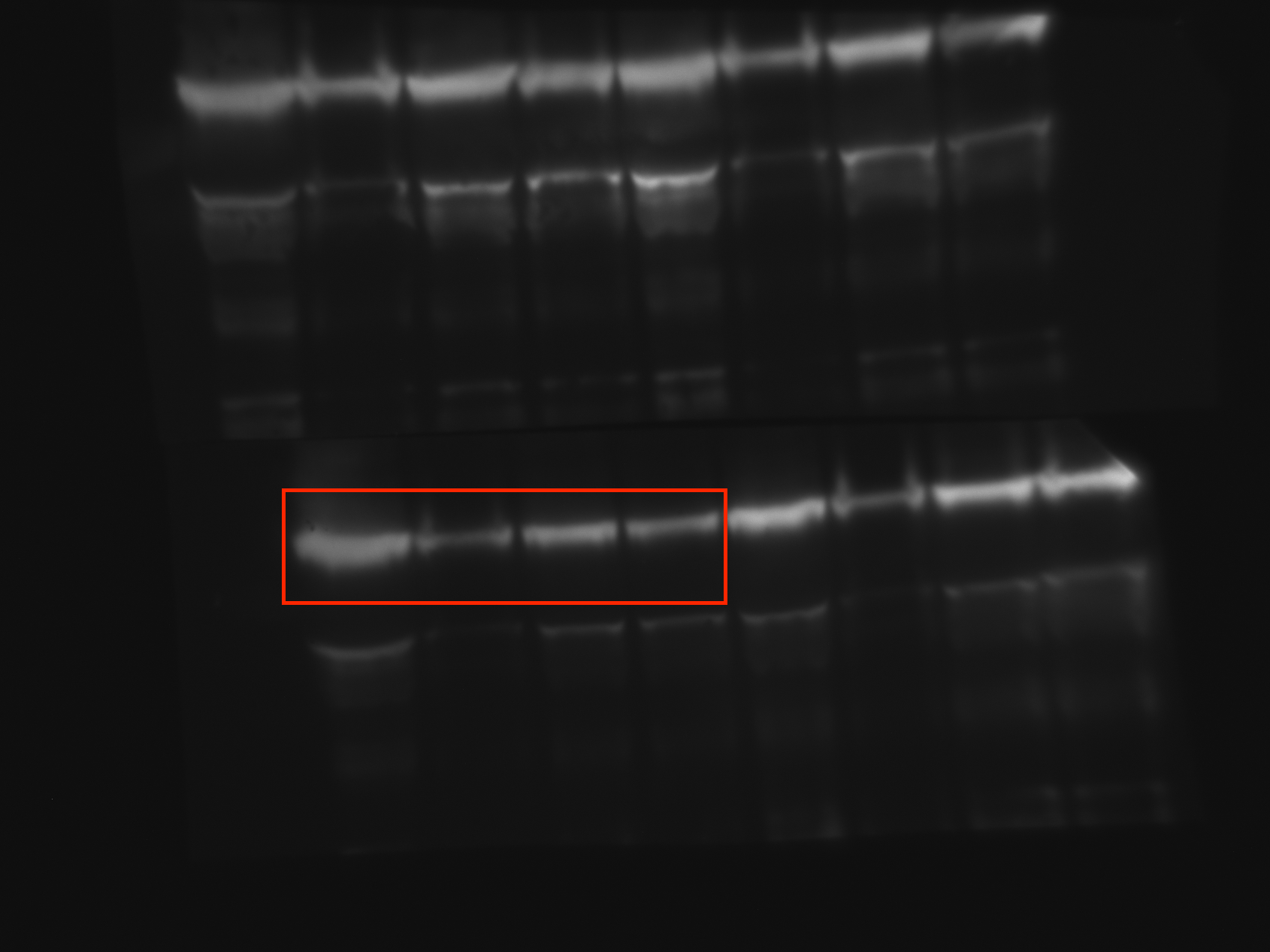

Supplement: Supplementary file 9 — Source data Fig. 8 [file 44321_2024_79_MOESM9_ESM.zip › Figure 8/8G/p-Akt.TIF]

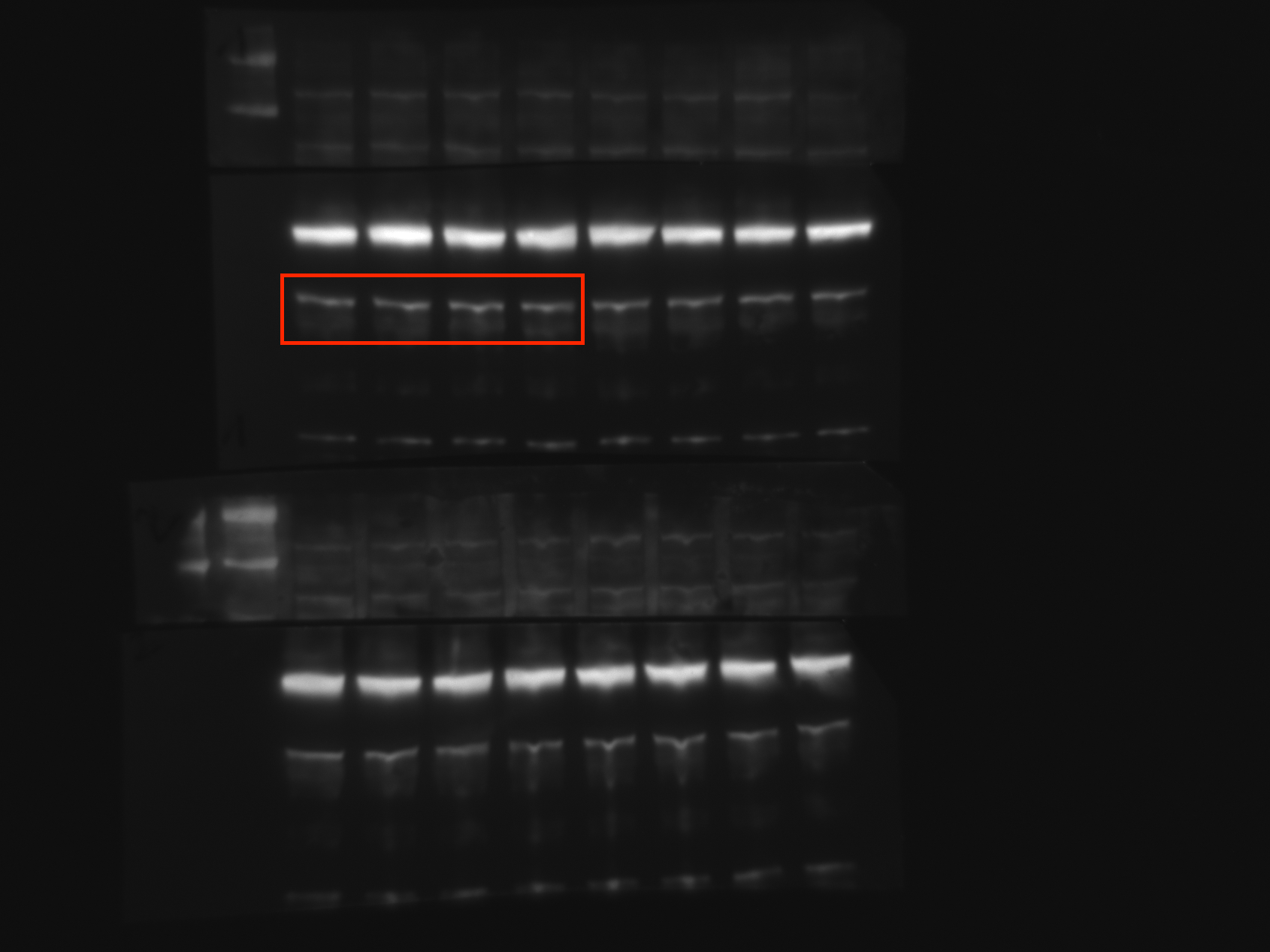

Supplement: Supplementary file 9 — Source data Fig. 8 [file 44321_2024_79_MOESM9_ESM.zip › Figure 8/8G/P13K.TIF]

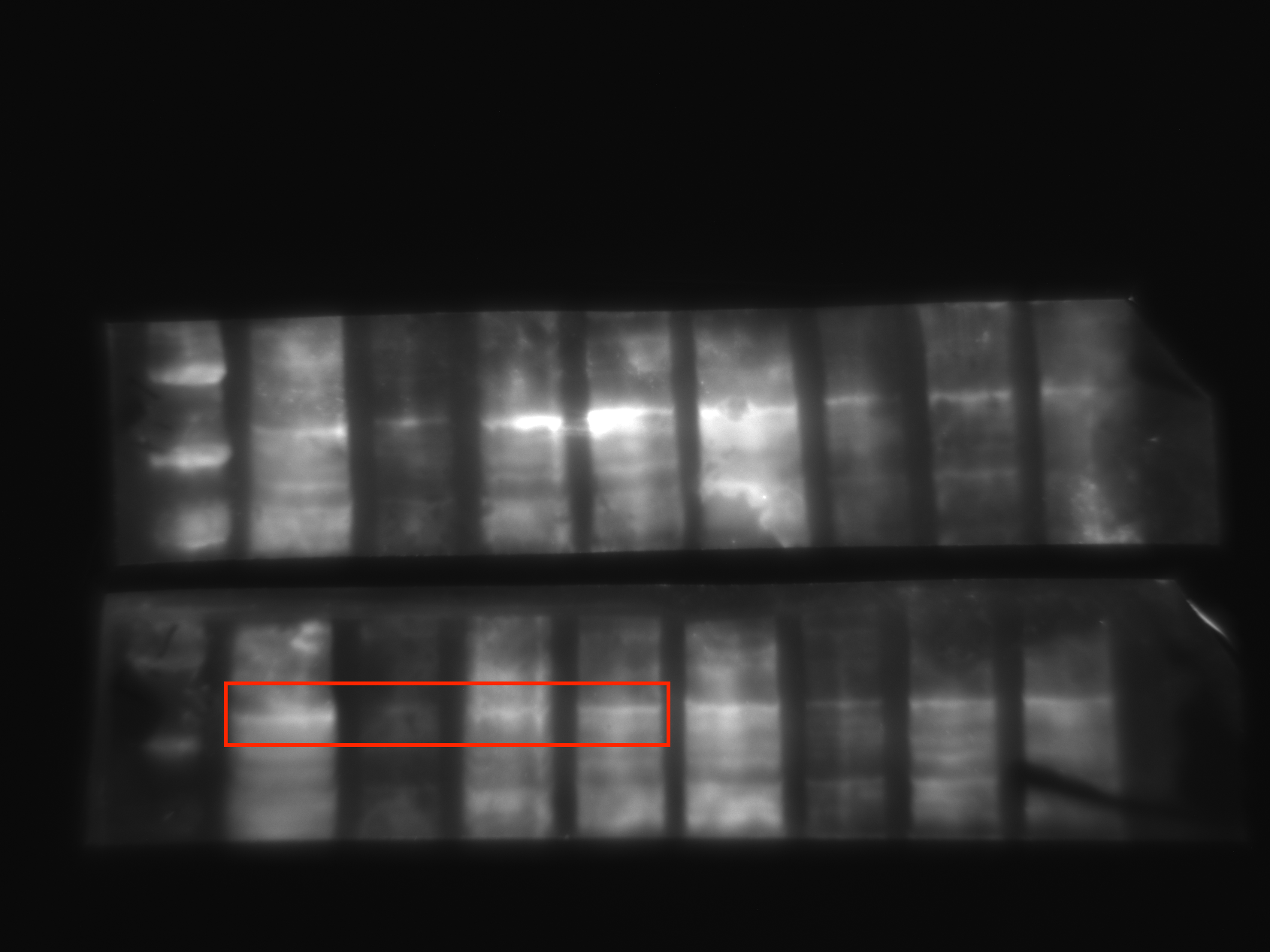

Supplement: Supplementary file 9 — Source data Fig. 8 [file 44321_2024_79_MOESM9_ESM.zip › Figure 8/8G/P-P13K.TIF]

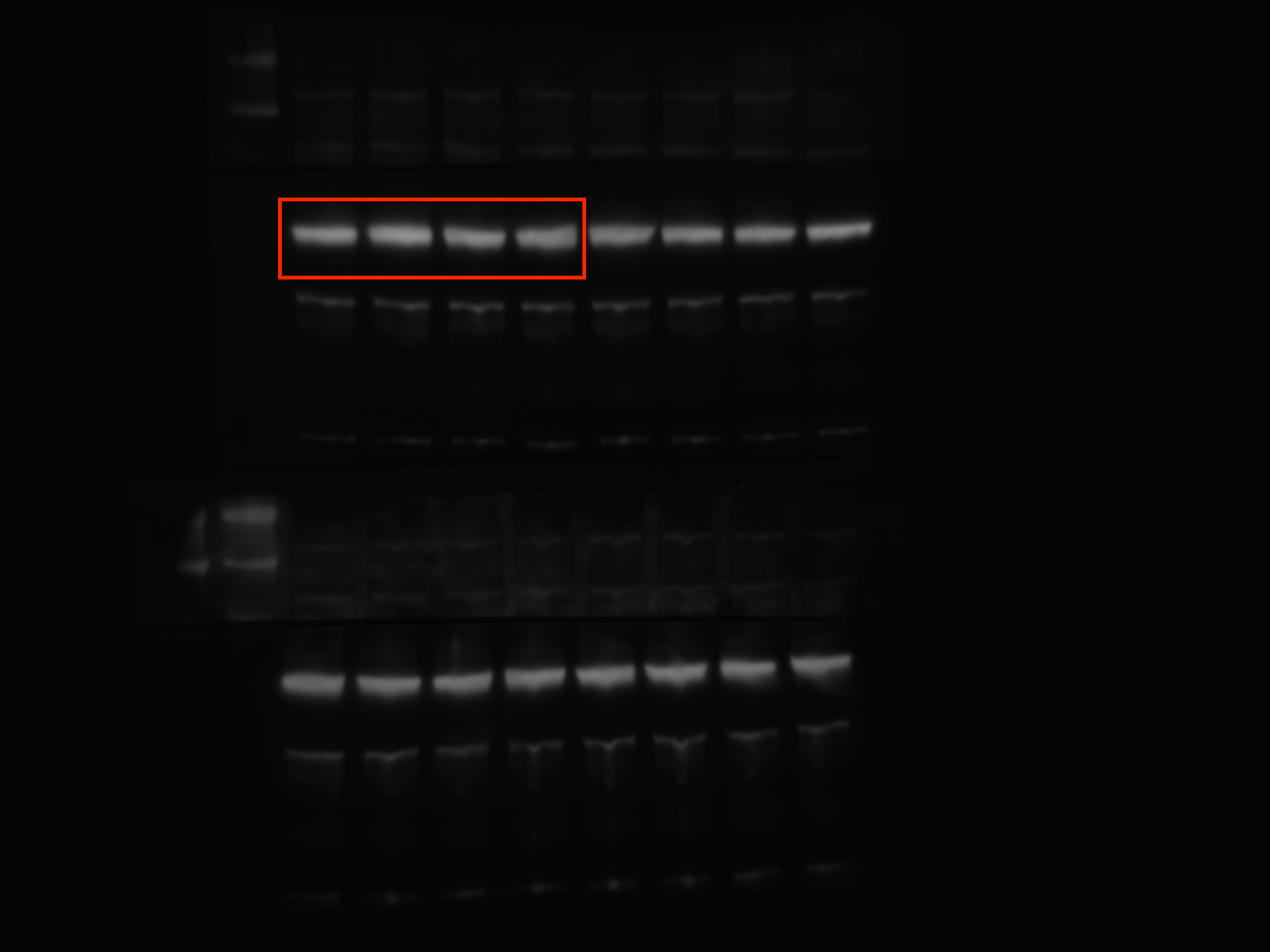

Supplement: Supplementary file 9 — Source data Fig. 8 [file 44321_2024_79_MOESM9_ESM.zip › Figure 8/8G/AKT.TIF]

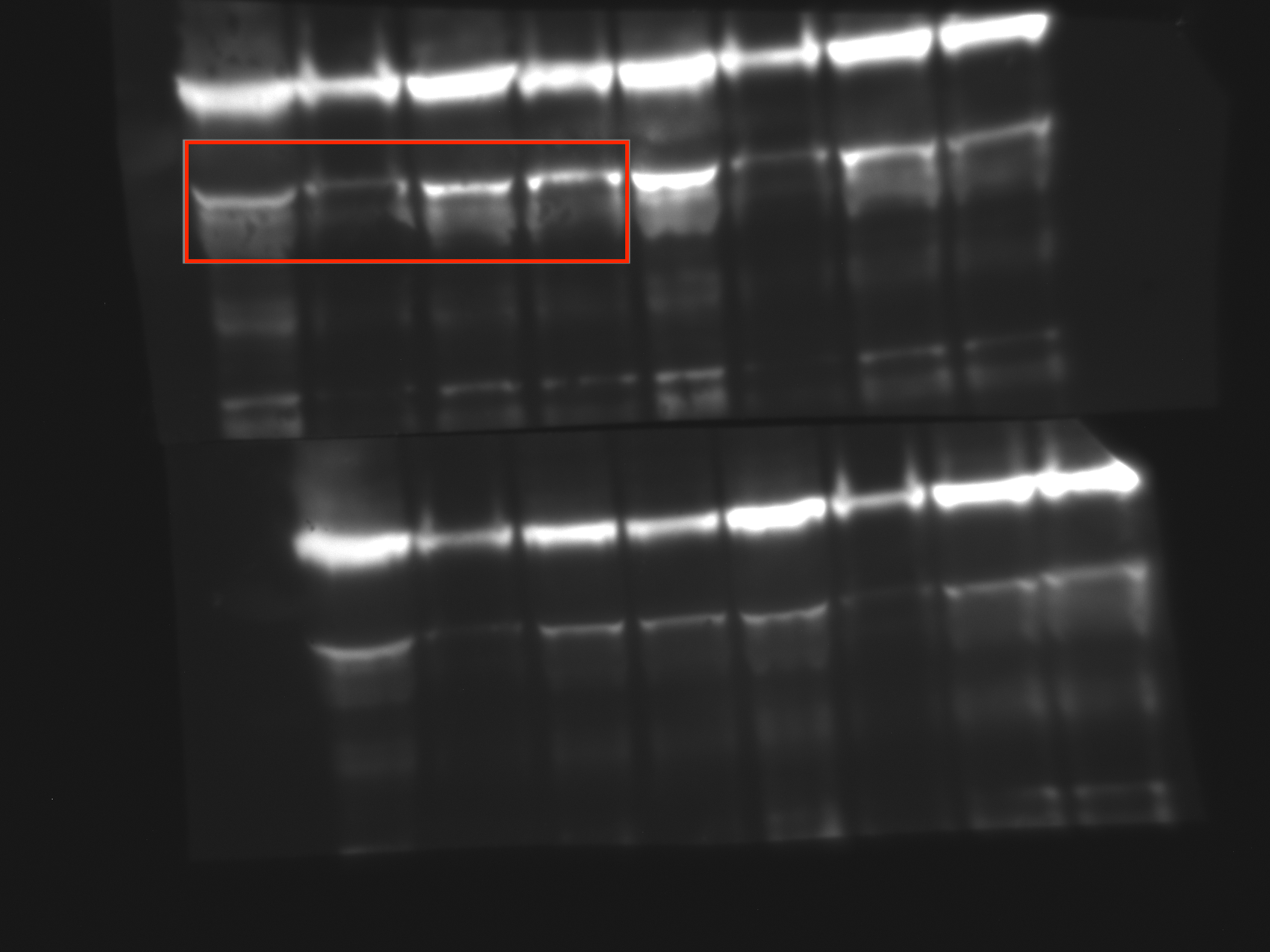

Supplement: Supplementary file 9 — Source data Fig. 8 [file 44321_2024_79_MOESM9_ESM.zip › Figure 8/8D/P-Creb S133.tiff]

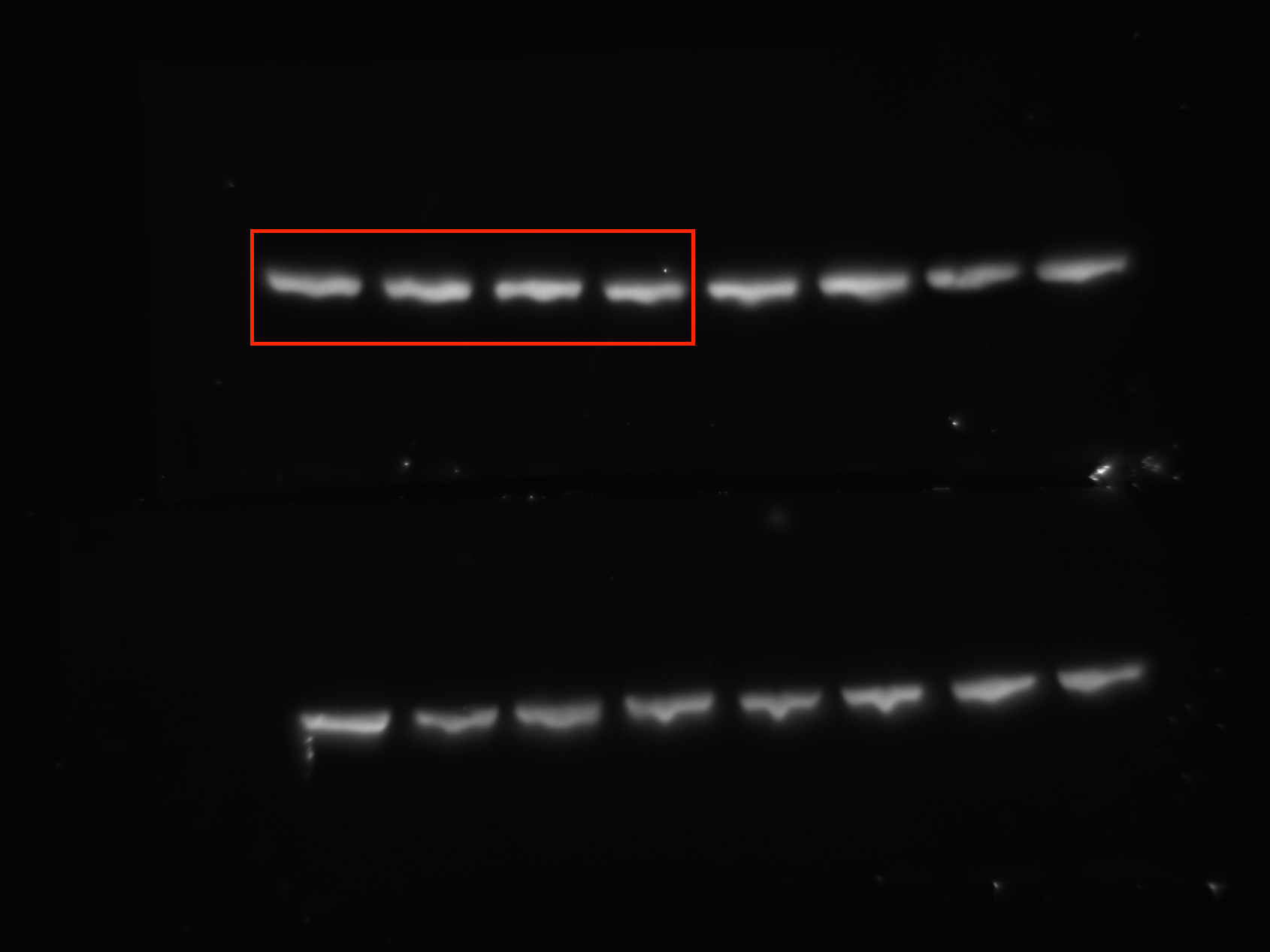

Supplement: Supplementary file 9 — Source data Fig. 8 [file 44321_2024_79_MOESM9_ESM.zip › Figure 8/8D/B-actin.TIF]

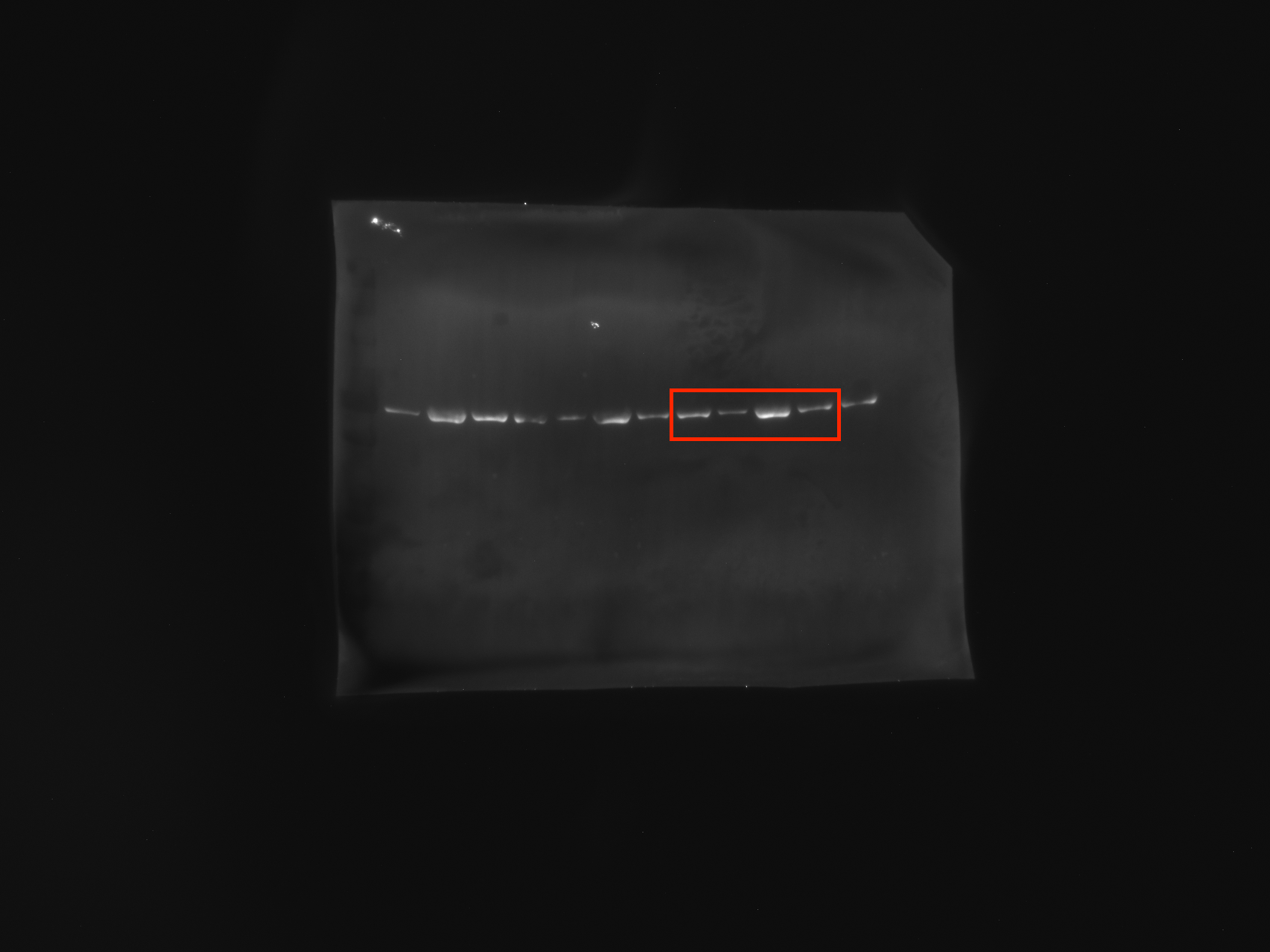

Supplement: Supplementary file 9 — Source data Fig. 8 [file 44321_2024_79_MOESM9_ESM.zip › Figure 8/8D/p-GSK3B S9.TIF]

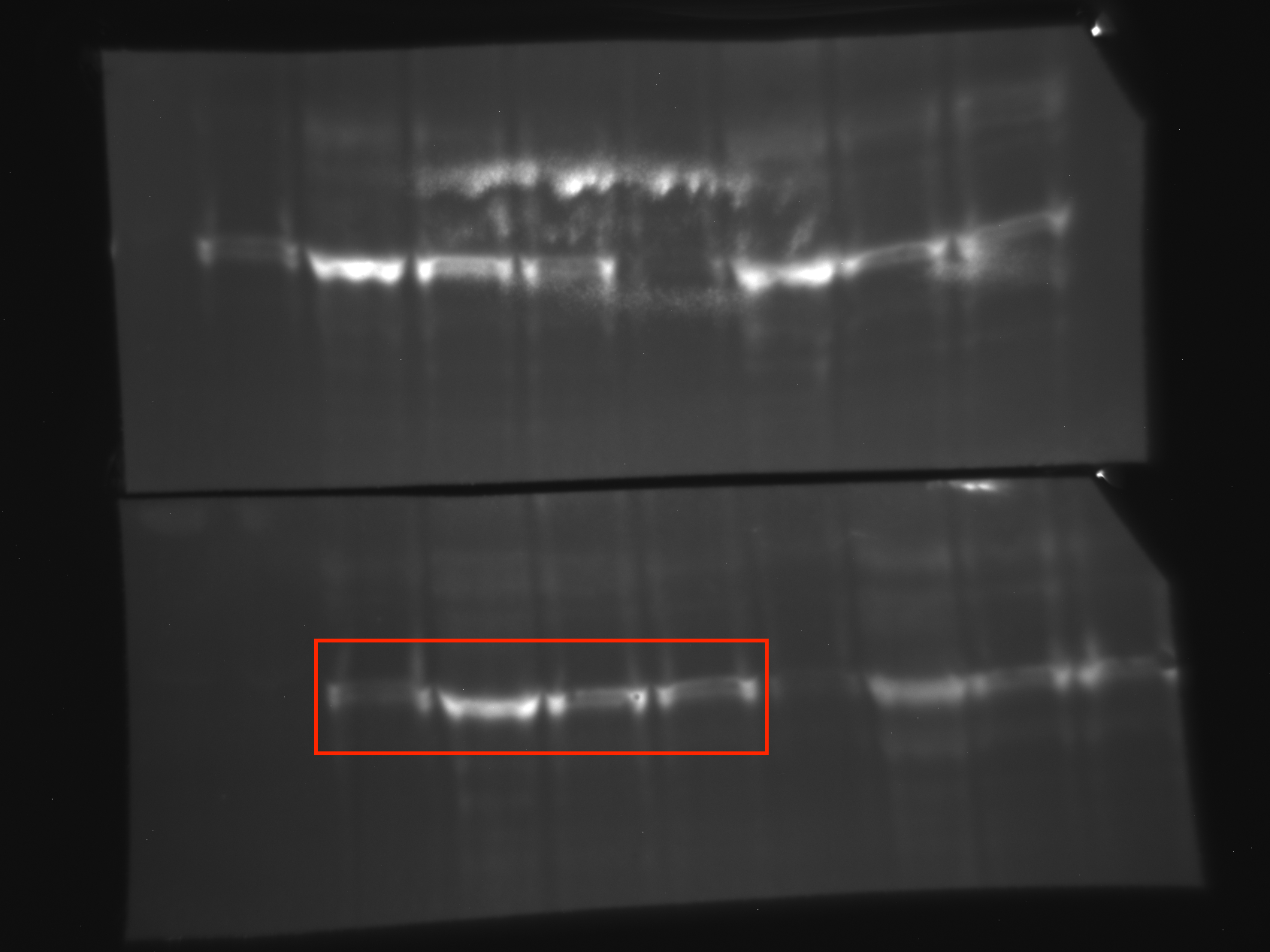

Supplement: Supplementary file 9 — Source data Fig. 8 [file 44321_2024_79_MOESM9_ESM.zip › Figure 8/8D/p-GSK3B Y216.TIF]
