# Supplementary material for: Diabetes drugs activate neuroprotective pathways in models of neonatal hypoxic-ischemic encephalopathy
Source: EMBO Mol Med. 2024 May 23;16(6):5. doi: 10.1038/s44321-024-00079-1 (PMC11178908; doi:10.1038/s44321-024-00079-1)
Supplement: Supplementary file 11 — Expanded View Figures [file 44321_2024_79_MOESM11_ESM.pdf]

Expanded View Figures

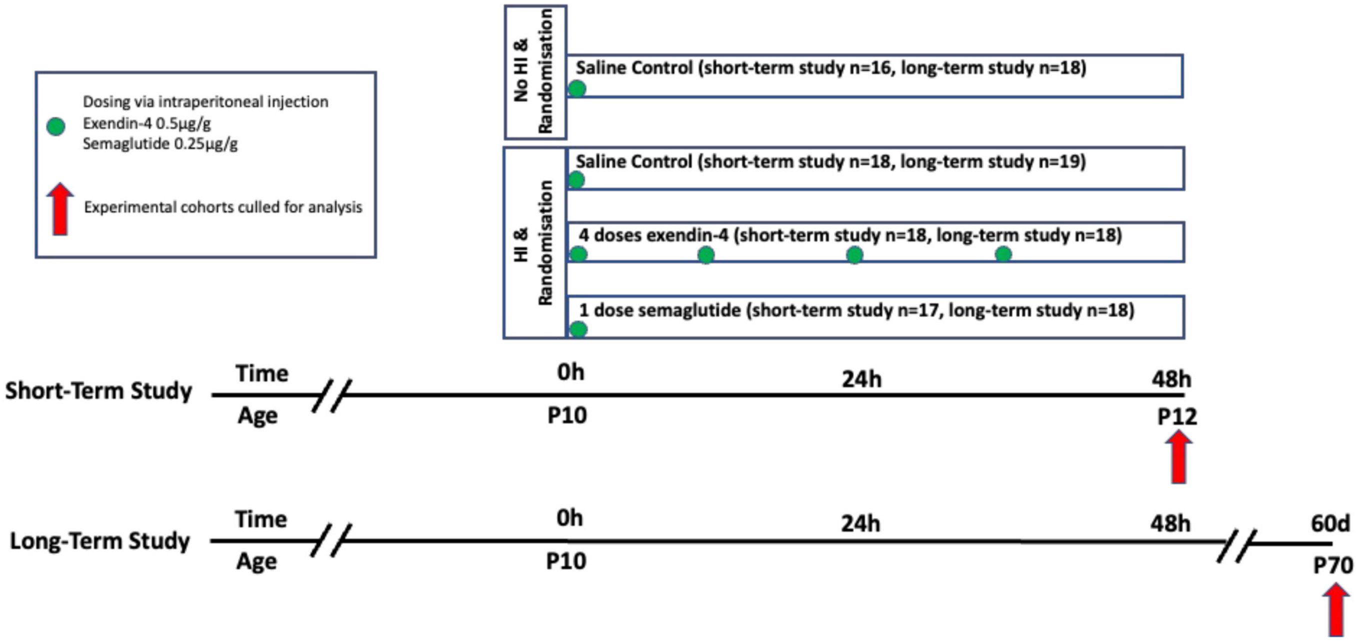

Figure EV1. In vivo HI and GLP1-R agonist short-term and long-term studies experimental design.

© The Author(s)

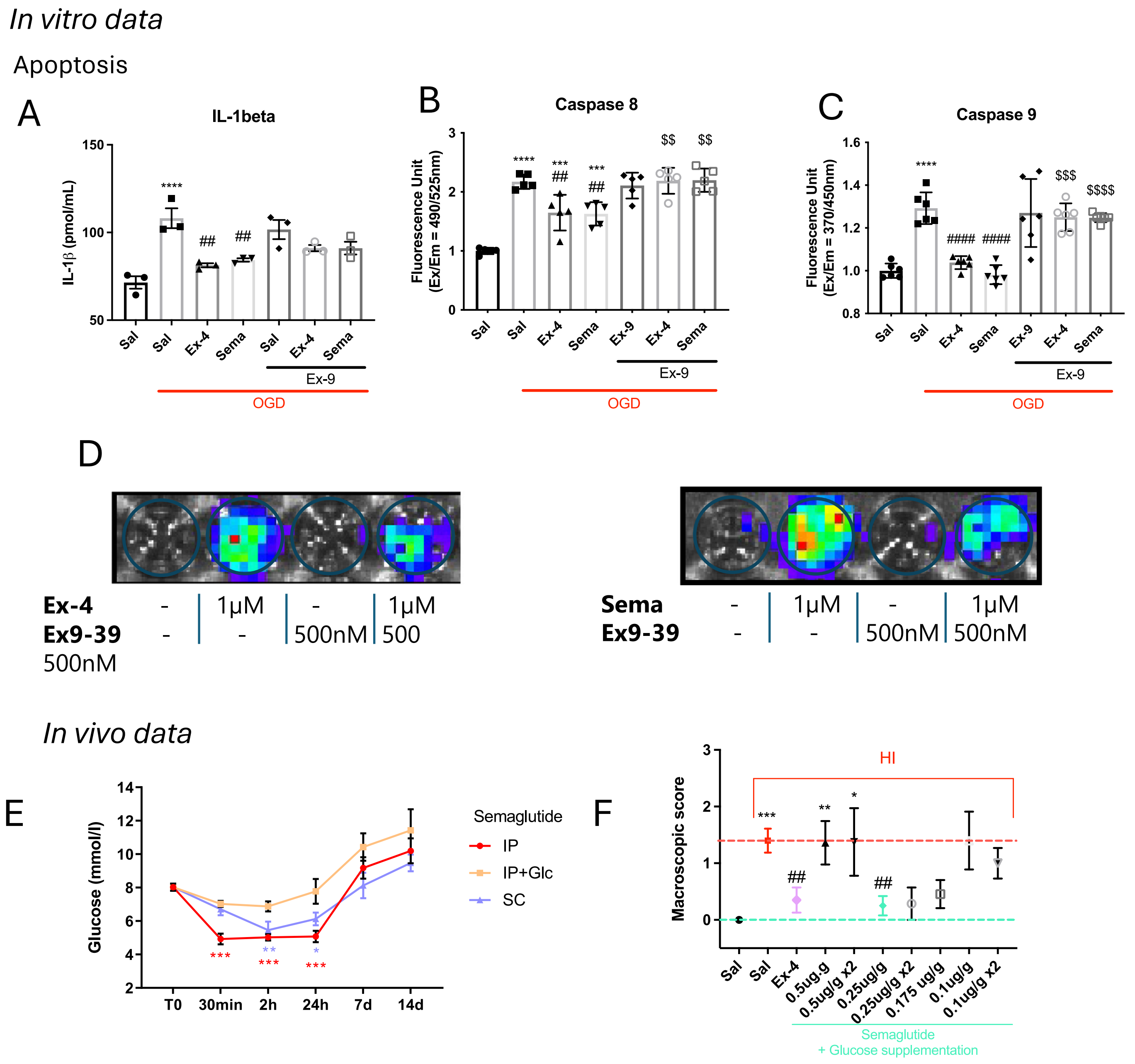

Figure EV2. Complementary results for in vitro study post-OGD and in vivo study post-HIE.

(**A**) Evaluation of proinflammatory marker IL-1β using qPCR in the in vitro neuronal cells exposed to OGD following treatment with exendin-4 or semaglutide, and in combination with exendin-9 (Ex-9) ($n = 3$ for all experimental groups). (**B**) Apoptotic proteins caspase 8 ($n = 5$ for all experimental groups) and (**C**) caspase 9 ($n = 6$ for all experimental groups) were measured using caspase assays. (**D**) Luminescent images of GLP1R/pCRE-luciferase/CHO-K1 cells treated with 0–1 µM of exendin-4 or semaglutide with or without antagonist, exendin9-39 (500 nM). (**E**) Determination of optimal routes of administration for avoiding hypoglycaemia with glucose measurement after semaglutide IP, semaglutide SC and semaglutide + glucose regimens ($n = 4$ per experimental group for each time point). (**F**) Dosing experiment with evaluation of macroscopic score after HI and treatment to determine optimal dose for semaglutide in the 10 groups: Sal ($n = 20$), HI + Sal ($n = 44$), HI + Ex-4 ($n = 20$), HI + 0.5 µg/g semaglutide ($n = 18$), HI + 2× 0.5 µg/g semaglutide ($n = 8$), HI + 0.25 µg/g semaglutide ($n = 16$), HI + 2× 0.25 µg/g semaglutide ($n = 7$), HI + 0.175 µg/g semaglutide ($n = 11$), HI + 0.1 µg/g semaglutide ($n = 5$), HI + 2× 0.1 µg/g semaglutide ($n = 11$). Data information: Error bars indicate mean ± SEM. Statistical analysis was performed using an ordinary one-way (**A–C**) or two-way (**E, F**) analysis of variance (ANOVA) corrected for using Tukey's multiple comparisons test. * or # or $, $p < 0.05$; ** or ## or $$, $p < 0.01$; *** or ### or $$$, $p < 0.001$; **** or #### or $$$$, $p < 0.0001$. * to compare with control (Sal) and # to compare with OGD group (Sal + OGD). $ symbol used to compare GLP1-R agonists treatment with corresponding treatment in combination with exendin-9.

© The Author(s)

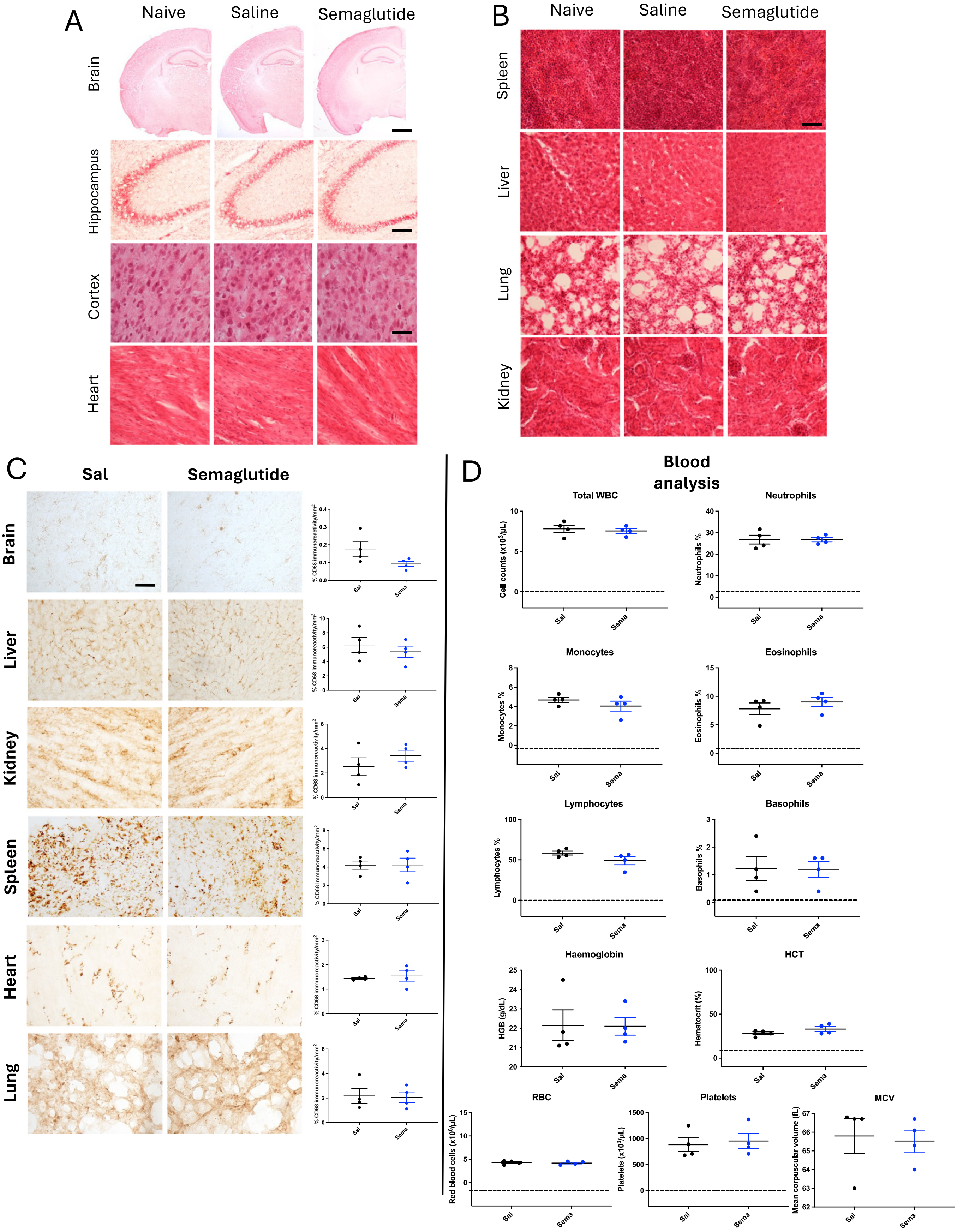
A
Naive
Saline
Semaglutide
Brain
Hippocampus
Cortex
Heart
B
Naive
Saline
Semaglutide
Spleen
Liver
Lung
Kidney
C
Sal
Semaglutide
Brain
Liver
Kidney
Spleen
Heart
Lung
% CD68 immunoreactivity/mm²
Sal
Sema
D
Blood analysis
Total WBC
Cell counts (x10³/µL)
Neutrophils
Neutrophils %
Monocytes
Monocytes %
Eosinophils
Eosinophils %
Lymphocytes
Lymphocytes %
Basophils
Basophils %
Haemoglobin
HGB (g/dL)
HCT
Hematocrit (%)
RBC
Red blood cells (x10⁶/µL)
Platelets
Platelets (x10³/µL)
MCV
Mean corpuscular volume (fL)

 © The Author(s)

Figure EV3. Toxicity assessment after semaglutide treatment.

(**A**, **B**) Representative images of Haematoxylin & Eosin staining showing no adverse effects observed in tissue architecture in naïve P10 mice following a single high dose of semaglutide (0.25 µg/g) in the brain or major visceral organs compared to controls. (**C**) CD68 staining and quantification revealed no macrophage activation or infiltration compared to controls. (**D**) Blood analysis also showed no significant changes in response to high dose semaglutide ($n = 4$ for all experimental groups). Scale bar: 0.1 cm (brain), 100 µm (Hippocampus), 60 µm (Cortex, Heart, Spleen, Liver, Lung, Kidney and all CD68 staining images in panel **C**). Data information: error bars indicate mean ± SD, Statistical analysis was performed using a t-test. *$p < 0.05$, **$p < 0.01$, ***$p < 0.001$, ****$p < 0.0001$. * to compare with control and group treated with semaglutide.

© The Author(s)

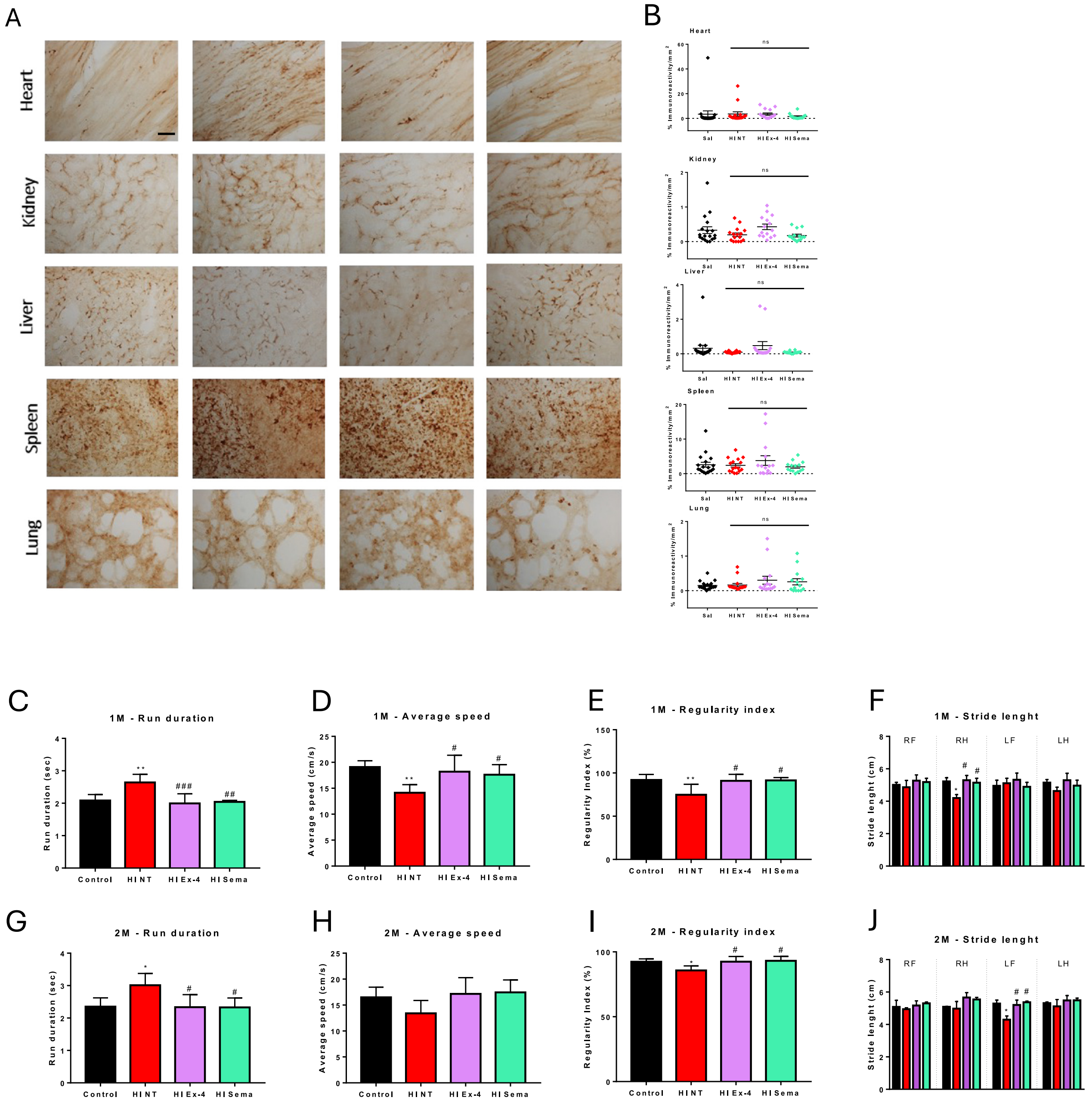

Figure EV4. Complementary results for long-term in vivo study post HI.

(**A**, **B**) No significant microglia activation or infiltration was observed or measured in sections from organ harvested from saline controls (Sal, $n = 18$), HI non-treated (HINT, $n = 16$), HI treated with Exendin-4 (Ex-4, $n = 15$) and HI treated with Semaglutide animals (Sema, $n = 14$). Scale bar: 60 µm. Locomotor functions were evaluated at 1 and 2 months post-HI with Catwalk and revealed significant exendin-4 and semaglutide induced improvements at 4 weeks (**C–F**) and 8 weeks (**G–J**) of age compared with the age-matched hypoxic-ischaemic group for various parameters: (**C**, **G**) run duration, (**D**, **H**) average speed, (**E**, **I**) regularity index, and (**F**, **J**) stride length (RF = right front, RH = right hind, LF = left front, LH = left hind) in saline controls (Control, 1 M $n = 18$, 2 M $n = 15$), HI non-treated (HINT, 1 M $n = 16$, 2 M $n = 13$), HI treated with Exendin-4 (Ex-4, 1 M $n = 14$, 2 M $n = 12$) and HI treated with Semaglutide (Sema, 1 M $n = 21$, 2 M $n = 22$). Data information: Each n represents an individual mouse. Error bars indicate mean ± SD. Statistical analysis performed using an ordinary one-way analysis of variance (ANOVA) with a Dunnett's multiple comparisons test. * or #, $p < 0.05$; ** or ##, $p < 0.01$; *** or ###, $p < 0.001$; **** or ####, $p < 0.0001$. * to compare with control and # to compare with HI non-treated (NT) group.

 © The Author(s)

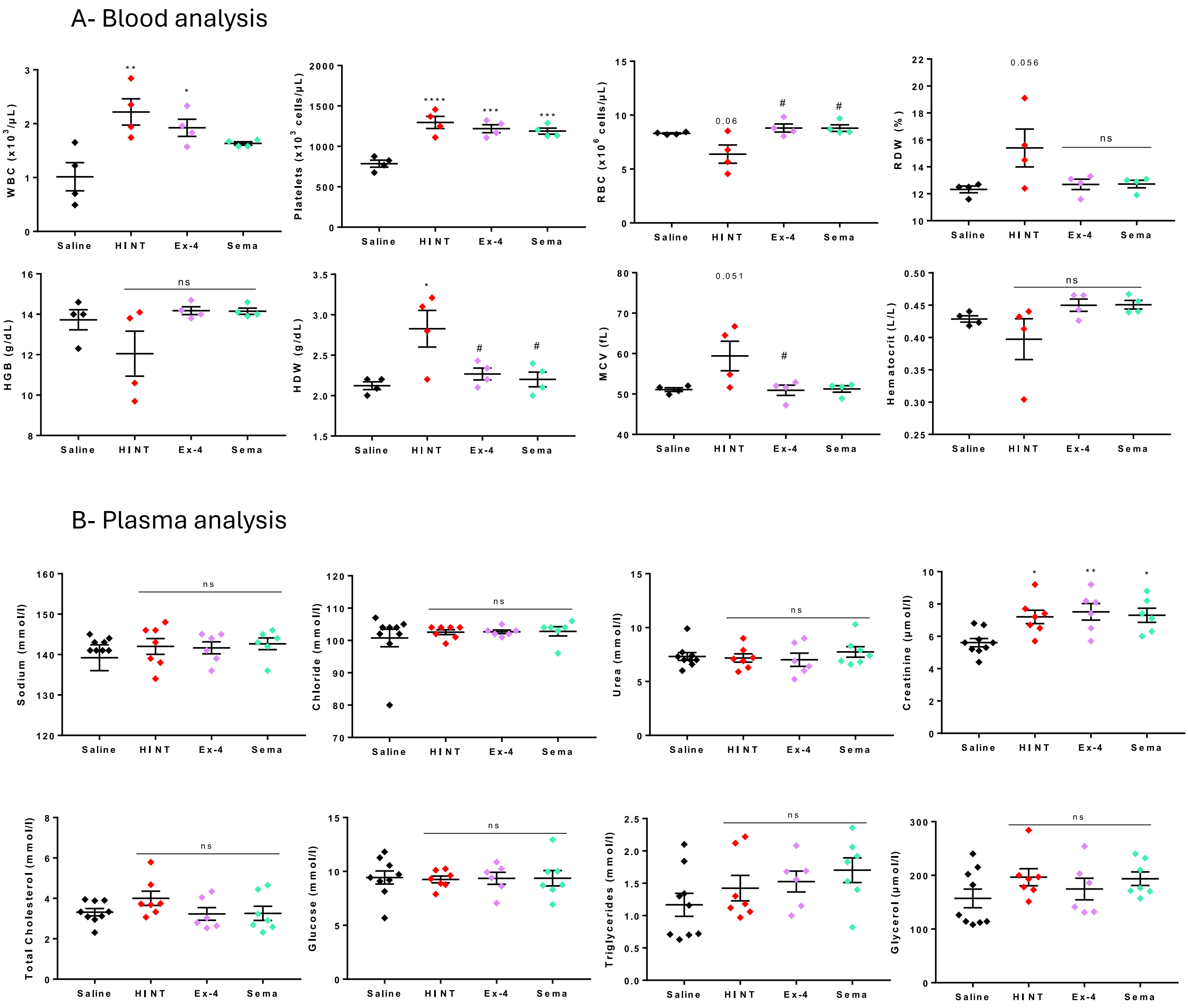

Figure EV5. Blood and plasma analysis 8 weeks post-insult.

(**A**) Blood analysis from the long-term 8 weeks study showed HI resulted in a significant increase in WBCs, platelets and haemoglobin distribution widths (HDWs) and a trend towards decreased RBCs, haemoglobin (HGB), increased red cell distribution widths (RDWs) and MCVs. Treatment with GLP1-R agonists improved the RBC, RDW, HGB, HDW and MCV counts ($n = 4$ per experimental group). (**B**) Analysis of plasma parameters (sodium, chloride, urea, creatinine, total cholesterol, glucose, triglycerides, glycerol levels) in saline controls (Saline, $n = 9$), saline-treated HI animals (HINT, $n = 7$), HI treated with Exendin-4 (Ex-4, $n = 6$) and HI treated with Semaglutide (Sema, $n = 7$), which showed no significant difference between groups except for a statistically significant increase in creatinine in all HI groups. Data information: Each n represents an individual mouse. Error bars indicate mean ± SD. Statistical analysis performed using an ordinary one-way analysis of variance (ANOVA) with a Dunnett's multiple comparisons test. * or #, $p < 0.05$; ** or ##, $p < 0.01$; *** or ###, $p < 0.001$; **** or ####, $p < 0.0001$. * to compare with control and # to compare with HI non-treated (NT) group.

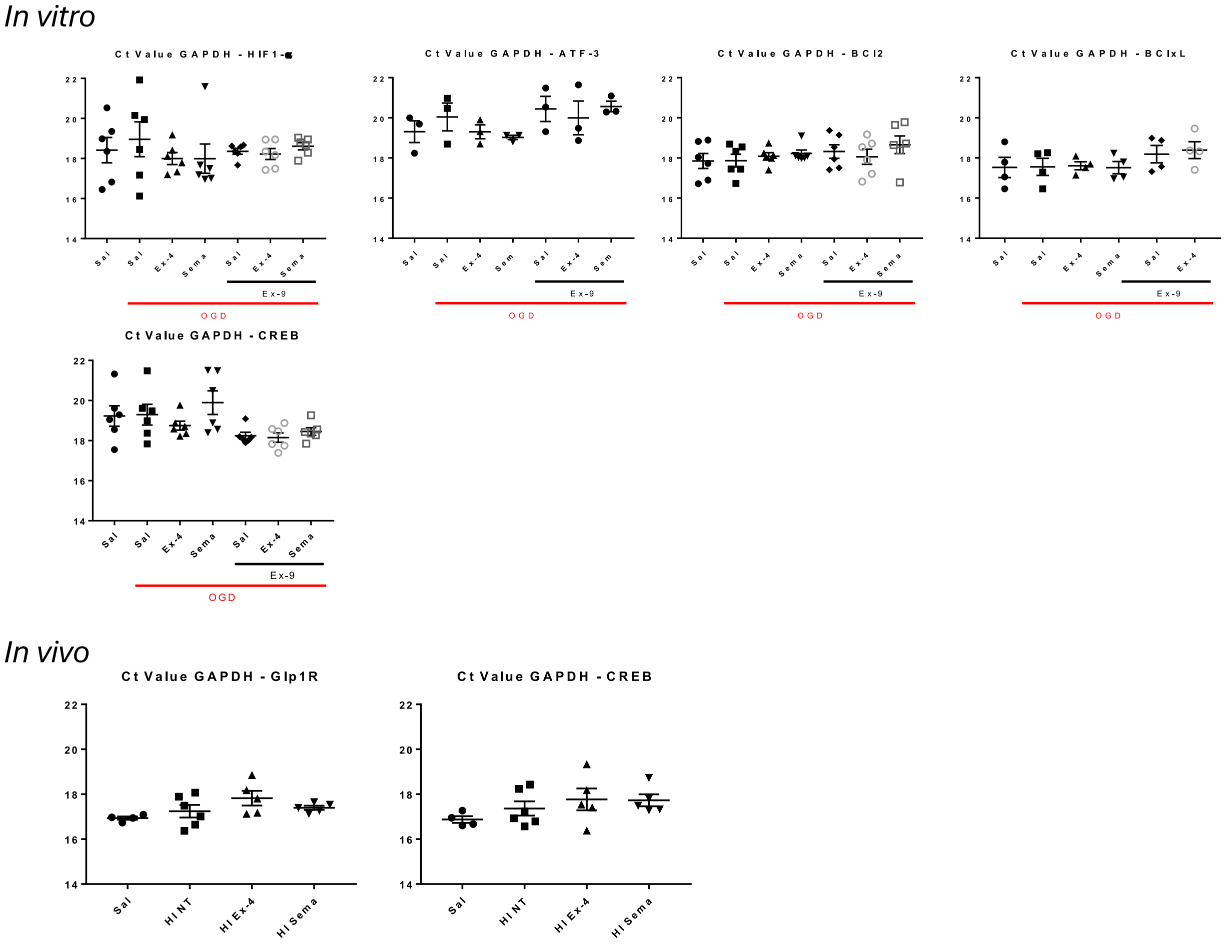

Figure EV6. All qPCR control ct values are shown from the study.

The GAPDH Ct value for various markers investigated by qPCR in the in vitro neuronal cells exposed to OGD following treatment with exendin-4 or semaglutide, and in combination with exendin-9: HIF-1-α ($n = 6$ for all experimental group), ATF-3 ($n = 3$ for all experimental groups), Bcl-2 ($n = 6$ for all experimental groups), Bcl-xL ($n = 4$ for all experimental groups) and CREB ($n = 6$ for all experimental groups). In addition, the Ct values for qPCR into Glp1R and CREB in the HIE model are also shown(Sal $n = 4$, HINT $n = 6$, HI Ex-4 $n = 5$ and HI Sema $n = 5$). Data information: error bars indicate mean ± SEM. Each n represents an individual sample.

 © The Author(s)
